# Manganese-catalyzed hydroboration of carbon dioxide and other challenging carbonyl groups

**DOI:** 10.1038/s41467-018-06831-9

**Published:** 2018-10-30

**Authors:** Christina Erken, Akash Kaithal, Suman Sen, Thomas Weyhermüller, Markus Hölscher, Christophe Werlé, Walter Leitner

**Affiliations:** 1 Max-Planck-Institute for Chemical Energy Conversion, Stiftstraße 34-36, 45470 Mülheim an der Ruhr, Germany; 2 Institut für Technische und Makromolekulare Chemie, RWTH Aachen University, Worringer Weg 2, 52074 Aachen, Germany

## Abstract

Reductive functionalization of the C=O unit in carboxylic acids, carbonic acid derivatives, and ultimately in carbon dioxide itself is a challenging task of key importance for the synthesis of value-added chemicals. In particular, it can open novel pathways for the valorization of non-fossil feedstocks. Catalysts based on earth-abundant, cheap, and benign metals would greatly contribute to the development of sustainable synthetic processes derived from this concept. Herein, a manganese pincer complex [Mn(Ph_2_PCH_2_SiMe_2_)_2_NH(CO)_2_Br] (**1**) is reported to enable the reduction of a broad range of carboxylic acids, carbonates, and even CO_2_ using pinacolborane as reducing agent. The complex is shown to operate under mild reaction conditions (80–120 °C), low catalyst loadings (0.1–0.2 mol%) and runs under solvent-less conditions. Mechanistic studies including crystallographic characterisation of a borane adduct of the pincer complex (**1**) imply that metal-ligand cooperation facilitates substrate activation.

## Abstract (web summary)

Catalytic reduction of CO_2_ and biomass derivatives is of great current interest from a sustainable chemistry perspective. Here, the authors report a manganese pincer complex able to reduce under mild conditions carboxylic acids, carbonates and even carbon dioxide using pinacolborane as reducing agent.

## Introduction

The catalytic reduction of the C=O unit in carboxylic acids (RCO_2_H), carbonic acid derivatives (O=C(OR)_2_), and ultimately carbon dioxide (CO_2_) itself is of great current interest for the development of sustainable chemical value chains. It can enable novel synthetic pathways through manipulation of functional groups in fine chemical and pharmaceutical synthesis in line with the principles of Green Chemistry^1–3^. In particular, such transformations are of paramount importance also in the utilization of alternative feedstocks derived from biomass^4–6^ or from carbon dioxide^7–10^. The use of molecular complexes of first-row transition metals has gained increasing attention as potential catalysts for reduction reactions in this context^11–22^. These metals are in most cases more readily available at lower costs and in some cases also more environmentally benign as compared to noble metals from the platinum group that would be applied typically in such transformations. Manganese is emerging as a particularly interesting metal component, as it fulfills all the criteria above and has been described to exhibit catalytic activity especially in form of so-called pincer complexes for reduction of carbonyl units in challenging substrates in most recent studies^23–30^. In the present paper, we report a Mn pincer complex that enables catalytic reductive functionalization for a wide range of structurally divers substrates through hydroboration of free carboxylic acids, cyclic five- and six-membered carbonates, linear carbonates, and even carbon dioxide.

## Results

### Synthesis of Mn complex 1

**Fig. 1:**
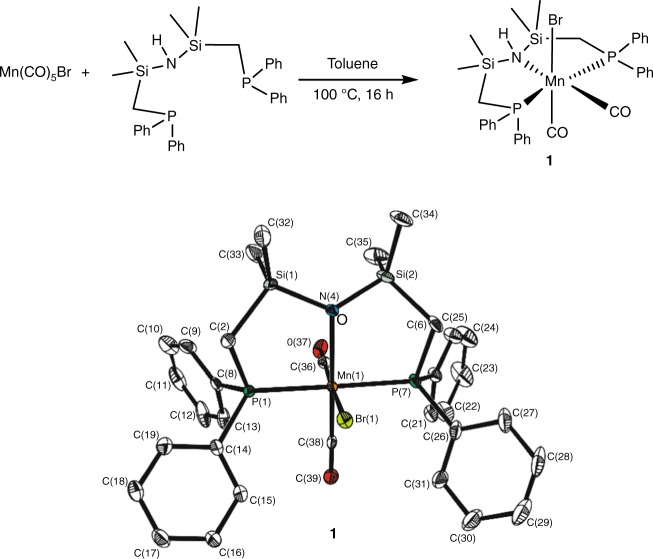
Reaction scheme and molecular structure of complex **1**. Overall geometry: distorted octahedral. Selected angles and atom distances: [P(1)-Mn(1)-P(7) = 173.47°], [C(36)-Mn(1)-C(38) = 86.14°]. The Mn center is embedded in the P,N,P-pincer framework with bond lengths in the typical range [(Mn(1)-P(1) = 229.97 pm; Mn(1)-N(4) = 227.4 pm; Mn(1)-P(7) = 230.22 pm), (Si(1)-N(4) = 177.0 pm), (N(4)-Si(2) = 177.1 pm)]; and both P-Mn-N angles very close to 90° (P(1): 88.32° and P(7): 87.91°, respectively)

The complex [Mn(Ph_2_PCH_2_SiMe_2_)_2_NH(CO)_2_Br] (**1**) was synthesized by treatment of Mn(CO)_5_Br with one equivalent of 1,3-bis((diphenyl-phosphino)methyl)tetramethyldi-silazane^31^ in toluene at 100 °C for 16 h (Fig. 1). Complex **1** was isolated as a yellow powder with 90% yield. All spectroscopic data are in the expected range (see Supplementary Information for details) and single crystals suitable for X-ray diffraction were obtained by slow diffusion of hexane into a concentrated solution of **1** in dichloromethane.

### Reduction of carboxylic acids

Exploring the potential of complex **1** as a catalyst, we were very pleased to find that exhibited excellent activity in the reduction of a broad range of carboxylic acids with pinacolborane in the presence of KO^*t*^Bu as base under neat conditions (Table 1). The hydroboration of free carboxylic acid remains otherwise largely elusive to date. Very recently, Gunanathan’s group reported the Ruthenium-catalyzed hydroboration of carboxylic acids with pinacolborane to alkyl boronate esters that could be hydrolyzed to the corresponding alcohols^32^. Notably, this noble-metal complex is currently the only catalytic system that has been reported for this transformation except for complex **1**.

**Table 1:**
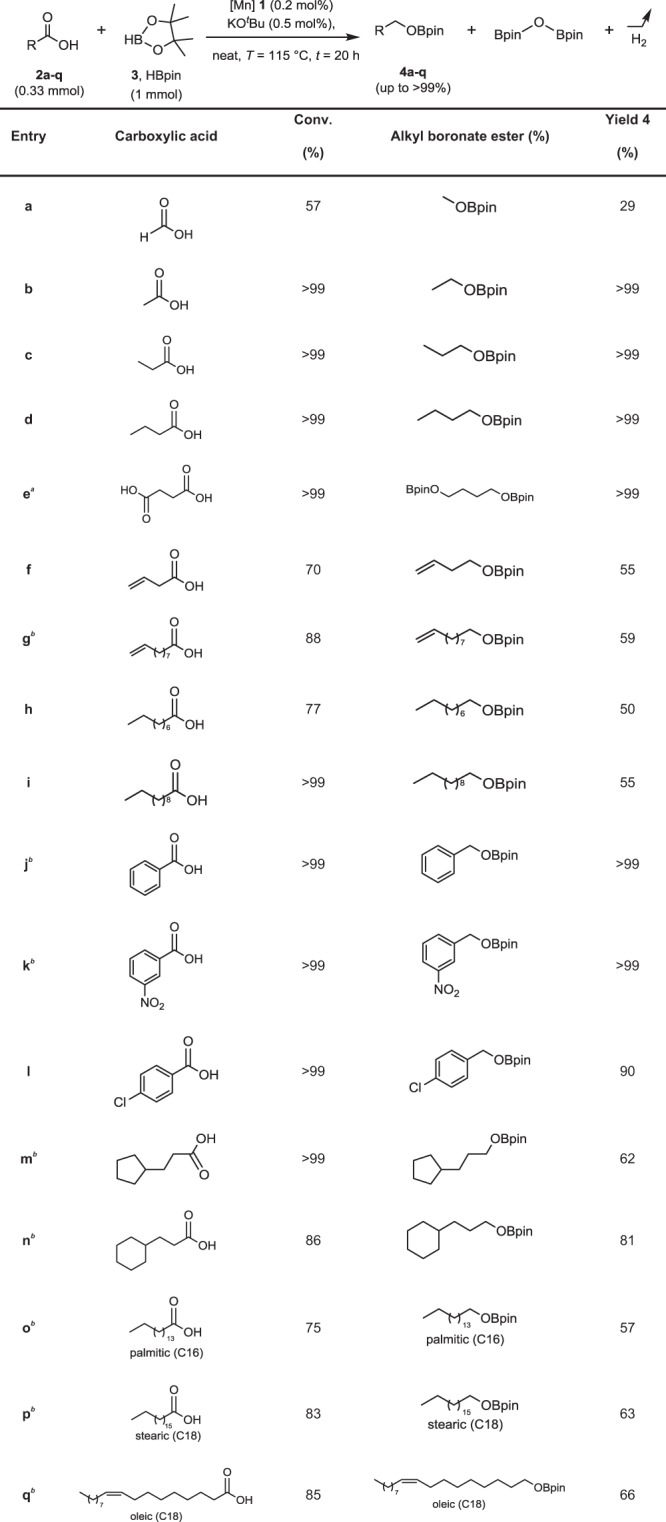
Hydroboration of carboxylic acids using Mn complex 1 as catalyst

Yields were determined by ^1^H NMR analysis using mesitylene as internal standard. Reaction conditions: **2** (0.33 mmol), **1** (0.2 mol%), KO^*t*^Bu (0.5 mol%) and **3** (1.0 mmol) at 115 °C for 20 h. **a**, Modified conditions: **2e** (0.33 mmol), **1** (0.2 mol%), KO^*t*^Bu (0.5 mol%) and **3** (2.0 mmol) at 115 °C for 36 h. **b**, Modified conditions: **2** (0.33 mmol), **1** (0.2 mol%), KO^*t*^Bu (0.5 mol%) and **3** (1.0 mmol) at 115 °C for 24 h

The reaction conditions of the standard protocol shown in Table 1 were derived from a detailed screening using acetic acid (**2b**) as benchmark substrate (see Supplementary Table 1 and Supplementary Table 2). Both the manganese complex and the base were found to be essential components for significant conversion (i.e., for **2b** 15% of yield in the absence of base and 30% of yield in the absence of **1**). NEt_3_ could also be used as base with good performance (90%, 24 h), while NaOH gave substantially lower yields (39%, 24 h). The use of solvents was found detrimental independent of their polarity or donor strength. The reaction reached >99% yield within 20 h at catalyst loadings of 0.2 mol% and higher and within 24 h even at 0.1 mol%.

Aliphatic acids (**2a–i, 2m–q**) were converted in very good to excellent yields independent of the chain length, including even the C1 compound formic acid (**2a**). Carbon-carbon double bonds were fully tolerated and not reduced (**2f–g, 2q**). Benzoic acid (**2j**) also reacted smoothly. The reduction was very selective even in the presence of chloro (**2l**), or nitro groups (**2k**), albeit yield was somewhat lower under standard reaction conditions in the latter case. The biomass-derived platform chemical succinic acid (**2e**) was hydroborated in good yields upon adjustment of the stoichiometric ratio of the pinacolborane. The excellent results obtained with saturated and unsaturated aliphatic carboxylic acids prompted us to apply the Mn-catalyzed hydroboration also to fatty acids. Both saturated (**2o–p**) and unsaturated (**2q**) fatty acids were readily converted to the corresponding alkyl boronates in 57–66% yield after 24 h. The integrity of the double bonds and their stereochemical arrangement was fully retained during this transformation.

### Reduction of carbonates

The selective reduction of carbonic acid esters is very challenging as these compounds are kinetically very stable toward hydride addition. In fact, organic carbonates are frequently used as a solvent in catalytic reduction reactions^33^. Reduction of carbonates has gained additional interest as these compounds can be prepared from CO_2_ and CO, leading to their reduction to methanol derivatives^34,35^. Cyclic carbonates can be envisaged also as protecting groups or synthetic precursors for the corresponding diols that are simultaneously liberated upon reductive cleavage^36,37^. The hydroboration of carbonates has not been described up to now, however.

Ethylene carbonate (**5a**) was chosen as a model substrate to screen for catalytic activity and identify a set of standard reaction conditions with catalyst **1** (see the Supplementary Information and Supplementary Table 3 for details). Reaction of ethylene carbonate (1 mmol) with the stoichiometric equivalent of pinacolborane (3 mmol) in the presence of complex **1** (0.1 mol%) and NaO^*t*^Bu (0.3 mol%) at 90 °C revealed formation of the boronate ester ethylene glycol already in 77% yield after 4 h. Methyl boronate (**4a**), the actual reduction product from the central carbon of the carbonate group, was detected in 62% yield. No significant conversion was observed in absence of either **1** or base. Up to 97% yield could be achieved at higher loadings of complex **1** and base (0.25 and 0.4 mol%, respectively). The yield could be increased to practically the same level of 95% also at the initial lower loadings upon prolonging the reaction time to 8 h. These conditions were chosen to assess the substrate scope of this catalytic transformation.

As summarized in Table 2, various carbonates including cyclic and linear structures were reduced to the corresponding hydroboration product. All reactions were carried out under solvent-free conditions. Five-membered ring carbonates (**5a–c**) gave excellent yields between 95 and 97% similar to ethylene carbonate. Interestingly, the 1,2-carbonate of glycerol (**5d**), which is the byproduct of the industry of vegetable oils, was also reduced to the corresponding boronate ester with 88% yield. Complex **1** also proved to be highly active for the catalytic hydroboration of six-membered ring carbonates. 1,3-dioxan-2-one (**5e**) and 5-methyl-5-propyl-1,3-dioxan-2-one (**5g**) gave 98% and 94% yield, respectively. The significantly lower yield of 39% obtained with 5,5-dimethyl-1,3-dioxan-2-one (**5f**) is probably due to the low solubility of the substrate in pinacolborane.

**Table 2:**
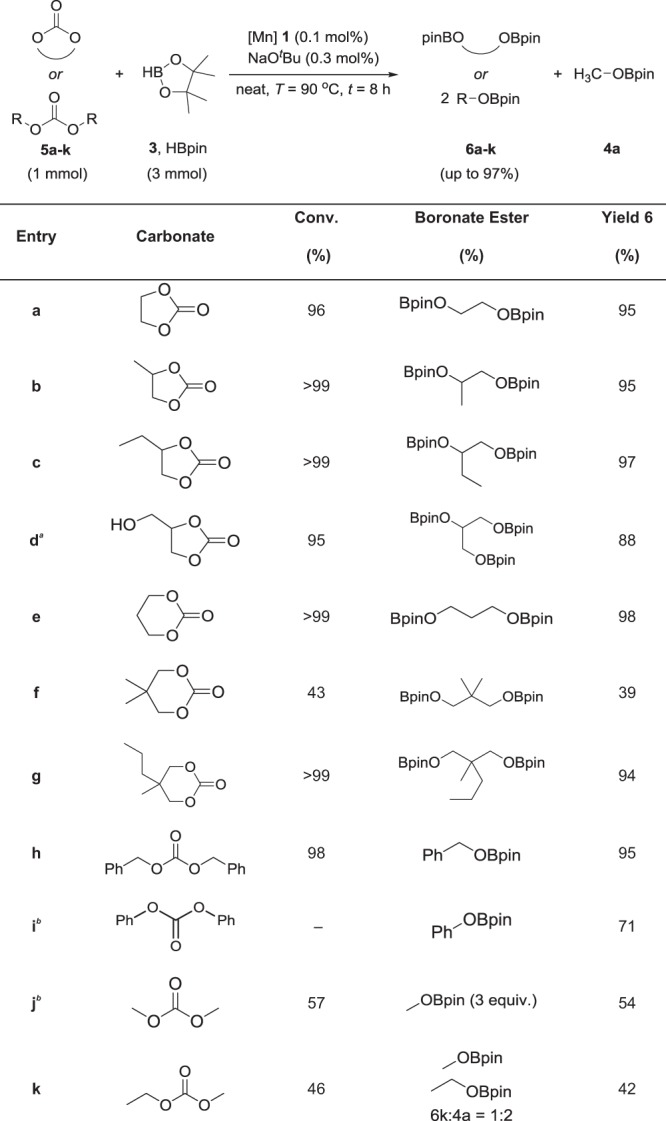
Hydroboration of carbonates using Mn complex 1 as catalyst

Yields of **6** were determined by ^1^H NMR analysis using mesitylene as internal standard. **a**, Modified conditions: **5** (1 mmol), **1** (0.1 mol%), NaO^*t*^Bu (0.3 mol%), and **3** (4 mmol) at 90 °C for 8 h. **b**, Modified conditions: Yield of **4a** was determined by ^1^H NMR analysis using mesitylene as internal standard

Linear carbonates are considered to be even more resistant to reduction than their cyclic congeners. Gratifyingly, complex **1** was able to catalyze the hydroboration of dibenzyl carbonate (**5h**) with similar efficiency yielding two equivalents of benzyl boronate in a total of 95% yield. Diphenyl carbonate (**5i**) showed 71% yield to the corresponding boronate ester. Lower conversion was achieved with dimethyl (**5j**) and ethyl methyl carbonate (**5k**) which may be due to their solely aliphatic functionality.

### Reduction of CO_2_

The high catalytic activity of the manganese complex **1** in the formation of methyl boronate (**4a**) from formic acid as well as from various carbonates prompted us to investigate its ability to reduce carbon dioxide directly under the same conditions. The reduction of CO_2_ to methanol or methanol derivatives is a field of great current interest within strategies to utilize waste carbon dioxide as feedstock at the interface of the energy and chemistry sectors (e.g., carbon capture and utilization, CCU, and power-to-X concepts)^38–41^. The hydroboration of CO_2_ has been explored in this context using noble and non-noble metal catalyst^19,21,42–44^. In 2010, Guan et al. reported the first hydroboration of CO_2_ to yield methoxy boronate using a nickel pincer complex showing a turnover number (moles product per moles catalyst, TON) of 495^45^. Several noble metal complexes were also reported for the reduction of CO_2_ to methoxy boronate, but TONs were even lower^44,46^. There is no report currently where the hydroboration of CO_2_ was achieved using a manganese-based catalyst.

Gratifyingly, complex **1** proved able to catalyze the formation of methyl boronate (**4a**) using carbon dioxide as feedstock under rather mild conditions similar to those applied for the other substrates. Control experiments demonstrated again that both the Mn complex and the base were required. Initial experiments were carried out using 0.036 mol% of complex **1** and 0.1 mol% of NaO^*t*^Bu dissolved in 2.76 mmol of pinacolborane under ambient pressure of CO_2_ (from a balloon). The presence of **4a** in solution was verified by NMR spectroscopy, amounting to 76% based on the HBpin employed after 14 h at 90 °C (Table 3, entry 1). These data correspond to a TON of 697 representing already the highest productivity observed for this transformation even under the non-optimized conditions. The reaction occurs with high selectivity and no other C1 compounds such as formaldehyde-derivatives, CO or methane are observed. Complex **1** shows activity also in toluene or THF as solvent, albeit with lower yields. Doubling the amount of catalyst and base amount under solvent-free conditions increased product formation to 83% yield, but of course on expense of the TON. The highest TON was achieved at the original loadings increasing the reaction temperature to 100 °C. This led to nearly quantitative conversion of HBpin (**3**) giving MeOBpin (**4a**) in 96% yield corresponding a TON of 883.

**Table 3:**
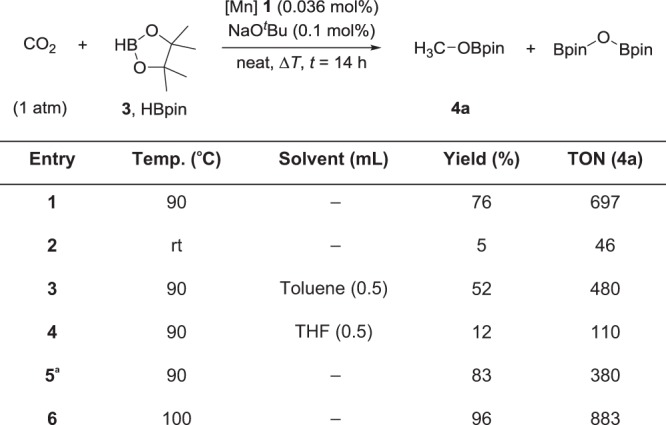
Hydroboration of CO_2_ using Mn complex **1** as catalyst

Yields were determined by ^1^H NMR analysis using mesitylene as internal standard. **a**, Modified conditions: CO_2_ (1 atm), **1** (0.072 mol%), NaO^*t*^Bu (0.2 mol%) and **3** (2.76 mmol) at 90 °C for 14 h

### Mechanistic studies

**Fig. 2:**
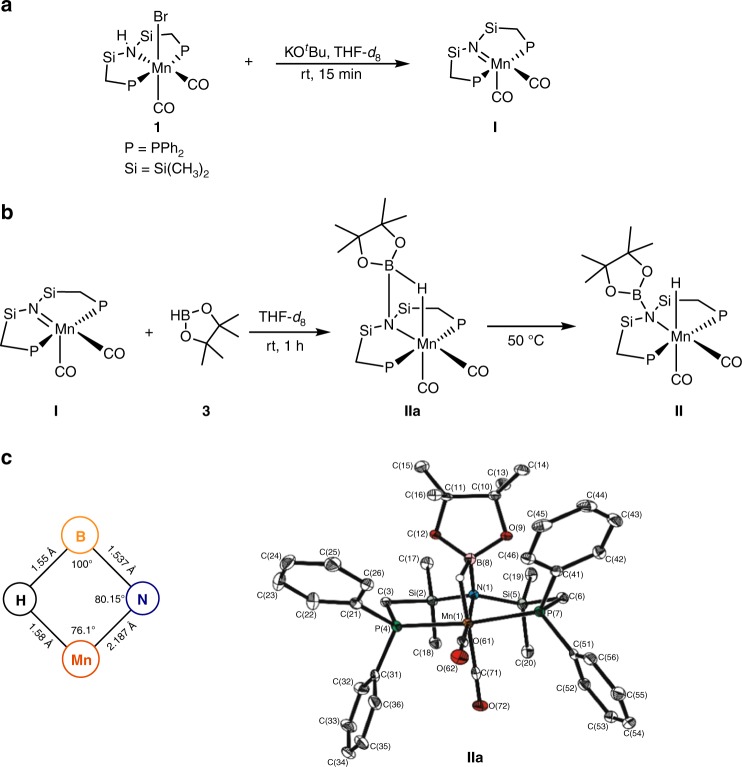
Generation of potential reaction intermediates and molecular structure of **IIa**. Overall geometry for **IIa**: distorted octahedral. Selected angles and atom distances: [P(4)-Mn(1)-P(7) = 172.38°], [C(61)-Mn(1)-C(71) = 86.73°], [N(1)-Mn(1)-P(4) = 88.85°], [N(1)-Mn(1)-P(7) = 88.90°], (Mn(1)-N(1) = 218.7 pm), (Mn(1)-P(4) = 226.93 pm), (Mn(1)-P(7) = 226.95 pm), (N(1)-Si(2) = 178.1 pm), (N(1)-Si(5) = 177.9 pm)

The reactivity of complex **1** toward HBpin (**3**) under relevant reaction conditions was investigated in order to get a first insight into its mode of action in these challenging reductive processes. Reacting **1** with KO^*t*^Bu at room temperature in d_8_-THF lead to an immediate color change from yellow to dark violet (Fig. 2a). Spectroscopic data confirmed the formation of the unsaturated species **I** (^31^P{^1^H} NMR: *δ* *=* 61.26 ppm), in line with the known reactivity of other Mn complexes containing PNP-pincer ligands^47^. Addition of pinacolborane **3** to this solution at room temperature restored the yellow color while ^1^H and ^31^P{^1^H} NMR revealed new signals at −6.56 ppm (broad, half-width 88 Hz) and 65.21 ppm, respectively (Fig. 2b). This new species **IIa** converts slowly to the Mn-hydride complex **II**, as indicated by appearance of the typical resonances in the ^1^H NMR and ^31^P{^1^H} NMR spectrum at *δ* = −7.67 ppm (t, *J* = 32 Hz) and *δ =* 57.87 ppm, respectively. Heating the solution at 50 °C for 30 min lead to a ratio of **IIa/II** of 90:10 with nearly quantitative conversion to **II** after standing at room temperature for 2 days (see Supplementary Information for details).

Single crystals suitable for X-ray diffraction of the intermediately formed new complex **IIa** were obtained by the slow diffusion of hexane into a concentrated solution of dichloromethane (Fig. 2c). The most salient feature of this structure is the four-membered ring Mn–H–B–N that defines a crucial intermediate during the heterolytic B–H bond cleavage across the Mn-amido group. Intermediate **IIa** showed shorter distance between Manganese and nitrogen atom (Mn(1)-N(1) = 218.7 pm) in comparison with **1** (Mn(1)-N(4) = 227.4 pm). It is, however, longer than Mn = N double bonds in complexes with structures analogous to species **I** (for comparison see the Mn-PNP^*iPr*^ MACHO-complex: Mn–N, 212.3 pm, Mn=N, 189.0 ppm)^47^.

### Proposed mechanism

**Fig. 3:**
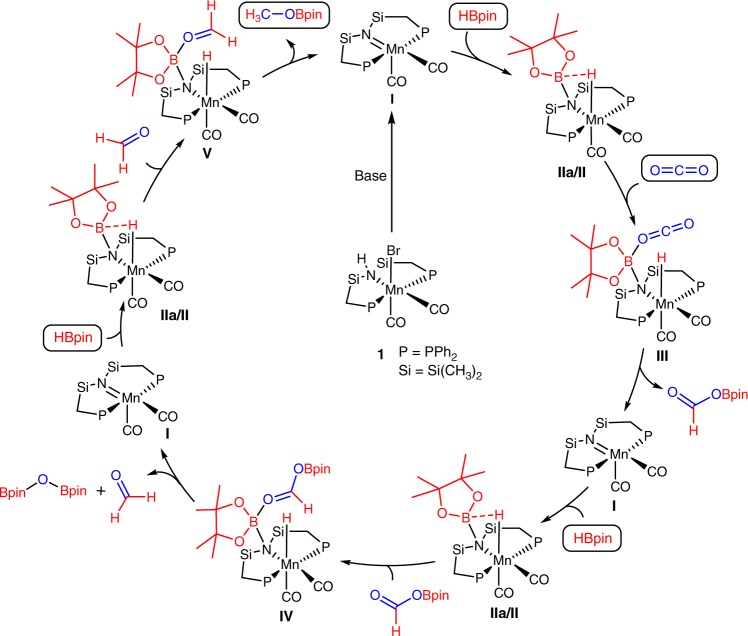
Suggested reaction mechanism for the hydroboration of CO_2_ catalyzed by complex **1**

On basis of the experimental observations and current literature precedent in Manganese catalysis, a tentative mechanism for the activation of the B–H bond and its transfer to the rather inert C=O units using complex **1** can be proposed as exemplified for the hydroboration of carbon dioxide in Fig. 3^48–52^. In the presence of base, complex **1** reacts to the actual active species **I**. Reaction with pinacolborane leads to complex **IIa** with activation of the B–H bond. This species may react with carbon dioxide directly or after B–H cleavage with hydride species **II**. Given the slow rate of the B–H bond cleavage, we currently favor the direct attack. The hydride complex **II** could carry an alternative catalytic cycle involving a second molecule of pinacolborane, similar to the mechanism deduced from kinetic studies most recently by Gade et al. for hydroboration of ketones and aldehydes with a Mn-catalyst that cannot form a Mn=N unit^48^. In case of pre-catalyst **1**, an additional open coordination site would have to be provided, e.g., through loss of CO, to enable such a cycle that would bypass the formation of species **I** and **IIa**. Although we have currently no evidence to suggest this alternative pathway for pre-catalyst **1**, the low energy barriers observed with the Gade catalyst indicate that this possibility should be considered in future mechanistic studies also. The carbonyl group can interact with the Lewis acidic boron center, leading to an activation for nucleophilic attack as indicated in intermediate **III**. The reduced formoxyborane HC(O)OBpin is liberated to regenerate the active complex **I**. Subsequently, pinacolborane reacts again with the active species **I** to give the intermediate **IIa**. Analogous reduction of the C=O unit of the formoxyborane leads to the acetal H_2_C(OBpin)_2_ at this stage, that cleaves into formaldehyde and BpinOBpin. The same steps can be once more repeated with the carbonyl of the formaldehyde unit until it reaches the alcohol/methanol stage. Carbonates can be converted into the corresponding alkyl boronate esters in full analogy, while carboxylic acids can enter the catalytic manifold in form of the boronate derivatives RC(O)OBpin that are spontaneously formed in presence of HBpin as verified experimentally.

## Discussion

In conclusion, the manganese pincer complex (**1**) revealed remarkable catalytic activity for the reduction of carboxylic acids, organic carbonates, and CO_2_ using pinacolborane as the reducing agent. The transformation can be applied for a remarkable broad substrate scope. These results significantly enlarge the potential of molecular catalysts based on earth abundant and non-toxic manganese as active metal for de- and re-functionalization of challenging substrates. The synthetic protocols and the molecular insight from this work may thus contribute to the development of benign catalytic systems for sustainable processes including the use of non-fossil carbon feedstocks derived from biomass or carbon dioxide.

## Methods

### General procedure for the Mn(I)-catalyzed hydroboration of carboxylic acids

A mixture of [Mn]-precatalyst (**1**) (0.2 mol%), potassium *tert*-butoxide (0.5 mol%), pinacolborane (3 eq.), corresponding carboxylic acid (1 eq.) and mesitylene were stirred at 115 °C for the indicated reaction times. Subsequently, a sample (15 µL) of the reaction mixture in CDCl_3_ (0.5 mL) was subjected to ^1^H-NMR spectroscopy to determine the yield in alkyl boronate ester.

### General procedure for the Mn(I)-catalyzed hydroboration of carbonates

Carbonate (**5**) (1 mmol) was added at room temperature to a mixture of [Mn]-precatalyst (**1**) (0.1 mol%), NaO^*t*^Bu (0.3 mol %) and pinacolborane (3 mmol). The reaction mixture was then heated at 90 °C for 8 h. Afterwards, the reaction medium was cooled down to room temperature, and mesitylene was added as an internal standard. Subsequently, a sample (10 µL) of the reaction mixture in CDCl_3_ (0.4 mL) was subjected to ^1^H-NMR spectroscopy to determine the yield in alkyl boronate ester.

### General procedure for the Mn(I)-catalyzed hydroboration of carbon dioxide

Carbon dioxide was purged three times through a schlenk containing a mixture of [Mn]-precatalyst (**1**) (0.73 mg, 0.036 mol%), NaO^*t*^Bu (0.288 mg, 0.1 mol %) and pinacolborane (353.2 mg, 2.76 mmol) in the indicated solvent. Afterwards, the schlenk tube was equipped with a CO_2_ balloon and the reaction mixture was heated at the indicated temperatures. The reaction medium was then cooled down to room temperature, and mesitylene was added as an internal standard. Subsequently, a sample (10 µL) of the reaction mixture in CDCl_3_ (0.4 mL) was subjected to ^1^H-NMR spectroscopy to determine the yield in alkyl boronate ester.

## Electronic supplementary material

Supplementary Information

## Data Availability

The X-ray crystallographic data for the structure of complex (**1**) and (**IIa**) have been deposited at the Cambridge Crystallographic Data Centre (CCDC) under the deposition numbers CCDC 1868572 and 1868571, respectively. These data can be obtained free of charge via www.ccdc.cam.ac.uk/data_request/cif. The authors declare that all data supporting the findings of this study are available within the manuscript and its Supplementary Information files. All other data are available from the authors upon reasonable request.
