# Supplementary material for: Manganese-catalyzed hydroboration of carbon dioxide and other challenging carbonyl groups
Source: Nat Commun. 2018 Oct 30;9:4521. doi: 10.1038/s41467-018-06831-9 (PMC6207666; doi:10.1038/s41467-018-06831-9)
Supplement: Supplementary file 1 — Supplementary Information [file 41467_2018_6831_MOESM1_ESM.pdf]

Supporting Information

Supplementary Methods

General considerations

All reactions involving air sensitive compounds were carried out under Argon in flame-dried glassware, ensuring rigorously inert conditions. The solvents were purified using solvent purification-systems and were stored and handled under Argon. Chemicals were purchased from Sigma-Aldrich, Alfa-Aesar, abcr, Acros Organics, TCI chemicals and used without further purification. NMR-spectra were recorded on Bruker AV-300, AV-400, DPX-300 spectrometers at the indicated temperatures with the chemical shifts (δ) given in ppm relative to TMS and the coupling constants (J) in Hz. The solvent signals were used as references and the chemical shifts converted to the TMS scale (Acetonitrile-d_3: δ_H = 1.94 ppm and δ_C = 118.3, 1.3 ppm; $CDCl_3$: δ_H = 7.26 ppm and δ_C = 77.1 ppm; C_6D_6: δ_H = 7.16 ppm and δ_C = 128.1 ppm; THF-d_8: δ_H = 1.72 ppm, 3.58 ppm and δ_C = 67.2, 25.3 ppm; Toluene-d_8: δ_H = 2.08, 6.97, 7.01, 7.09 ppm and δ_C = 137.5, 128.9, 127.9, 125.1, 20.4 ppm DMSO-d_6: δ_H = 2.50 ppm and δ_C = 39.5 ppm).[1] HRMS spectra were recorded on an Bruker ESQ3000 spectrometer.

Synthesis of the ligand (Si-PNP)

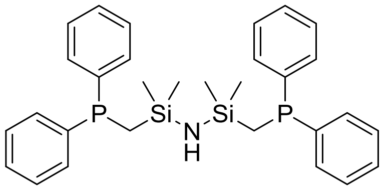

1,3-bis((diphenyl-phosphino)methyl)tetramethyldi-silazane. The organic ligand **(Si-PNP)** was prepared by following a modified literature procedure.[2] *n*-BuLi (2.5M in hexane, 6.65 mmol, 2.7 mL) was added dropwise to a solution of Ph_2PH (1.24 g, 6.65 mmol) in THF (10 mL) at -78 °C, leading to a bright yellow solution. The resulting mixture was stirred at -78 °C for 30 min and then slowly warmed up to room temperature where it was stirred for another 30 min to ensure full conversion of the starting material. Subsequently, the mixture was cooled down to - 78 °C and added *via* cannula transfer to a pre-cooled solution (-78 ^{o}C) of 1,3-Bis(chloromethyl)-1,1,3,3-tetramethyldisilazane (0.76 g, 3.33 mmol) in THF (8 mL) leading to a color change from bright yellow to light pale. After warming up to room temperature, the resulting solution was stirred for another 12 h. The volatiles were then removed in vacuo. The residue was extracted with pentane (15 mL) and filtered over fritted glass to remove undissolved LiCl. Removals of the solvent in vacuo lead to the targeted ligand **Si-PNP** as a colourless oil (1.3 g, 2.45 mmol, 74%) which was used without further purification. **^{1}H-NMR** (300 MHz, C_6D_6, 298 K): δ = 7.52-7.47 (m, 8H), 7.09-7.04 (m, 12H), 1.30 (s, 4H), 0.06 (s, 12H); **^{31}P{^{1}H}-NMR** (300 MHz, C_6D_6, 298 K): δ = -22.51.

Synthesis of manganese complex (1)

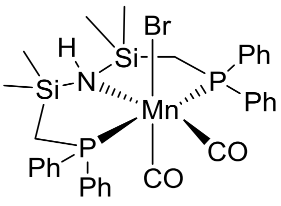

1

$Mn(Ph_2PCH_2SiMe_2)_2NH(CO)_2Br$ (1). A solution of **Si-PNP** (0.210 g, 0.396 mmol) in toluene (1 mL) was added to a suspension of $Mn(CO)_5Br$ (0.109 g, 0.395 mmol) in toluene (4 mL). The reaction mixture was stirred at 100 °C for 12 h. The volatiles were removed in vacuo and the residue was washed with hexane (2 × 5 mL). Upon drying in vacuo, the residue solidified and complex **1** was obtained as a bright yellow powder (0.265 g, 0.367 mmol, 93%). Bright yellow crystals suitable for X-ray diffraction were grown by layering a solution of complex **1** (20 mg, 0.0028 mmol) in dichloromethane (0.5 mL) with hexane (5 mL). **^{1}H-NMR** (300 MHz, THF-d_8, 298 K): δ = 7.88-7.79 (m, 8H), 7.34-7.22 (m, 12H), 1.92 (s, 4H), 0.41 (s, 6H), 0.13 (s, 6H); **^{31}P{^{1}H}-NMR** (23 MHz, THF-d_8, 298 K): δ = 48.46; **^{13}C{^{1}H}-NMR** (100.1 MHz, CD_2Cl_2, 298 K): δ = 138.8, 138.6, 137.7, 137.5, 137.4, 132.7, 132.6, 132.6, 131.6, 129.5, 128.8, 128.3, 14.7, 3.6, 0.7; **HRMS (ESI$^+$):** *m/z*: calcd. for $C_{32}H_{37}BrMnNP_2Si_2$ $[M]^+$: 719.03964; found: 719.03961.

Optimization of the reaction conditions for the hydroboration of acetic acid

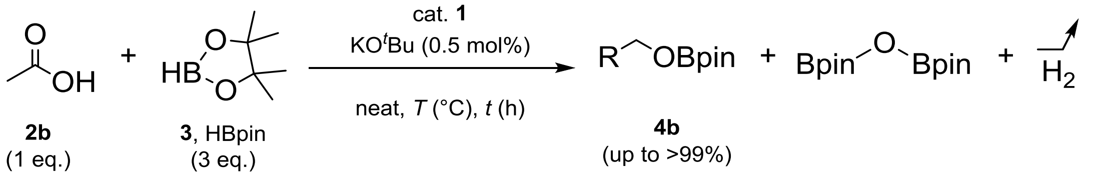

Supplementary Table 1: Optimization studies for the hydroboration of acetic acid in absence of solvent.

Entry	Catalyst (mol%)	Base	T (°C)	T (h)	Yield (%)^a
1a	0.1	KO^{t}Bu	115	5	63
1b	0.1	KO^{t}Bu	115	17	77
1c	0.1	KO^{t}Bu	115	20	84
1d	0.1	KO^{t}Bu	115	24	>99
2a	0.2	KO^{t}Bu	115	5	69
2b	0.2	KO^{t}Bu	115	17	88
2c	0.2	KO^{t}Bu	115	20	>99
2d	0.2	KO^{t}Bu	115	24	>99
3a	0.5	KO^{t}Bu	115	5	69
3b	0.5	KO^{t}Bu	115	17	91

3c	0.5	KO^{t}Bu	115	20	>99
3d	0.5	KO^{t}Bu	115	24	>99
4a	1.0	KO^{t}Bu	115	5	88
4b	1.0	KO^{t}Bu	115	17	>99
4c	1.0	KO^{t}Bu	115	20	>99
4d	1.0	KO^{t}Bu	115	24	>99
5	No cat.	KO^{t}Bu	115	24	30
6	0.2	No base	115	24	15
7a	0.2	NEt_3	115	5	78
7b	0.2	NEt_3	115	17	84
7c	0.2	NEt_3	115	20	88
7d	0.2	NEt_3	115	24	90
8	0.2	NaOH	115	24	39

[a]Yields were determined by ^{1}H-NMR analysis, by using mesitylene as internal standard.

Supplementary Table 2: Optimization studies for the hydroboration of acetic acid in solution.[a]

Entry	Solvent	Base	T (h)	Yield (%)[b]
2a	No	KO^{t}Bu	5	69
9	Acetonitrile-d_3	KO^{t}Bu	5	14
10	Benzene-d_6	KO^{t}Bu	5	10
11	Chloroform-d	KO^{t}Bu	5	16
12	Tetrahydrofuran-d_8	KO^{t}Bu	5	8

[a]Conditions: **1** (0.2 mol%), KO^{t}Bu (0.5mol%), **2b** (1 eq.) and **3** (3 eq.) in 1mL of solvent at 115 °C for 5 h. [b]Yields were determined by ^{1}H NMR analysis, by using mesitylene as internal standard.

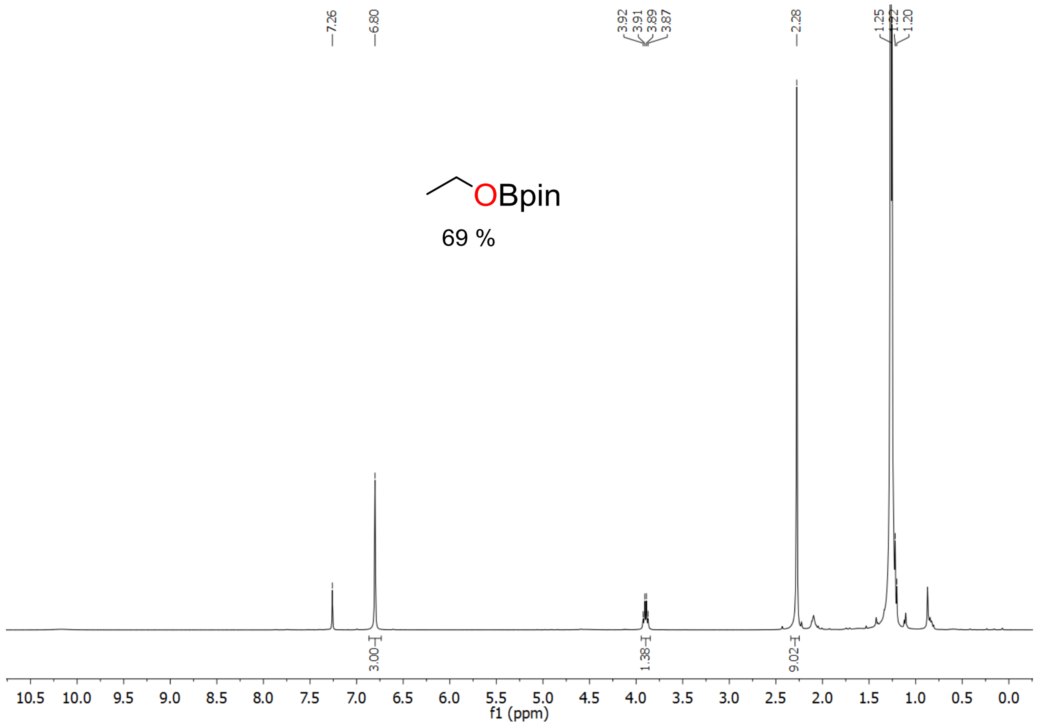

Supplementary Figure 1: ^{1}H NMR (400 MHz, $CDCl_3$, 296 K) Hydroboration of acetic acid (Supplementary Table 1 and Supplementary Table 2, entry 2a) using mesitylene (1 eq.) as internal standard.

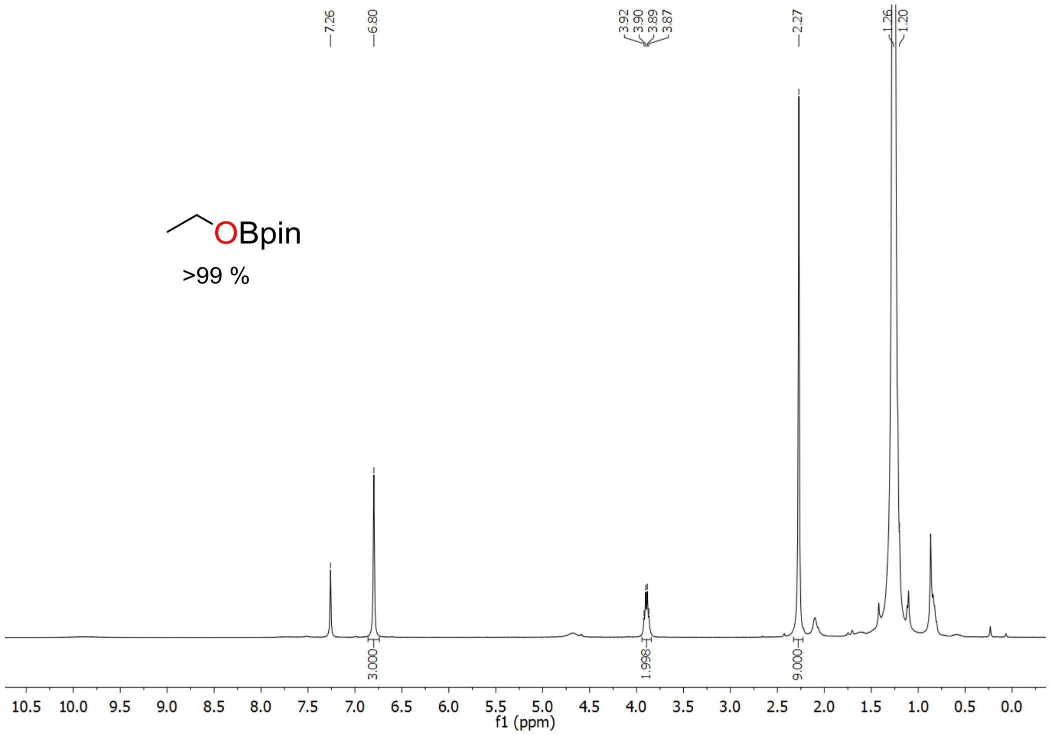

Supplementary Figure 2: ^{1}H NMR (400 MHz, $CDCl_3$, 296 K) Hydroboration of acetic acid (Supplementary Table 1, entry 2c) using mesitylene (1 eq.) as internal standard.

General Procedure for the hydroboration of carboxylic acids

A mixture of manganese catalyst **1** (0.2 mol%), potassium *tert*-butoxide (0.5 mol%), pinacolborane (3 eq.), corresponding carboxylic acid (1 eq.) and mesitylene were stirred at 115 °C for the indicated reaction times. Subsequently, a sample (15 μL) of the reaction mixture in $CDCl_3$ (0.5 mL) was subjected to ^{1}H-NMR spectroscopy to determine the yield in alkyl boronate ester.

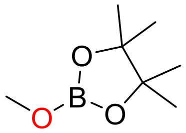

2-methoxy-4,4,5,5-tetramethyl-1,3,2-dioxaborolane (4a). Prepared by following the general experimental procedure with: **1** (0.5 mg, 0.2 mol%), **2a** (14.3 mg, 0.33 mmol), **3** (127.98 mg, 1 mmol) and KO^{t}Bu (0.2 mg, 0.5 mol%) for 20 h. **^{1}H-NMR** (400 MHz, $CDCl_3$, 296 K): δ = 3.60 (s, 3H), 1.25 (s-overlap, 36H); **^{13}C APT-NMR** (101 MHz, $CDCl_3$, 296 K): δ = 83.3, 52.8, 24.8. The obtained analytical data are consistent with those previously reported in the literature.[3]

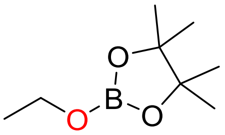

2-ethoxy-4,4,5,5-tetramethyl-1,3,2-dioxaborolane (4b). Prepared by following the general experimental procedure with: **1** (0.5 mg, 0.2 mol%), **2** (20.0 mg, 0.33 mmol), **3** (127.98 mg, 1 mmol) and KO^{t}Bu (0.2 mg, 0.5 mol%) for 20 h. **^{1}H-NMR** (400 MHz, $CDCl_3$, 296 K): δ = 3.83 (q, J = 7.0 Hz, 2H), 1.20 (s-overlap, 39H); **^{13}C APT-NMR** (101 MHz, $CDCl_3$, 296 K): δ = 83.5, 61.1, 25.0, 17.7. The analytical data are consistent with those previously reported in the literature. [4,5]

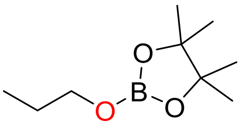

4,4,5,5-tetramethyl-2-propoxy-1,3,2-dioxaborolane (4c). Prepared by following the general experimental procedure with: **1** (0.5 mg, 0.2 mol%), **2** (24.7 mg, 0.33 mmol), **3** (127.98 mg, 1 mmol) and KO^{t}Bu (0.2 mg, 0.5 mol%) for 20 h. **^{1}H-NMR** (400 MHz, $CDCl_3$, 296 K): δ = 3.81 (t, J = 6.6 Hz, 2H), 1.60 (h, J = 7.1 Hz, 2H), 1.25 (s-overlap, 36H), 0.92 (t, J = 7.4 Hz, 3H); **^{13}C APT-NMR** (101 MHz, $CDCl_3$, 296 K): δ = 83.4, 67.0, 24.9, 10.5. The analytical are consistent with those previously reported in the literature.[6]

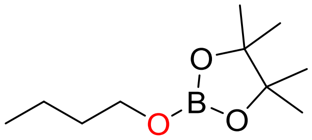

2-butoxy-4,4,5,5-tetramethyl-1,3,2-dioxaborolane (4d). Prepared by following the general experimental procedure with: **1** (0.5 mg, 0.2 mol%), **2** (29.4 mg, 0.33 mmol), **3** (127.98 mg, 1 mmol) and KO^{t}Bu (0.2 mg, 0.5 mol%) for 20 h. **^{1}H-NMR** (400 MHz, $CDCl_3$, 296 K): δ = 4.07 (t, J = 6.6 Hz, 2H), 1.65 (tt, J = 10.1, 4.9 Hz, 2H), 1.38 (dq, J = 14.9, 7.6, 7.0 Hz, 2H), 1.25 (s-overlap, 36H), 0.92 (t, J = 7.2 Hz, 3H); **^{13}C APT-NMR** (101 MHz, $CDCl_3$, 296 K): δ = 83.5, 64.5, 36.7, 24.9, 19.6, 14.0. The analytical data are consistent with those previously reported in the literature.[7]

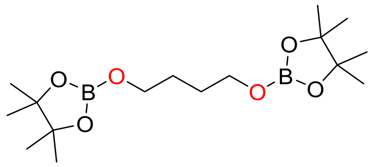

1,4-bis((4,4,5,5-tetramethyl-1,3,2-dioxaborolan-2-yl)oxy)butane (4e). Prepared by following the general experimental procedure with: **1** (0.5 mg, 0.2 mol%), **2** (39.4 mg, 0.33 mmol), **3** (255.96 mg, 2 mmol) and KO^{t}Bu (0.2 mg, 0.5 mol%) for 36 h. **^{1}H-NMR** (400 MHz, $CDCl_3$, 296 K): δ = 3.83 (br, 4H), 1.24 (s-overlap, 76 H); **^{13}C{^{1}H}-NMR** (101 MHz, $CDCl_3$, 296 K): δ = 83.3, 64.8, 27.8, 24.8.

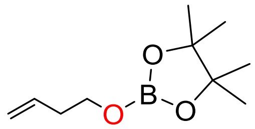

2-(but-3-en-1-yloxy)-4,4,5,5-tetramethyl-1,3,2-dioxaborolane (4f). Prepared by following the general experimental procedure with: **1** (0.5 mg, 0.2 mol%), **2** (28.7 mg, 0.33 mmol), **3** (127.98 mg, 1 mmol) and KO^{t}Bu (0.2 mg, 0.5 mol) for 20 h. **^{1}H-NMR** (400 MHz, $CDCl_3$, 296 K): δ = 5.79 (ddt, J = 13.7, 7.0, 3.2 Hz, 1H), 5.12-5.03 (m, 2H), 3.90 (t, J = 6.7 Hz, 2H), 2.73 (m, 2H), 1.25 (s-overlap, 36H); **^{13}C APT-NMR** (101 MHz, $CDCl_3$, 296 K): δ = 137.7, 126.9, 82.6, 67.6, 33.4, 24.7. The analytical data of known products are consistent with those previously reported in the literature.[8]

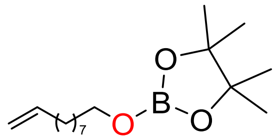

2-(dec-9-en-1-yloxy)-4,4,5,5-tetramethyl-1,3,2-dioxaborolane (4g). Prepared by following the general experimental procedure with: **1** (0.5 mg, 0.2 mol%), **2** (56.8 mg, 0.33 mmol), **3** (127.98 mg, 1 mmol) and KO^{t}Bu (0.2 mg, 0.5 mol%) for 24 h. **^{1}H-NMR** (400 MHz, $CDCl_3$, 296 K): δ = 5.79 (td, J = 16.9, 6.7 Hz, 1H), 4.94 (dd, J = 25.0, 13.6 Hz, 2H), 3.81 (t, J = 6.2 Hz, 2H), 2.01 (q, J = 6.4 Hz, 2H), 1.73-1.68 (m, 4H), 1.25 (s-overlap, 44H); **^{13}C{^{1}H}-NMR** (101 MHz, $CDCl_3$, 296 K): δ = 139.4, 114.4, 83.4, 65.3, 34.1, 32.7, 29.9, 29.6, 29.3, 25.8, 24.8.

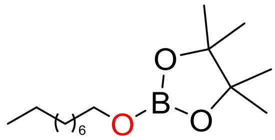

4,4,5,5-tetramethyl-2-(nonyloxy)-1,3,2-dioxaborolane (4h). Prepared by following the general experimental procedure with: **1** (0.5 mg, 0.2 mol%), **2** (52.7 mg, 0.33 mmol), **3** (127.98 mg, 1 mmol) and KO^{t}Bu (0.2 mg, 0.5 mol%) for 20 h. **^{1}H-NMR** (400 MHz, $CDCl_3$, 296 K): δ = 3.86 (t, J = 6.5 Hz, 2H), 1.62-1.55 (m, 2H), 1.44-1.30 (m, 2H), 1.25 (s-overlap, 46H), 0.93 (t, J = 6.6 Hz, 3H); **^{13}C APT-NMR** (101 MHz, $CDCl_3$, 296 K): δ = 83.2, 65.2, 32.3, 31.9, 30.0, 29.7, 26.0, 24.8, 23.1, 14.4. The analytical data of known products are consistent with those previously reported in the literature.[9]

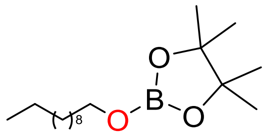

4,4,5,5-tetramethyl-2-(undecyloxy)-1,3,2-dioxaborolane (4i). Prepared by following the general experimental procedure with: **1** (0.5 mg, 0.2 mol%), **2** (62.1 mg, 0.33 mmol), **3** (127.98 mg, 1 mmol) and KO^{t}Bu (0.2 mg, 0.5 mol%)

for 20 h. ^{1}**H-NMR** (400 MHz, $CDCl_3$, 296 K): δ = 3.82 (t, 2H, J = 6.3 Hz, 2H), 1.60-1.49 (m, 3H), 1.25 (s-overlap, 50H), 0.86 (t, J = 5.8 Hz, 3H); ^{13}**C{^{1}H}-NMR** (101 MHz, $CDCl_3$, 296 K): δ = 83.4, 65.3, 32.2, 31.7, 29.9, 29.6, 25.9, 24.8, 23.0, 14.4.

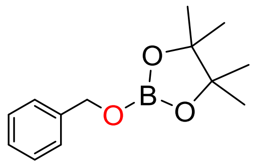

2-(benzyloxy)-4,4,5,5-tetramethyl-1,3,2-dioxaborolane (4j). Prepared by following the general experimental procedure with: **1** (0.5 mg, 0.2 mol%), **2** (40.7 mg, 0.33 mmol), **3** (127.98 mg, 1 mmol) and KO^{t}Bu (0.2 mg, 0.5 mol%) for 24 h. ^{1}**H-NMR** (400 MHz, $CDCl_3$, 296 K): δ = 7.35-7.22 (m, 5H), 4.92 (s, 2H), 1.25 (s-overlap, 36H); ^{13}**C APT-NMR** (101 MHz, $CDCl_3$, 296 K): δ = 138.0, 127.8, 127.4, 127.1, 83.4, 67.1, 25.0. The analytical data of known products are consistent with those previously reported in the literature.[8,10]

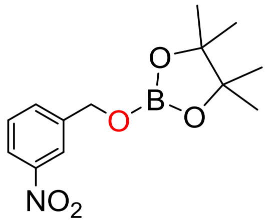

4,4,5,5-tetramethyl-2-((3-nitrobenzyl)oxy)-1,3,2-dioxaborolane (4k). Prepared by following the general experimental procedure with: **1** (0.5 mg, 0.2 mol%), **2** (55.7 mg, 0.33 mmol), **3** (127.98 mg, 1 mmol) and KO^{t}Bu (0.2 mg, 0.5 mol%) for 24 h. ^{1}**H-NMR** (400 MHz, $CDCl_3$, 296 K): δ = 8.22 (d, J = 2.0 Hz, 1H), 8.12 (d, J = 8.2 Hz, 1H), 7.66 (d, J = 7.6 Hz, 1H), 7.50 (t, J = 7.9 Hz, 1H), 5.00 (s, 2H), 1.25 (s, 36H); ^{13}**C{^{1}H}-NMR** (101 MHz, $CDCl_3$, 296 K): δ = 148.6, 141.6, 132.9, 129.5, 122.7, 121.9, 83.4, 65.8, 24.8.

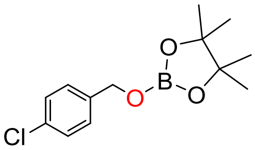

2-((4-chlorobenzyl)oxy)-4,4,5,5-tetramethyl-1,3,2-dioxaborolane (4l). Prepared by following the general experimental procedure with: **1** (0.5 mg, 0.2 mol%), **2** (52.2 mg, 0.33 mmol), **3** (127.98 mg, 1 mmol) and KO^{t}Bu (0.2 mg, 0.5 mol%) for 20 h. ^{1}**H-NMR** (400 MHz, $CDCl_3$, 296 K): δ = 7.41-7.19 (m, 4H), 4.88 (s, 2H), 1.26 (s-overlap, 36H); ^{13}**C{^{1}H}-NMR** (101 MHz, $CDCl_3$, 296 K): δ = 138.0, 137.5, 128.5, 128.2, 82.9, 65.9. The analytical data of known products are consistent with those previously reported in the literature.[11]

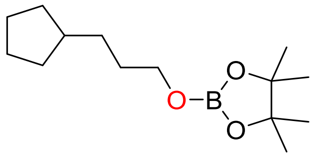

2-(3-cyclopentylpropoxy)-4,4,5,5-tetramethyl-1,3,2-dioxaborolane (4m). Prepared by following the general experimental procedure with: **1** (0.5 mg, 0.2 mol%), **2** (47.4 mg, 0.33 mmol), **3** (127.98 mg, 1 mmol) and KO^{t}Bu (0.2 mg, 0.5 mol%) for 24 h. ^{1}**H-NMR** (400 MHz, $CDCl_3$, 296 K): δ = 3.82 (t, J = 6.6 Hz, 2H), 1.75 (tt, J = 8.7, 4.5 Hz, 2H), 1.65-1.43 Hz (m, 9H), 1.26 (s, 36H), 1.11-1.06 (m, 2H); ^{13}**C{^{1}H}-NMR** (101 MHz, $CDCl_3$, 296 K): δ = 83.4, 65.5, 40.1, 32.9, 32.3, 31.0, 25.5, 24.8.

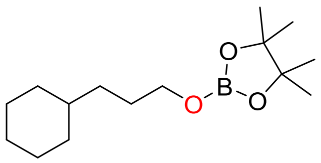

2-(3-cyclohexylpropoxy)-4,4,5,5-tetramethyl-1,3,2-dioxaborolane (4n). Prepared by following the general experimental procedure with: **1** (0.5 mg, 0.2 mol%), **2** (52.1 mg, 0.33 mmol), **3** (127.98 mg, 1 mmol) and KO^{t}Bu (0.2 mg, 0.5 mol%) for 24 h. ^{1}**H-NMR** (400 MHz, $CDCl_3$, 296 K): δ = 3.80 (t, J = 6.71 Hz, 2H), 1.68-1.49 (m, 15H), 1.25 (s-overlap, 38H); ^{13}**C{^{1}H}-NMR** (101 MHz, $CDCl_3$, 296 K): δ = 83.4, 65.6, 37.6, 33.6, 33.2, 29.0, 26.6, 24.8.

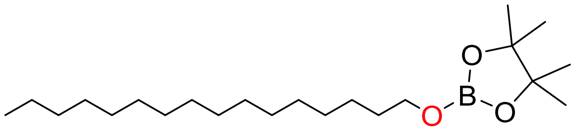

2-(hexadecyloxy)-4,4,5,5-tetramethyl-1,3,2-dioxaborolane (4o). Prepared by following the general experimental procedure with: **1** (0.5 mg, 0.2 mol%), **2** (85.7 mg, 0.33 mmol), **3** (127.98 mg, 1 mmol) and KO^{t}Bu (0.2 mg, 0.5 mol%) for 24 h. ^{1}**H-NMR** (400 MHz, $CDCl_3$, 296 K): δ = 3.81 (t, J = 6.6 Hz, 2H), 1.61-1.38 (m, 2H), 1.25 (s, 47H), 0.86 (t, J = 6.7 Hz, 3H); ^{13}**C{^{1}H}-NMR** (101 MHz, $CDCl_3$, 296 K): δ = 83.6, 65.5, 32.5, 32.0, 30.0, 29.9, 26.2, 25.1, 23.3, 14.7.

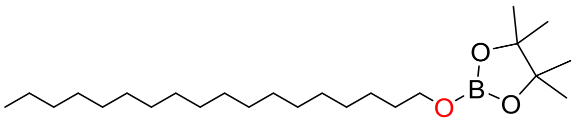

4,4,5,5-tetramethyl-2-(octadecyloxy)-1,3,2-dioxaborolane (4p). Prepared by following the general experimental procedure with: **1** (0.5 mg, 0.2 mol%), **2** (82.8 mg, 0.33 mmol), **3** (127.98 mg, 1 mmol) and KO^{t}Bu (0.2 mg, 0.5 mol%) for 24 h. ^{1}**H-NMR** (400 MHz, $CDCl_3$, 296 K): δ = 3.79 (t, J = 6.6 Hz, 2H), 1.54-1.49 (m, 4H), 1.24 (s-overlap, 64H), 0.84 (t, J = 6.6 Hz, 3H); ^{13}**C{^{1}H}-NMR** (101 MHz, $CDCl_3$, 296 K): δ = 83.3, 65.2, 32.2, 31.7, 29.9, 29.6, 25.8, 24.8, 22.9, 14.3.

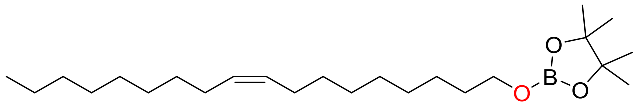

(Z)-4,4,5,5-tetramethyl-2-(octadec-9-en-1-yloxy)-1,3,2-dioxaborolane (4q). Prepared by following the general experimental procedure with: **1** (0.5 mg, 0.2 mol%), **2** (94.1 mg, 0.33 mmol), **3** (127.98 mg, 1 mmol) and KO^{t}Bu (0.2 mg, 0.5 mol%) for 24 h. ^{1}**H-NMR** (400 MHz, $CDCl_3$, 296 K): δ = 5.37-5.29 (m, 2H), 3.82 (t, J = 6.6 Hz, 2H), 2.01-1.97 (m, 4H), 1.58-1.51 (m, 2H), 1.26 (s-overlap, 58H), 0.87 (t, J = 6.8 Hz, 3H); ^{13}**C{^{1}H}-NMR** (101 MHz, $CDCl_3$, 296 K): δ = 130.2, 83.4, 65.3, 32.2, 31.7, 30.1, 29.8, 29.6, 27.5, 24.9, 23.0, 14.4.

Optimization of the reaction conditions for the hydroboration of ethylene carbonate

Ethylene carbonate (0.088 g, 1 mmol) was added at room temperature to a mixture of [Mn]-precatalyst **1**, NaO^{t}Bu and pinacolborane (0.435 mL, 3 mmol). The reaction mixture was then heated at the indicated temperature (Supplementary Table 3). After reaction time was reached and the reaction medium cooled down to room temperature, mesitylene was added as an internal standard. Subsequently, a sample (10 μL) of the reaction mixture in $CDCl_3$ (0.4 mL) was subjected to ^{1}H-NMR spectroscopy to determine the yield in alkyl boronate ester.

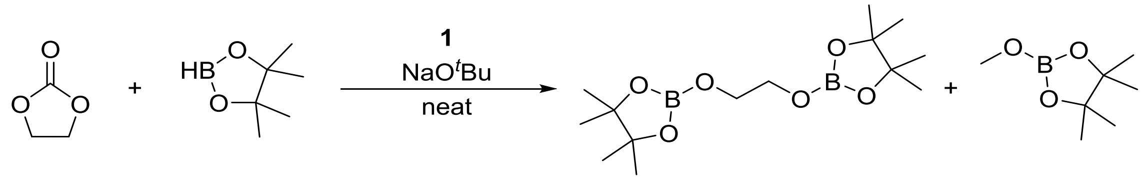

5a **3** **6a** **4a**

Supplementary Table 3: Optimization studies for the hydroboration of ethylene carbonate.

Entry	Catalyst (mol%)	NaO^{t}Bu (mol%)	Temp. (°C)	Time (h)	Yield (%)^a
1	0.25	0.4	90	4	97
2	0.1	0.3	90	4	77
3	0.1	0.3	100	4	88
4	0.1	0.3	90	8	95
5	-	-	110	12	12
6	-	0.3	110	12	13

^{a} Yields were determined by ^{1}H NMR spectroscopy using mesitylene as an internal standard.

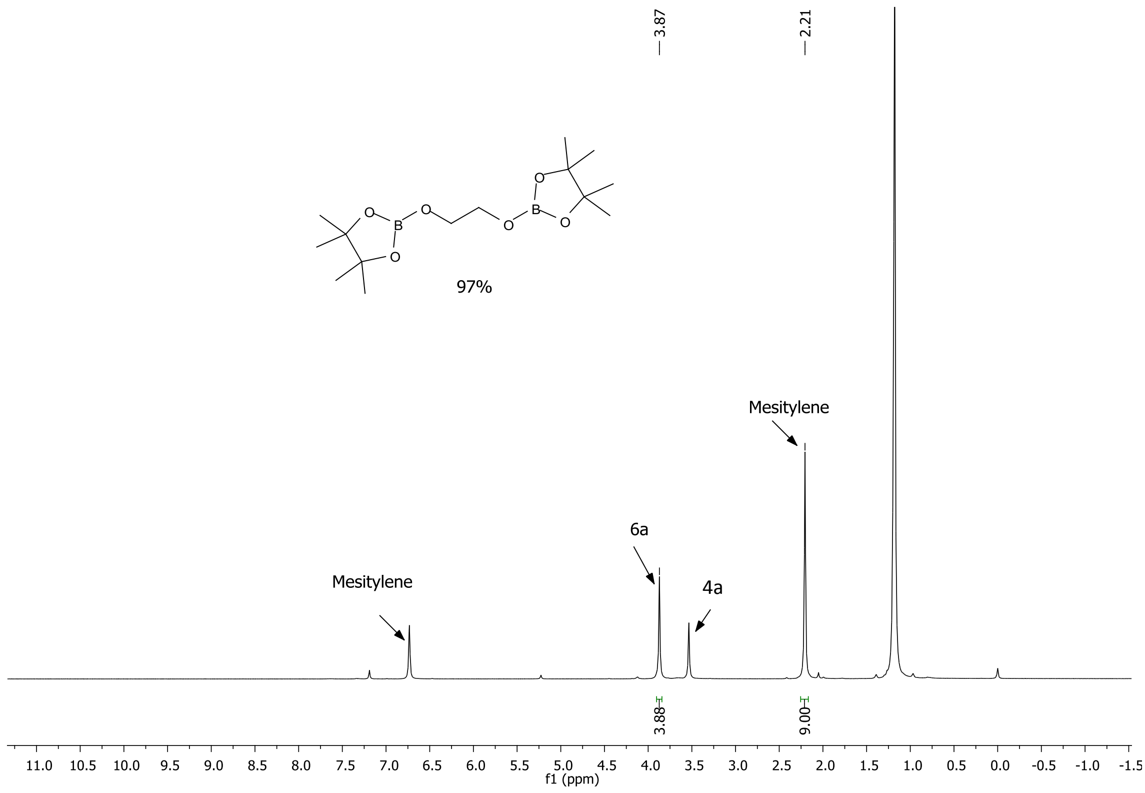

Supplementary Figure 3: ^{1}H-NMR (300 MHz, $CDCl_3$, 298 K) spectrum for the hydroboration of ethylene carbonate (Supplementary Table 3, entry 1).

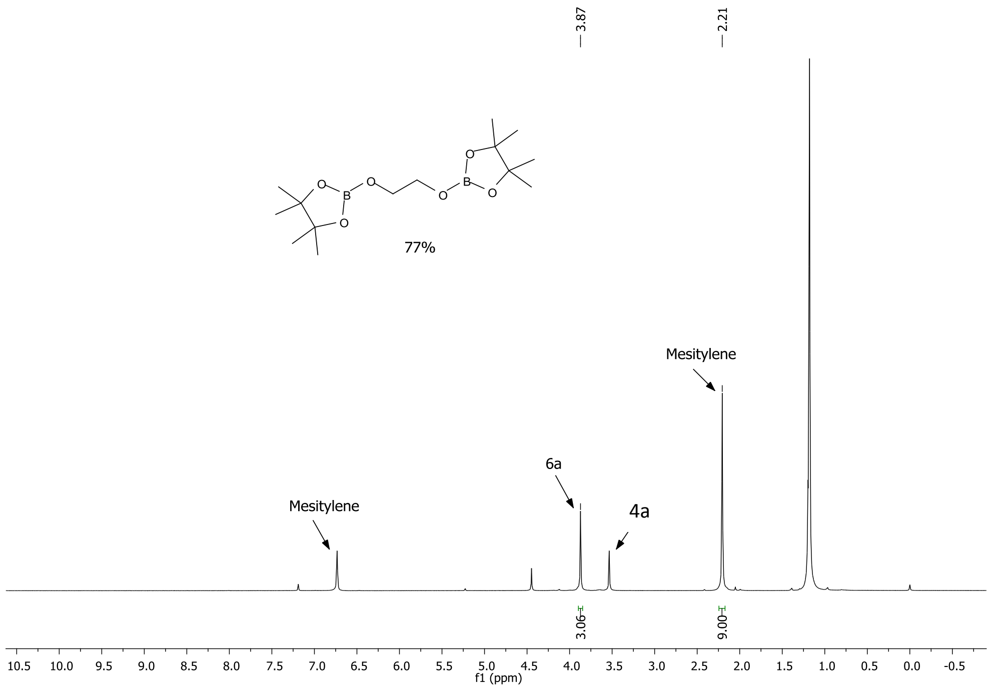

Supplementary Figure 4: ^{1}H-NMR (300 MHz, $CDCl_3$, 298 K) spectrum for the hydroboration of ethylene carbonate (Supplementary Table 3, entry 4).

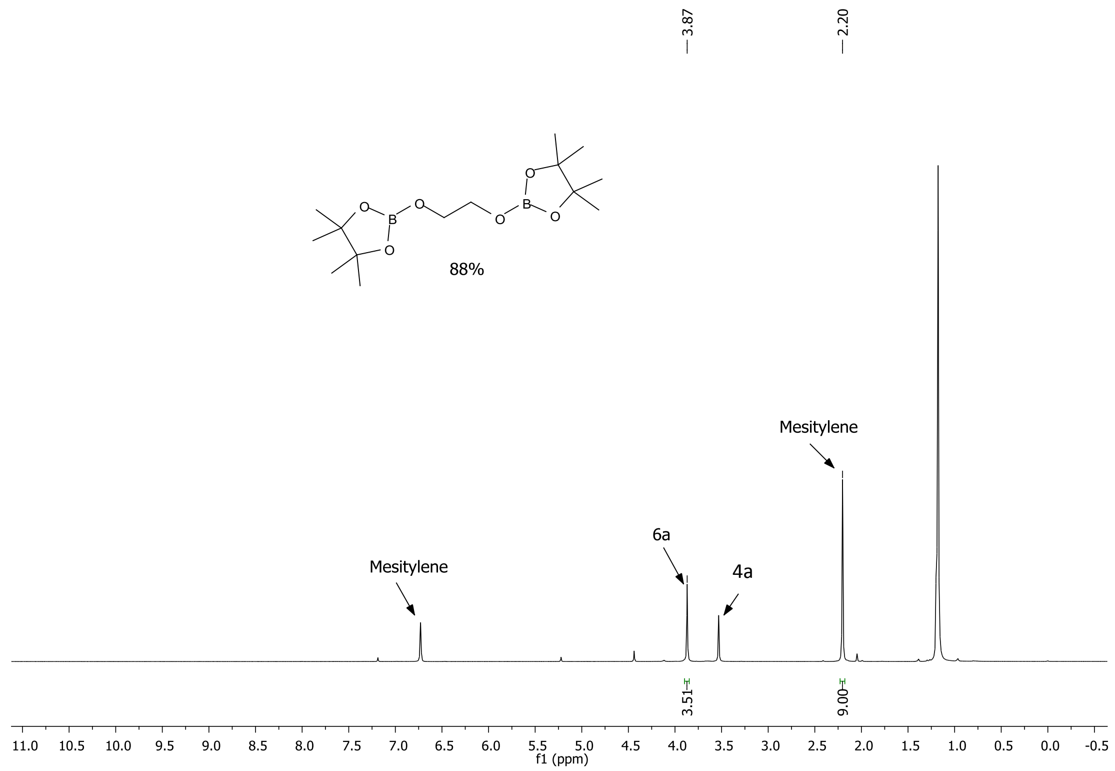

Supplementary Figure 5: ^{1}H-NMR (300 MHz, $CDCl_3$, 298 K) spectrum for the hydroboration of ethylene carbonate (Supplementary Table 3, entry 3).

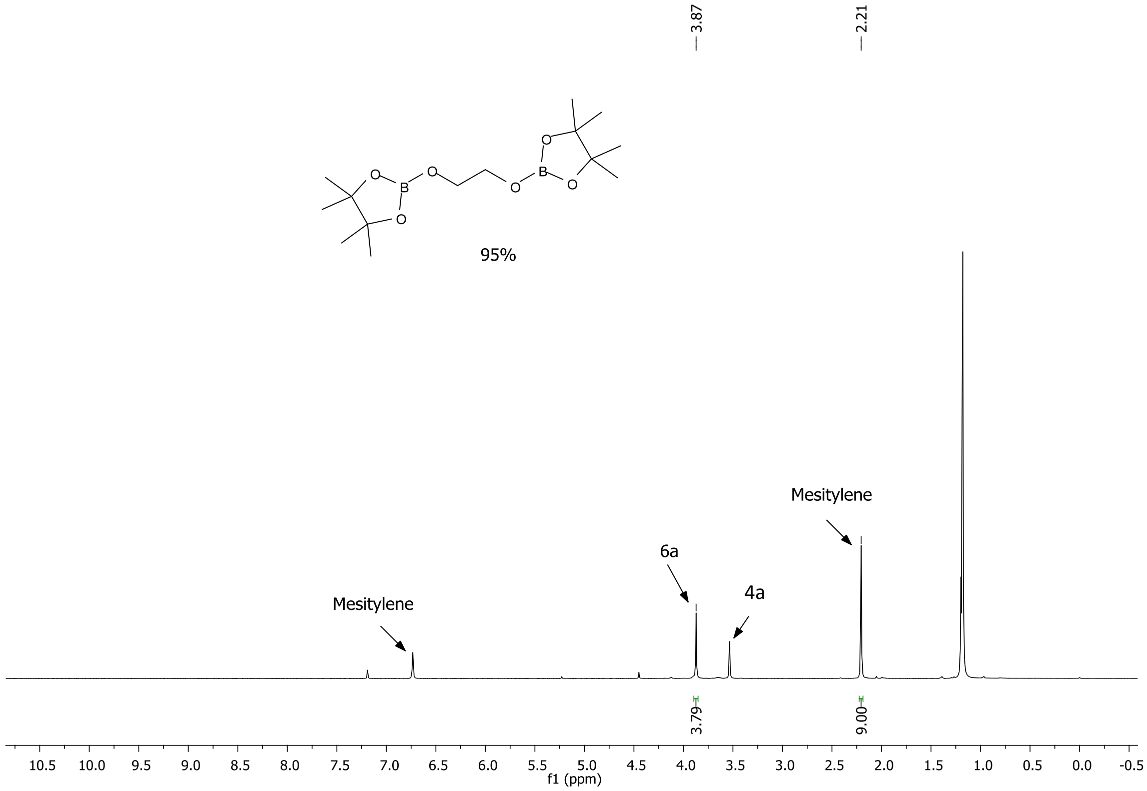

Supplementary Figure 6: ^{1}H-NMR (300 MHz, $CDCl_3$, 298 K) spectrum for the hydroboration of ethylene carbonate (Supplementary Table Supplementary Table 3, entry 3 and Table 2, entry a).

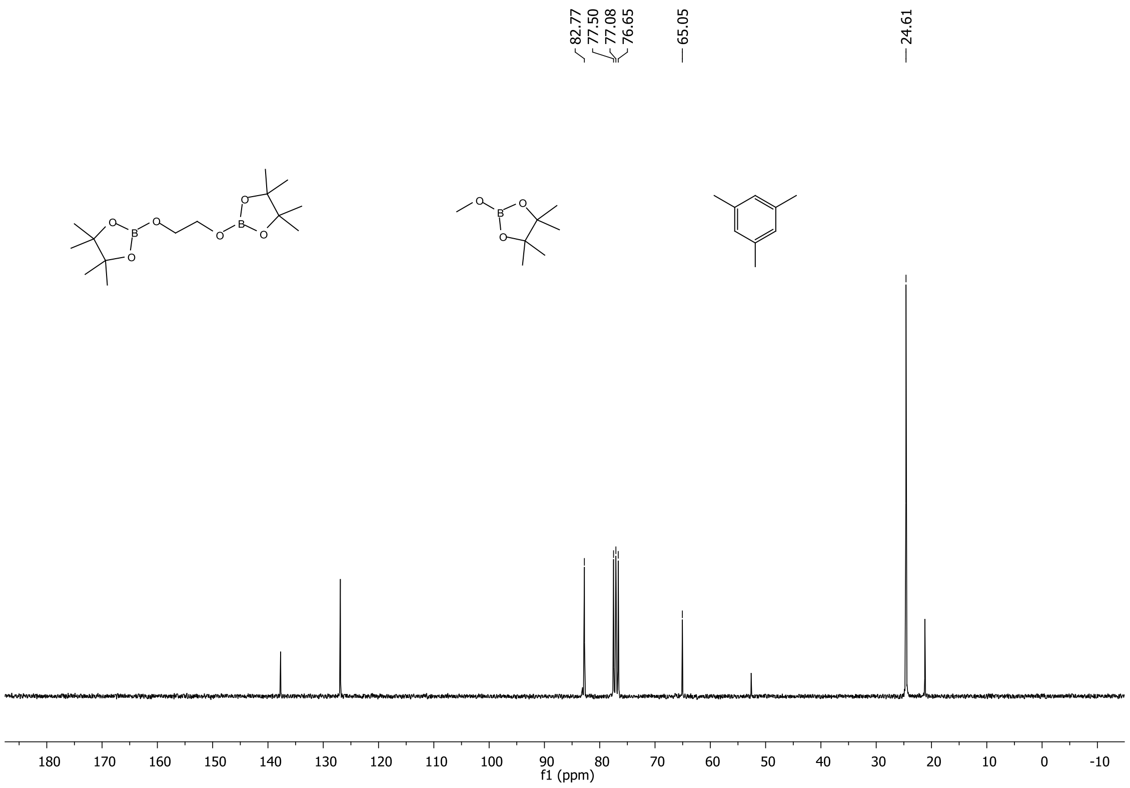

Supplementary Figure 7: $^{13}C\{^1H\}$-NMR (75 MHz, $CDCl_3$, 298 K) spectrum for the hydroboration of ethylene carbonate (Supplementary Table 3, entry 3 and Table 2, entry a).

General Procedure for the hydroboration of carbonates

Carbonate **5** (1 mmol) was added at room temperature to a mixture of [Mn]-precatalyst **1** (0.1 mol%), NaO^{t}Bu (0.3 mol %) and pinacolborane (3 mmol). The reaction mixture was then heated at 90 ^{o}C for 8 h. Afterwards, the reaction medium was cooled down to room temperature, and mesitylene was added as an internal standard. Subsequently, a sample (10 μL) of the reaction mixture in $CDCl_3$ (0.4 mL) was subjected to ^{1}H-NMR spectroscopy to determine the yield in alkyl boronate ester.

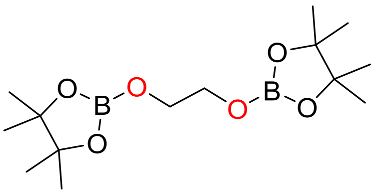

1,2-bis((4,4,5,5-tetramethyl-1,3,2-dioxaborolan-2-yl)oxy)ethane (6a): Prepared by following the general experimental procedure with: **1** (0.73 mg, 0.1 mol%), **5a** (88.06 mg, 1 mmol), **3** (0.435 mL, 3 mmol) and NaO^{t}Bu (0.288 mg, 0.3 mol%). **^{1}H-NMR** (300 MHz, $CDCl_3$, 298 K): δ = 3.87 (s, 4H), 1.18 (s, 24H); **$^{13}C\{^1H\}$-NMR** (75 MHz, $CDCl_3$, 298 K): δ = 82.8, 65.1, 24.6; **$^{11}B\{^1H\}$-NMR** (96 MHz, $CDCl_3$, 298 K): δ = 22.27.

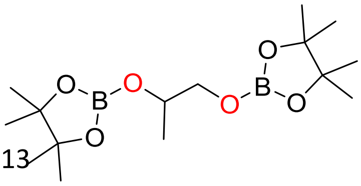

2,2'-(propane-1,2-diylbis(oxy))bis(4,4,5,5-tetramethyl-1,3,2-dioxaborolane) (6b): Prepared by following the general

experimental procedure with: **1** (0.73 mg, 0.1 mol%), **5b** (102.0 mg, 1 mmol), **3** (0.435 mL, 3 mmol) and NaO^{t}Bu (0.288 mg, 0.3 mol%). The obtained analytical data are consistent with those previously reported in the literature.[12]

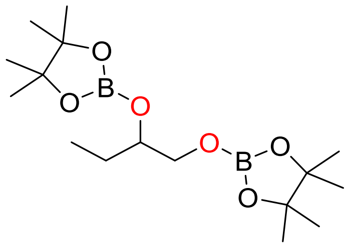

2,2'-(butane-1,2-diylbis(oxy))bis(4,4,5,5-tetramethyl-1,3,2-dioxaborolane) (6c): Prepared by following the general experimental procedure with: **1** (0.73 mg, 0.1 mol%), **5c** (116.12 mg, 1 mmol), **3** (0.435 mL, 3 mmol) and NaO^{t}Bu (0.288 mg, 0.3 mol%). **^{1}H-NMR** (300 MHz, $CDCl_3$, 298 K): δ = 3.96-4.04 (m, 1H), 3.65-3.77 (m, 2H), 1.37-1.50 (m, 2H), 1.19 (s, 12H), 1.18 (s, 12H), 0.85 (t, J = 9 Hz, 3H); **^{13}C{^{1}H}-NMR** (75 MHz, $CDCl_3$, 298 K): δ = 82.8, 82.7, 75.5, 67.8, 25.4, 24.7, 24.6, 9.6; **^{11}B{^{1}H}-NMR** (128 MHz, $CDCl_3$, 298 K): δ = 22.14.

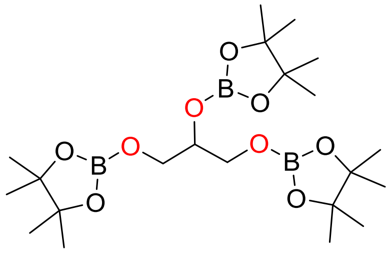

2,2',2''-(propane-1,2,3-triyltris(oxy))tris(4,4,5,5-tetramethyl-1,3,2-dioxaborolane) (6d): Prepared by following the general experimental procedure with: **1** (0.73 mg, 0.1 mol%), **5d** (118.00 mg, 1 mmol), **3** (0.580 mL, 4 mmol) and NaO^{t}Bu (0.288 mg, 0.3 mol%). **^{1}H-NMR** (101 MHz, $CDCl_3$, 298 K): δ = 4.15-4.23 (quin, J = 6 Hz, 1H), 3.74-3.88 (m, 4H), 1.19 (s, 12H), 1.17 (s, 24H); **^{13}C{^{1}H}-NMR** (101 MHz, $CDCl_3$, 298 K): δ = 82.9, 82.8, 73.7, 65.2, 24.6, 24.5; **^{11}B{^{1}H}-NMR** (128 MHz, $CDCl_3$, 298 K): δ = 22.16.

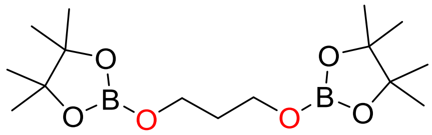

1,3-bis((4,4,5,5-tetramethyl-1,3,2-dioxaborolan-2-yl)oxy)propane (6e): Prepared by following the general experimental procedure with: **1** (0.73 mg, 0.1 mol%), **5e** (102.10 mg, 1 mmol), **3** (0.435 mL, 3 mmol) and NaO^{t}Bu (0.288 mg, 0.3 mol%). **^{1}H-NMR** (300 MHz, $CDCl_3$, 298 K): δ = 3.86 (t, J = 6 Hz, 4H), 1.77 (quin, J = 6 Hz, 2H), 1.17 (s, 24H); **^{13}C{^{1}H}-NMR** (101 MHz, $CDCl_3$, 298 K): δ = 82.8, 61.6, 33.4, 24.7; **^{11}B{^{1}H}-NMR** (128 MHz, $CDCl_3$, 298 K): δ = 22.19.

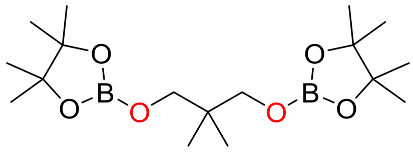

2,2'-((2,2-dimethylpropane-1,3-diyl)bis(oxy))bis(4,4,5,5-tetramethyl-1,3,2-dioxaborolane) (6f): Prepared by following the general experimental procedure with: **1** (0.73 mg, 0.1 mol%), **5f** (130.10 mg, 1 mmol), **3** (0.435 mL, 3 mmol) and NaO^{t}Bu (0.288 mg, 0.3 mol%). **^{1}H-NMR** (300 MHz, $CDCl_3$, 298 K): δ = 3.57 (s, 4H), 1.20 (s, 24H), 0.81 (s, 6H); **^{13}C{^{1}H}-NMR** (101 MHz, $CDCl_3$, 298 K): δ = 82.9, 70.4, 36.8, 21.3, 21.2. **^{11}B{^{1}H}-NMR** (128 MHz, $CDCl_3$, 298 K): δ = 22.03.

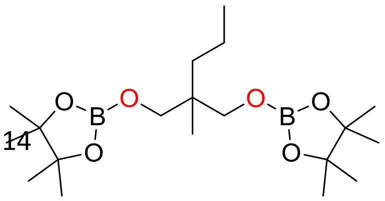

2,2'-((2-methyl-2-propylpropane-1,3-diyl)bis(oxy))bis(4,4,5,5-tetramethyl-1,3,2-dioxaborolane) (6g): Prepared by following the

general experimental procedure with: **1** (0.73 mg, 0.1 mol%), **5g** (158.19 mg, 1 mmol), **3** (0.435 mL, 3 mmol) and NaO^{t}Bu (0.288 mg, 0.3 mol%). **^{1}H-NMR** (300 MHz, $CDCl_3$, 298 K): δ = 3.59 (d, J = 2.4 Hz, 4H), 1.17 (s, 28H), 0.76-0.81 (m, 6H); **^{13}C{^{1}H}-NMR** (101 MHz, $CDCl_3$, 298 K): δ = 82.7, 68.9, 39.2, 36.1, 24.7, 18.4, 16.5, 15.0; **^{11}B{^{1}H}-NMR** (128 MHz, $CDCl_3$, 298 K): δ = 22.21.

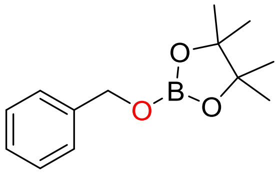

2-(benzyloxy)-4,4,5,5-tetramethyl-1,3,2-dioxaborolane (6h): Prepared by following the general experimental procedure with: **1** (0.73 mg, 0.1 mol%), **5h** (242.10 mg, 1 mmol), **3** (0.435 mL, 3 mmol) and NaO^{t}Bu (0.288 mg, 0.3 mol%). The obtained analytical data are consistent with those previously reported in the literature.[13]

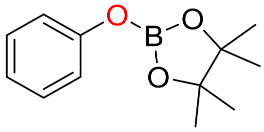

4,4,5,5-tetramethyl-2-phenoxy-1,3,2-dioxaborolane (6i): Prepared by following the general experimental procedure with: **1** (0.73 mg, 0.1 mol%), **5i** (214.10 mg, 1 mmol), **3** (0.435 mL, 3 mmol) and NaO^{t}Bu (0.288 mg, 0.3 mol%). The obtained analytical data are consistent with those previously reported in the literature.[14]

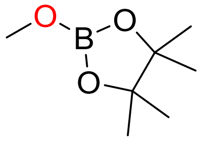

2-methoxy-4,4,5,5-tetramethyl-1,3,2-dioxaborolane (6j): Prepared by following the general experimental procedure with: **1** (0.73 mg, 0.1 mol%), **5j** (90.00 mg, 1 mmol), **3** (0.435 mL, 3 mmol) and NaO^{t}Bu (0.288 mg, 0.3 mol%). The obtained analytical data are consistent with those previously reported in the literature.[15]

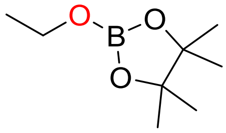

2-ethoxy-4,4,5,5-tetramethyl-1,3,2-dioxaborolane (6k): Prepared by following the general experimental procedure with: **1** (0.73 mg, 0.1 mol%), **5k** (104.11 mg, 1 mmol), **3** (0.435 mL, 3 mmol) and NaO^{t}Bu (0.288 mg, 0.3 mol%). The obtained analytical data are consistent with those previously reported in the literature.[5]

General procedure for catalytic hydroboration of carbon dioxide

Carbon dioxide was purged three times through a schlenk containing a mixture of [Mn]-precatalyst **1** (0.73 mg, 0.036 mol%), NaO^{t}Bu (0.288 mg, 0.1 mol %) and pinacolborane (353.2 mg, 2.76 mmol) in the indicated solvent. Afterwards, the schlenk tube was equipped with a CO_2 balloon and the reaction mixture was heated at the indicated temperatures. The reaction medium was then cooled down to room temperature, and mesitylene was added as an internal standard. Subsequently, a sample (10 μL) of the reaction mixture in $CDCl_3$ (0.4 mL) was subjected to ^{1}H-NMR spectroscopy to determine the yield in alkyl boronate ester.

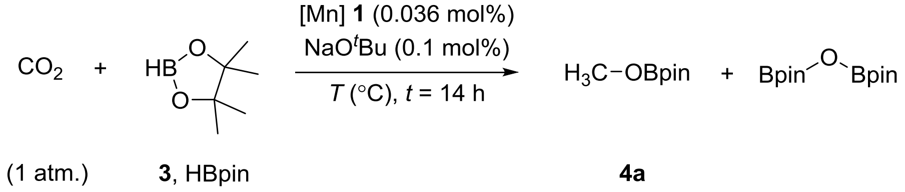

Supplementary Table 4: Optimization studies for the hydroboration of carbon dioxide.

Entry	Temp. (°C)	Solvent (mL)	Yield (%)[a]	TON (4a)
**1**	90	-	76	697
**2**	rt	-	8	70
**3**	90	Toluene (0.5)	52	480
**4**	90	THF (0.5)	12	110
**5**[b]	90	-	83	380
**6**	100	-	96	883
**7**[c]	90	-	4	-
**8**[d]	90	-	3	-

[a] Yields were determined by ^{1}H NMR spectroscopy using mesitylene as an internal standard. [b] Modified conditions: **1** (0.072 mol%), NaO^{t}Bu (0.2 mol%). [c] Modified conditions: Catalyst and base free. [d] Modified conditions: By using NaO^{t}Bu (0.1 mol%) in absence of catalyst.

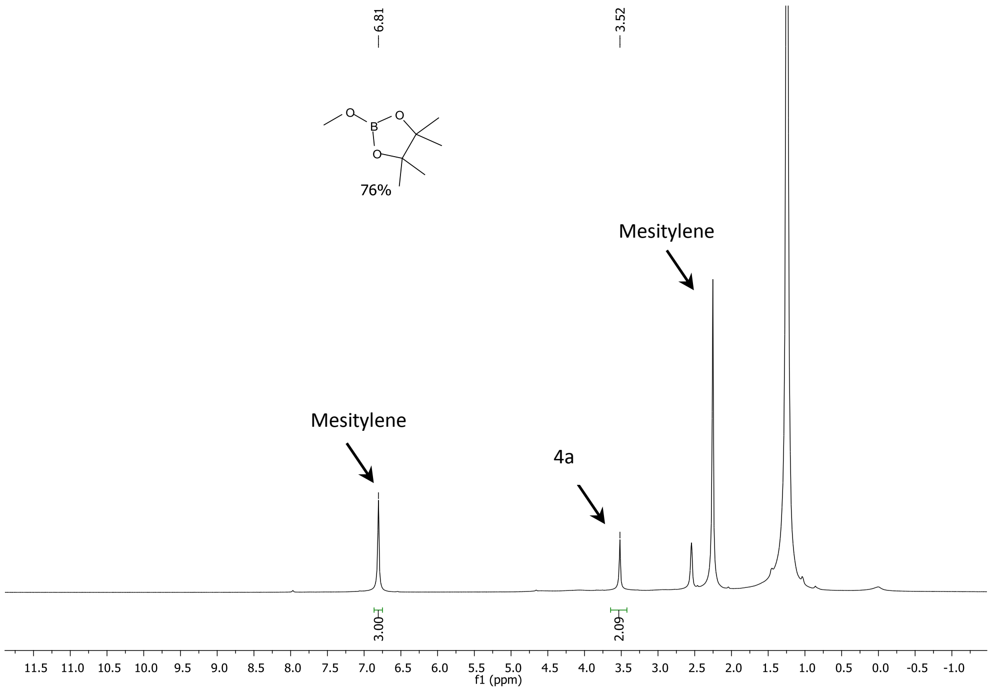

Supplementary Figure 8: ^{1}H-NMR (300 MHz, DMSO-*d*$_6$, 298 K) spectrum for the hydroboration of carbon dioxide under neat conditions, at 90 °C, after 14 h of reaction time, and using mesitylene (0.120 g, 1 mmol) as internal standard (Table 3, entry 1).

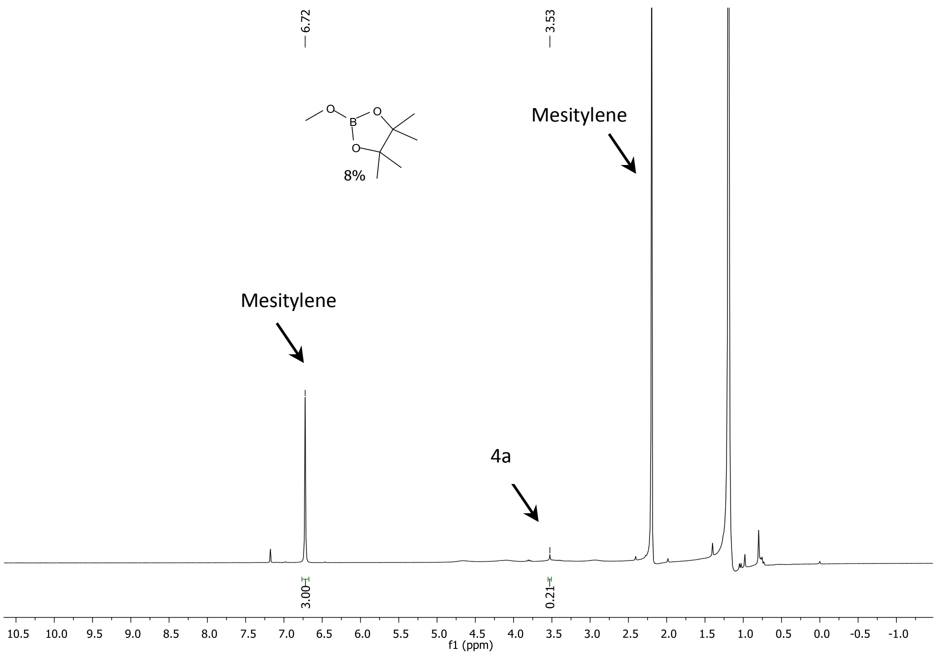

Supplementary Figure 9: ^{1}H-NMR (300 MHz, CDCl3, 298 K) spectrum for the hydroboration of carbon dioxide under neat conditions, at room temperature, after 14 h of reaction time, and using mesitylene (0.120 g, 1 mmol) as internal standard (Table 3, entry 2).

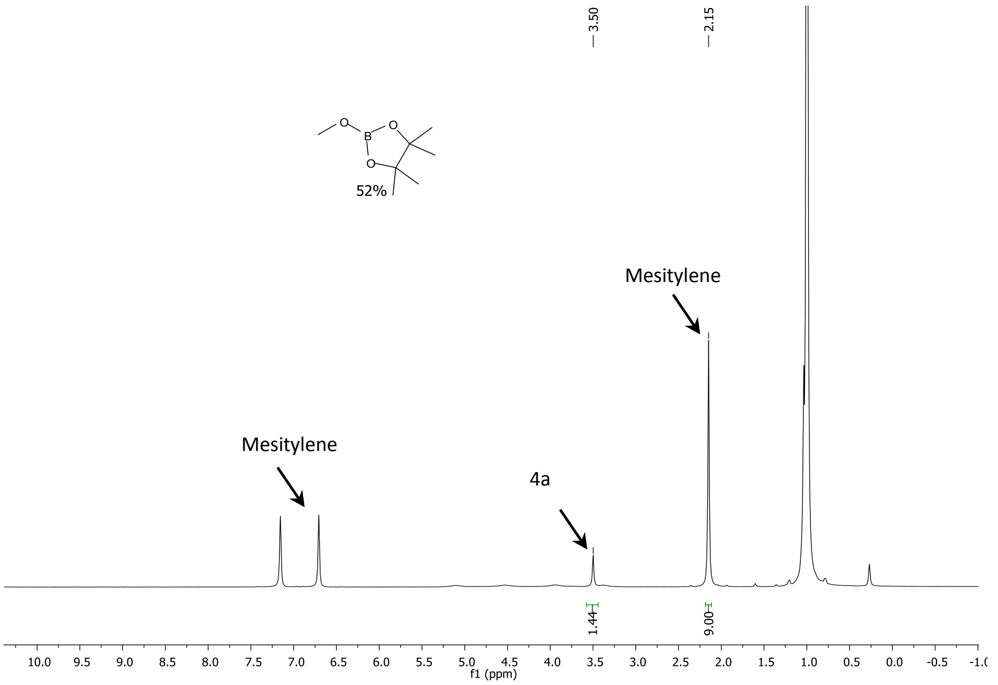

Supplementary Figure 10: ^{1}H-NMR (300 MHz, $CDCl_3$, 298 K) spectrum for the hydroboration of carbon dioxide in toluene, at 90 °C, after 14 h of reaction time, and using mesitylene (0.120 g, 1 mmol) as internal standard (Table 3, entry 3).

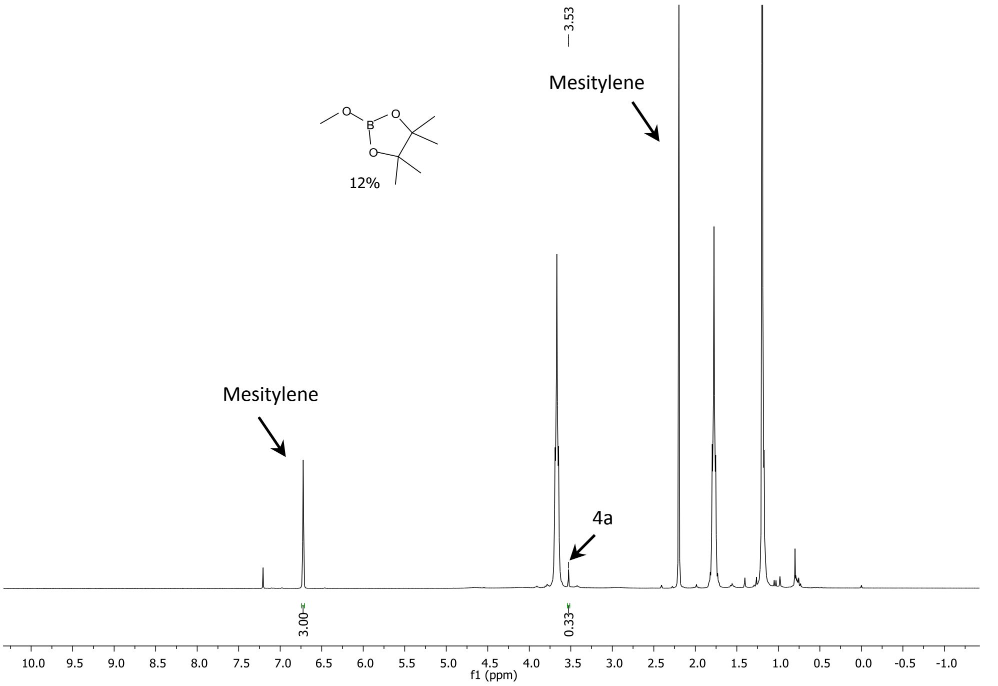

Supplementary Figure 11: ^{1}H-NMR (300 MHz, $CDCl_3$, 298 K) spectrum for the hydroboration of carbon dioxide in THF, at 90 °C, after 14 h of reaction, and using mesitylene (0.120 g, 1 mmol) as internal standard (Table 3, entry 4).

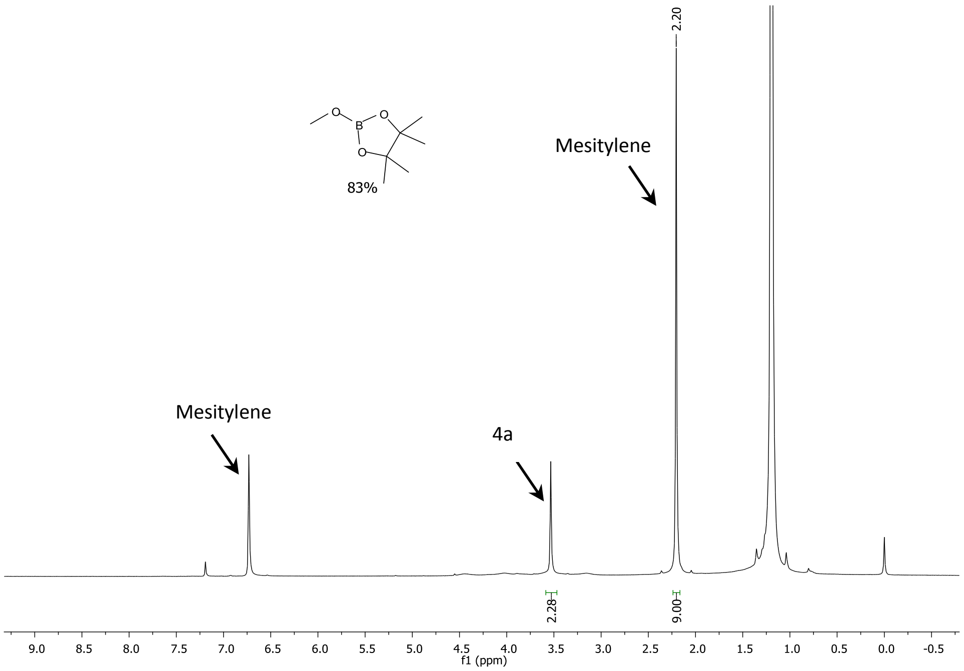

Supplementary Figure 12: ^{1}H-NMR (300 MHz, $CDCl_3$, 298 K) spectrum for the hydroboration of carbon dioxide under neat conditions, at room temperature, after 14 h of reaction, and by using 1 (0.072 mol%), NaO^{t}Bu (0.2 mol%) Mesitylene (0.120 g, 1mmol) (Table 3, entry 5).

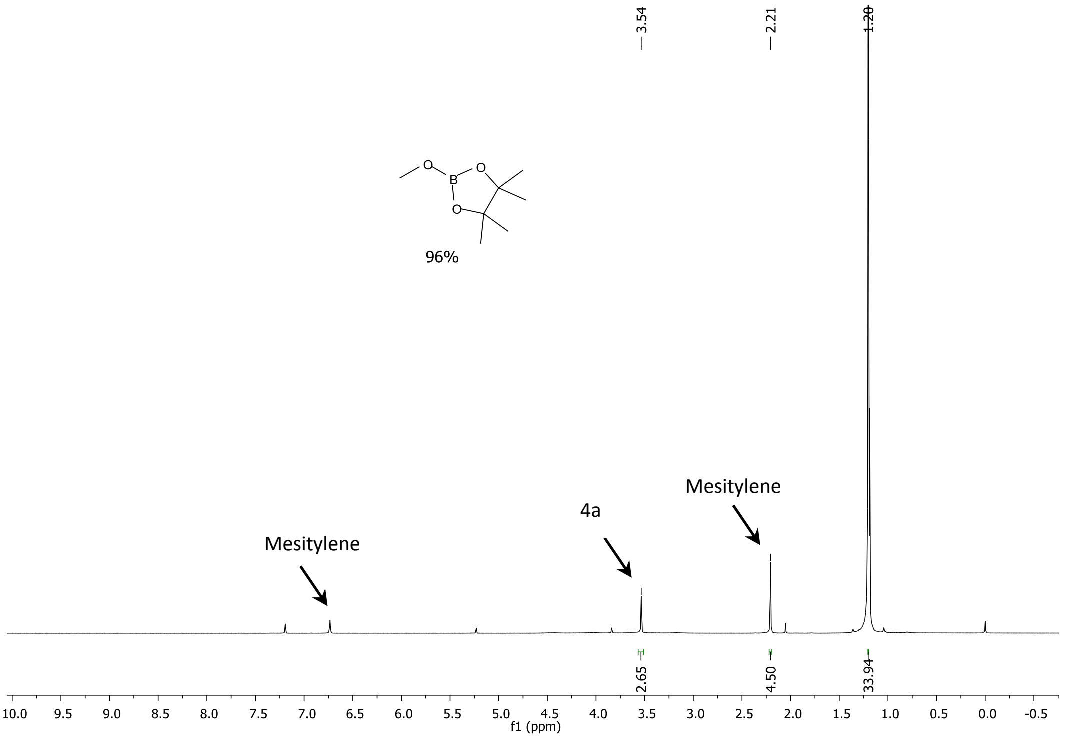

Supplementary Figure 13: ^{1}H-NMR (400 MHz, $CDCl_3$, 298 K) spectrum for the hydroboration of carbon dioxide under neat conditions at 100 °C after 14 h of reaction, and using mesitylene (0.060 g, 0.5 mmol) as internal standard (Table 3, entry 6).

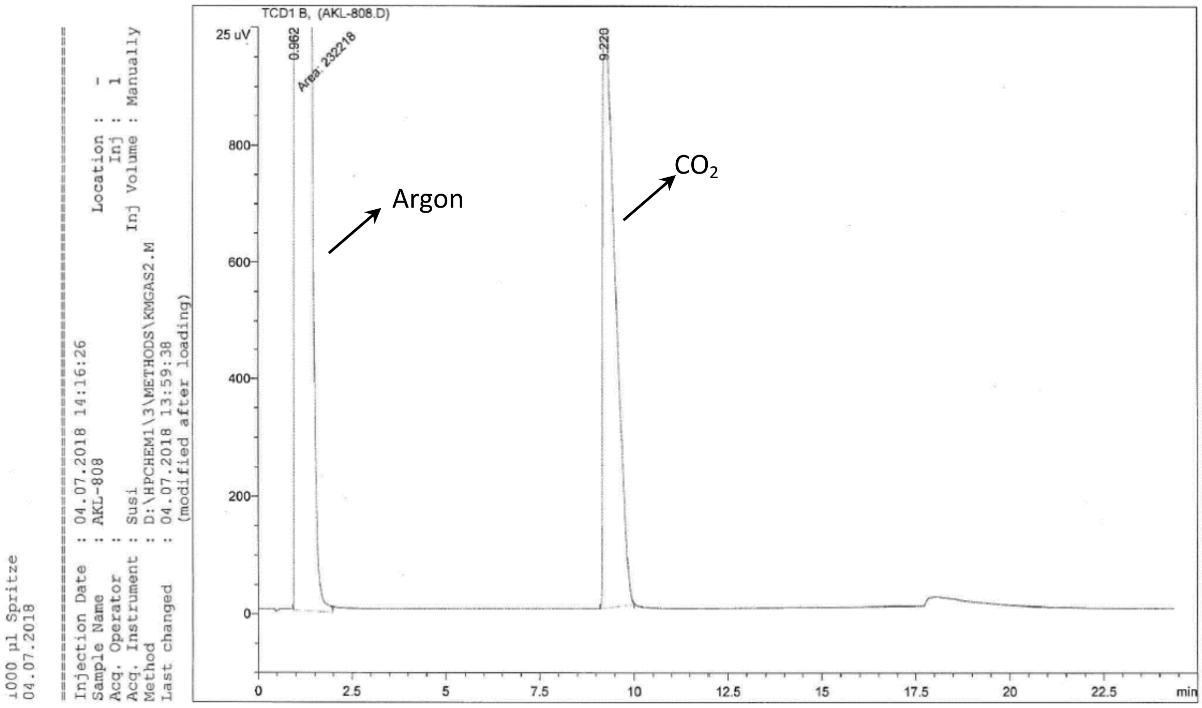

Supplementary Figure 14: Gas phase spectrum for the CO_2 hydroboration.

Observation of catalytic intermediates

Observation of complex (I)

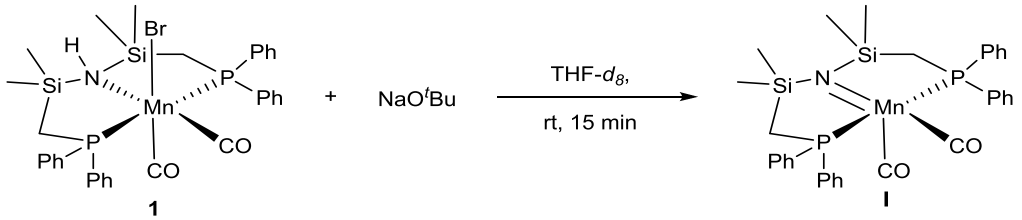

1 **I**

Sodium *tert*-butoxide (2.403 mg, 0.025 mmol) was added to a THF (0.4 mL) solution of complex **1** (15 mg, 0.021 mmol). The resulting mixture was stirred for fifteen minutes at room temperature leading to a colour change from yellow to dark violet. Subsequent filtration over celite, followed by removal of the solvent in vacuo led to complex **I** (9.8 mg, 0.0154 mmol, 74%) as a dark violet solid. Several attempts were performed to obtain single crystals of complex **I**, but were proven so far unsuccessful. 1H-NMR (300 MHz, THF-d_8, 298 K): δ = 7.66-7.74 (m, 8H), 7.35-7.36 (m, 12H), 1.84 (t, 4H, J = 6 Hz), -0.02 (s, 12H). **^{13}C{^{1}H}-NMR**^{a} (75 MHz, THF-d_8, 298 K): δ = 133.6 (vt, J = 5.4 Hz), 130.4 (br s), 129.8 (s), 129.1 (vt, J= 4.4 Hz), 8.1 (s), 5.9 (s). **^{31}P{^{1}H}-NMR** (121 MHz, THF-d_8, 298 K): δ = 61.26. **HRMS (ESI$^+$):** *m/z*: calcd. for $C_{32}H_{37}MnNP_2Si_2$ [M+H]$^+$: 640.12131; found: 640.12119.[a] The presumably broad ^{13}C signals of the carbonyl groups could not be located even after increasing the acquisition time or using two-dimensional NMR experiments.

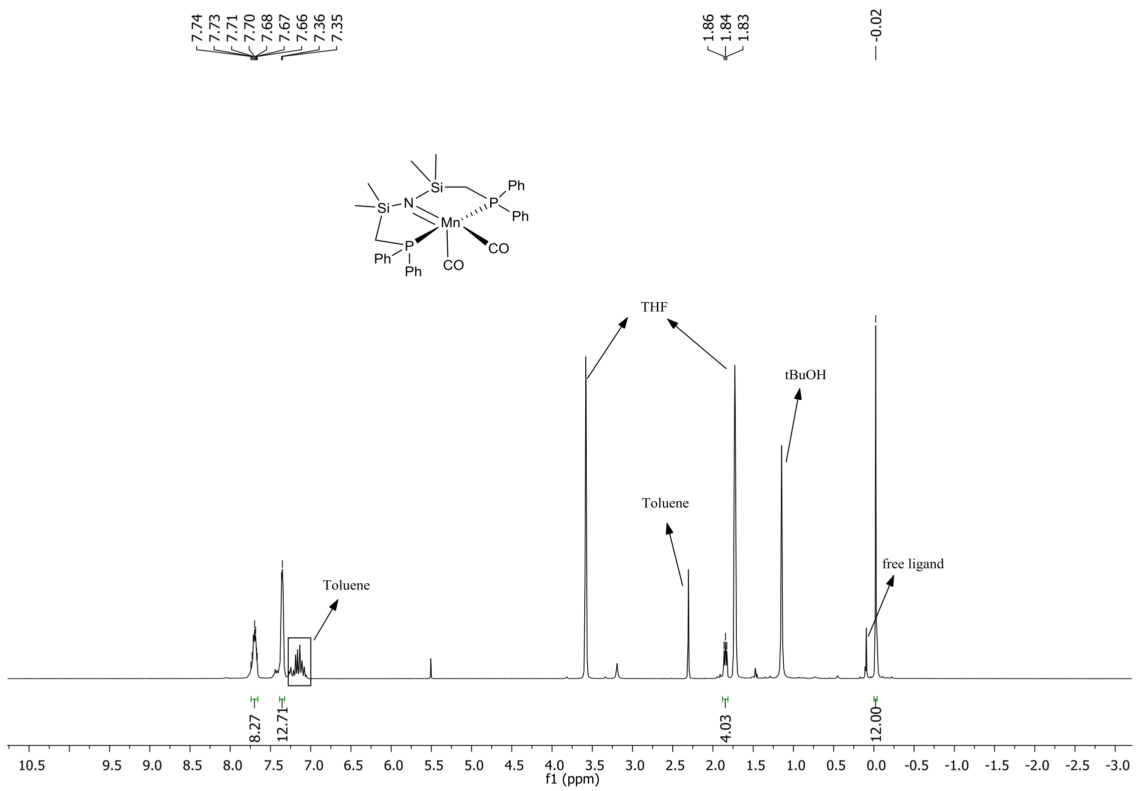

Supplementary Figure 15: ^{1}H-NMR (300 MHz, THF-d_8, 298 K) spectrum of complex I.

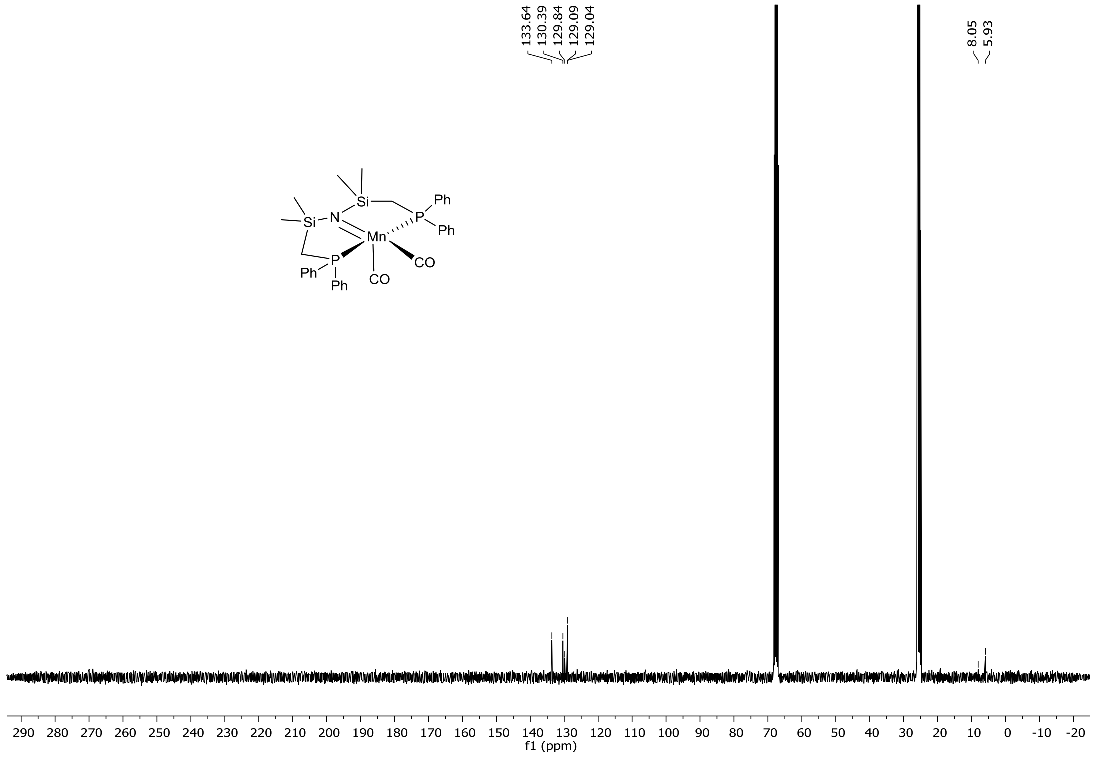

Supplementary Figure 16: ^{13}C{^{1}H}-NMR (75 MHz, THF-d_8, 298 K) spectrum of complex I.

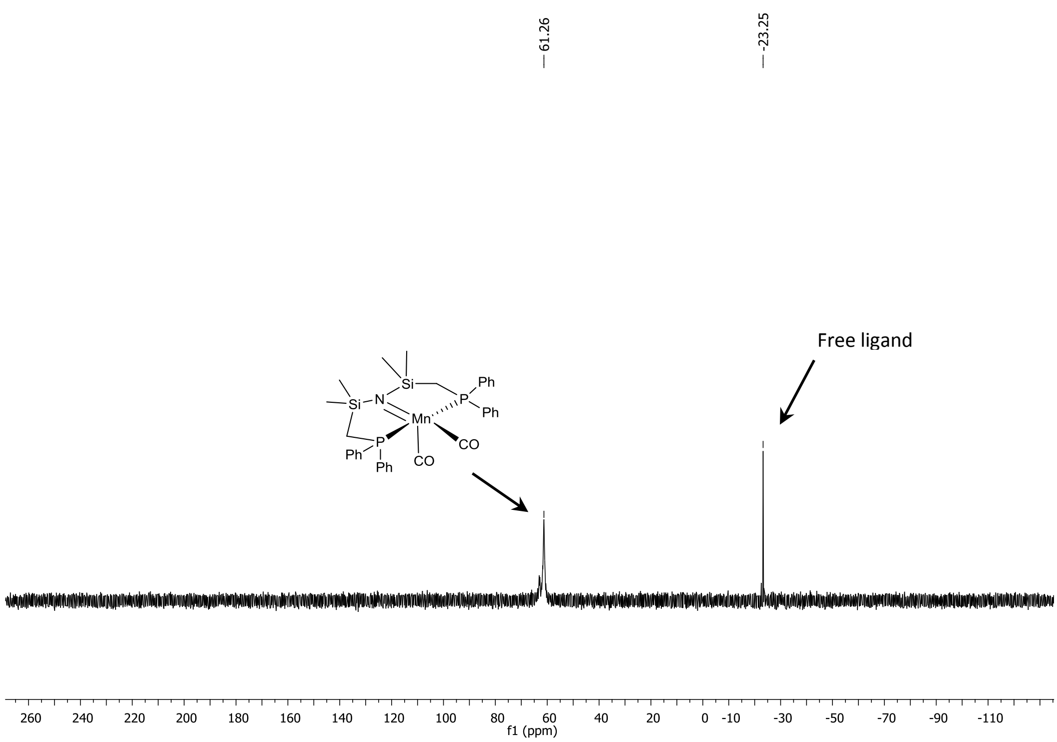

Supplementary Figure 17: $^{31}P\{^{1}H\}$-NMR (121 MHz, THF-d_8, 298 K) spectrum of complex I.

Stoichiometric reaction of complex (I) with pinacolborane:

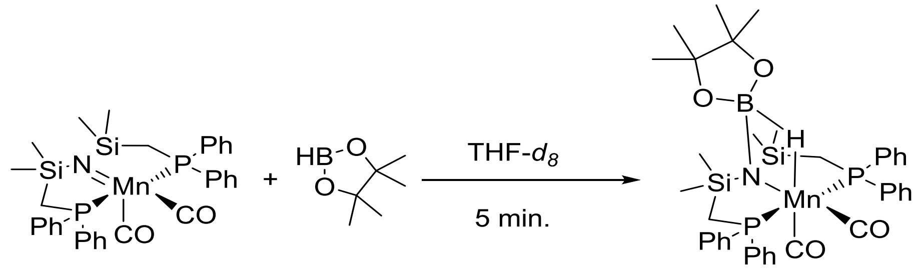

I **IIa**

The addition of complex **I** (13.5 mg, 0.021 mmol) to a solution of pinacolborane (3.9 mg, 0.030 mmol) in THF-d_8 (0.4 mL) lead to a concomitant color change from dark violet to yellow. ^{1}H-NMR analysis revealed a broad signal at -6.57 ppm that can be attributed to complex **IIa**, where a pinacolborane bridges the manganese atom with the nitrogen from the ligand framework. This results in the formation of a metal hydride. Furthermore, $^{31}P\{^{1}H\}$ and $^{11}B\{^{1}H\}$-NMR analysis revealed a new peak at 65.20 ppm and 21.35 ppm, respectively. The formation of complex **IIa**, could be confirmed by X-Ray analysis of single crystals grown by the slow diffusion of hexane into a concentrated solution of dichloromethane.

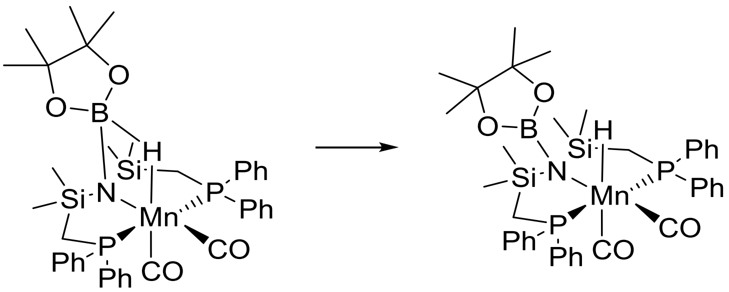

IIa **II**

When complex **IIa** is heated at 50 °C for 30 min, a new signal in the hydride region of the ^{1}H-NMR spectrum is observed (-8.14 ppm, t, J = 32 Hz). The associated ^{31}P{^{1}H}-NMR spectrum also revealed the apparition of a new signal at 62.31 ppm. These signals can be attributed to the formation of **II**. Interestingly, after 2 days of reaction, most of **IIa** converts into **II**.

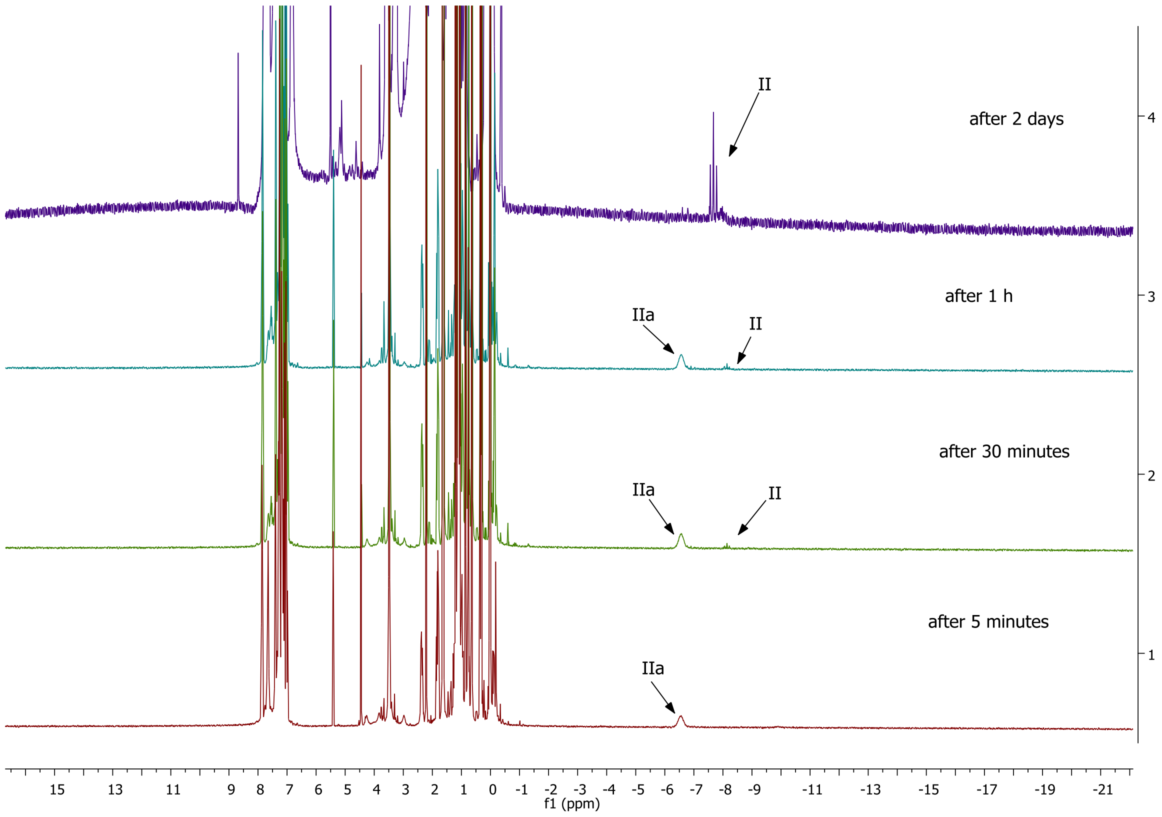

Supplementary Figure 18: Superposed ^{1}H-NMR spectra in THF-d_8 of intermediates IIa and II at different time intervals.

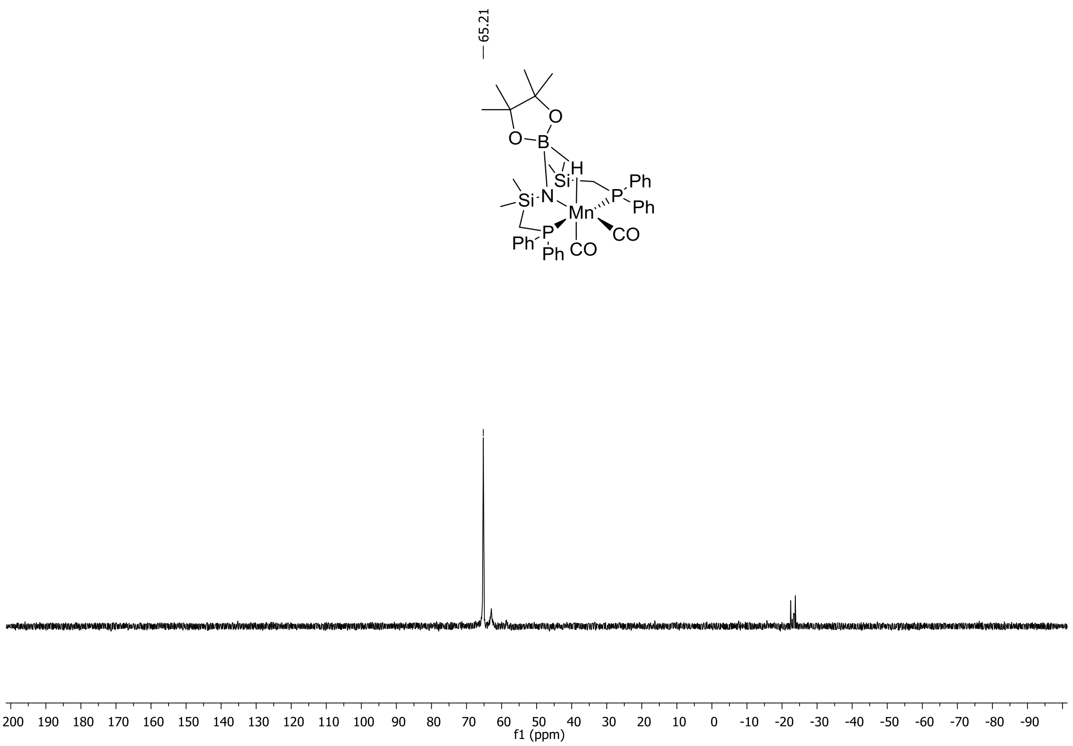

Supplementary Figure 19: $^{31}P\{^{1}H\}$-NMR (162 MHz, THF-d_8, 298 K) spectrum of complex IIa.

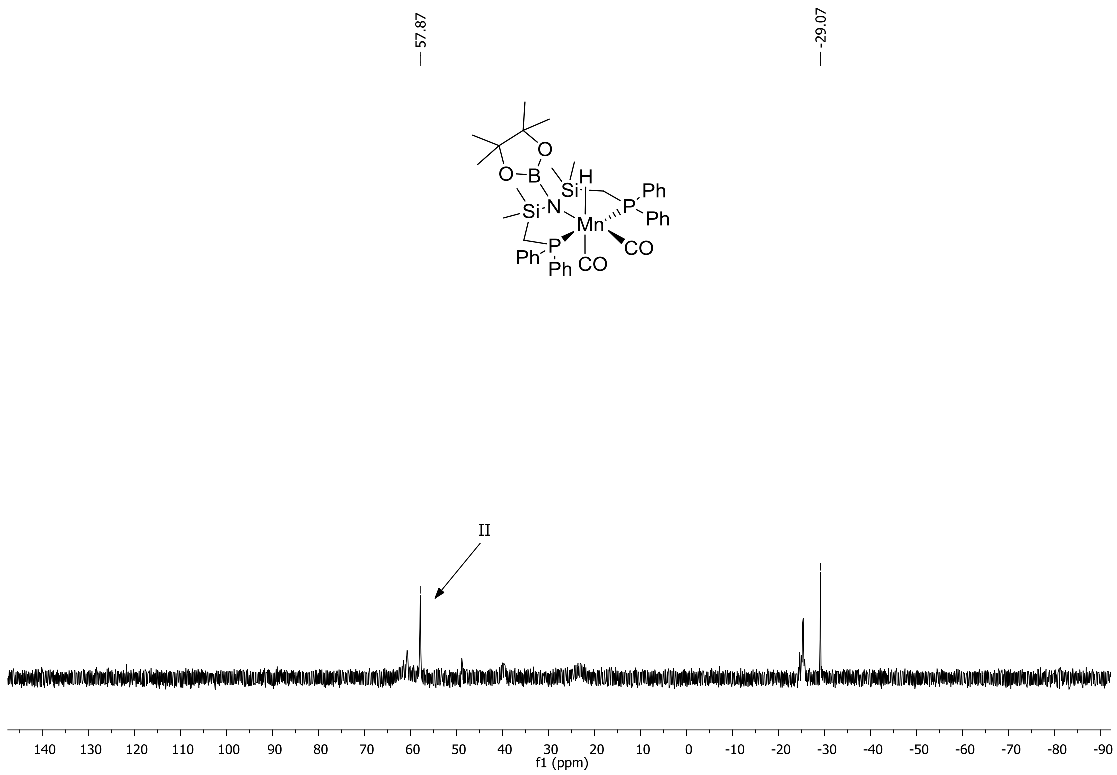

Supplementary Figure 20: $^{31}P\{^{1}H\}$-NMR (121 MHz, THF-d_8, 298 K) spectrum of reaction mixture of complex II after 2 days.

Observation of reaction intermediates from dimethyl carbonate

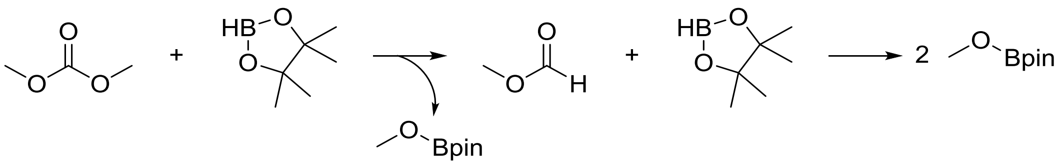

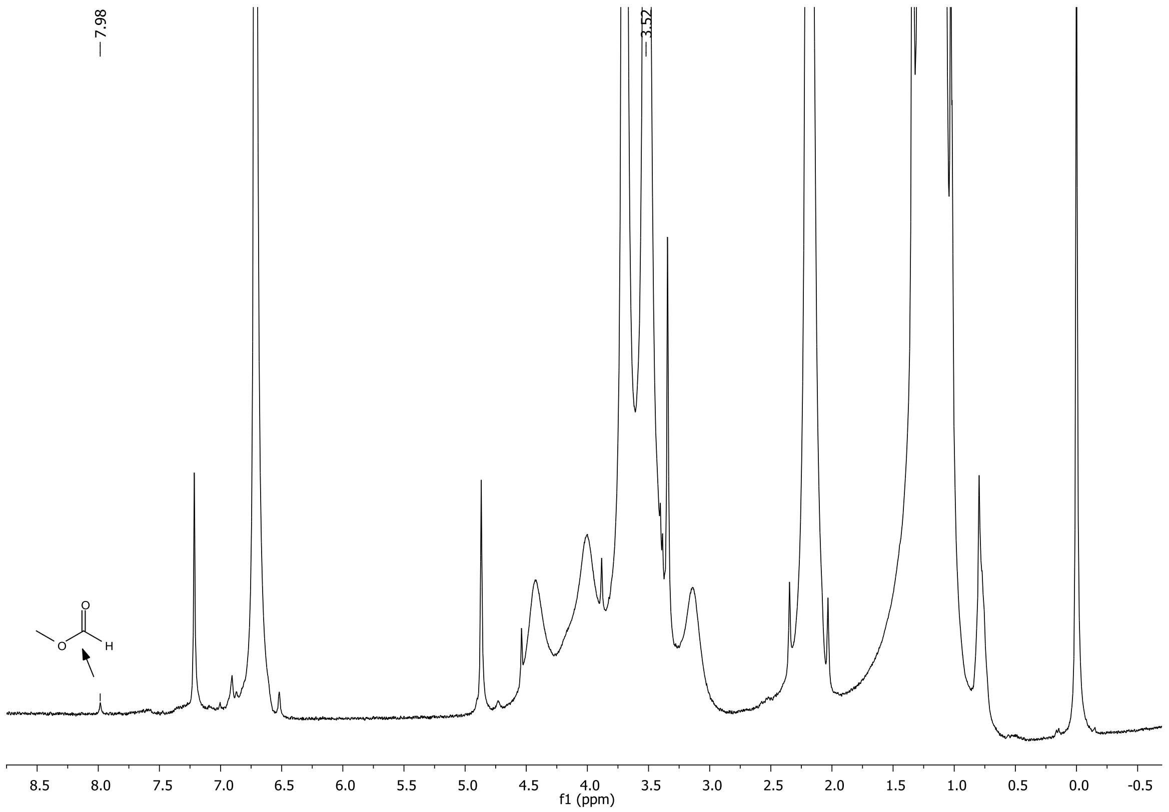

Supplementary Figure 21: ^{1}H-NMR (300 MHz, $CDCl_3$, 298 K) spectrum of the reaction between dimethyl carbonate with pinacolborane in the presence of complex 1 and base.

Observation of reaction intermediates from carbon dioxide

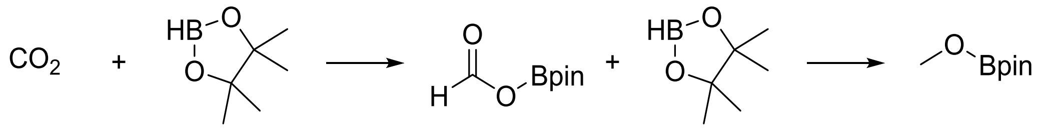

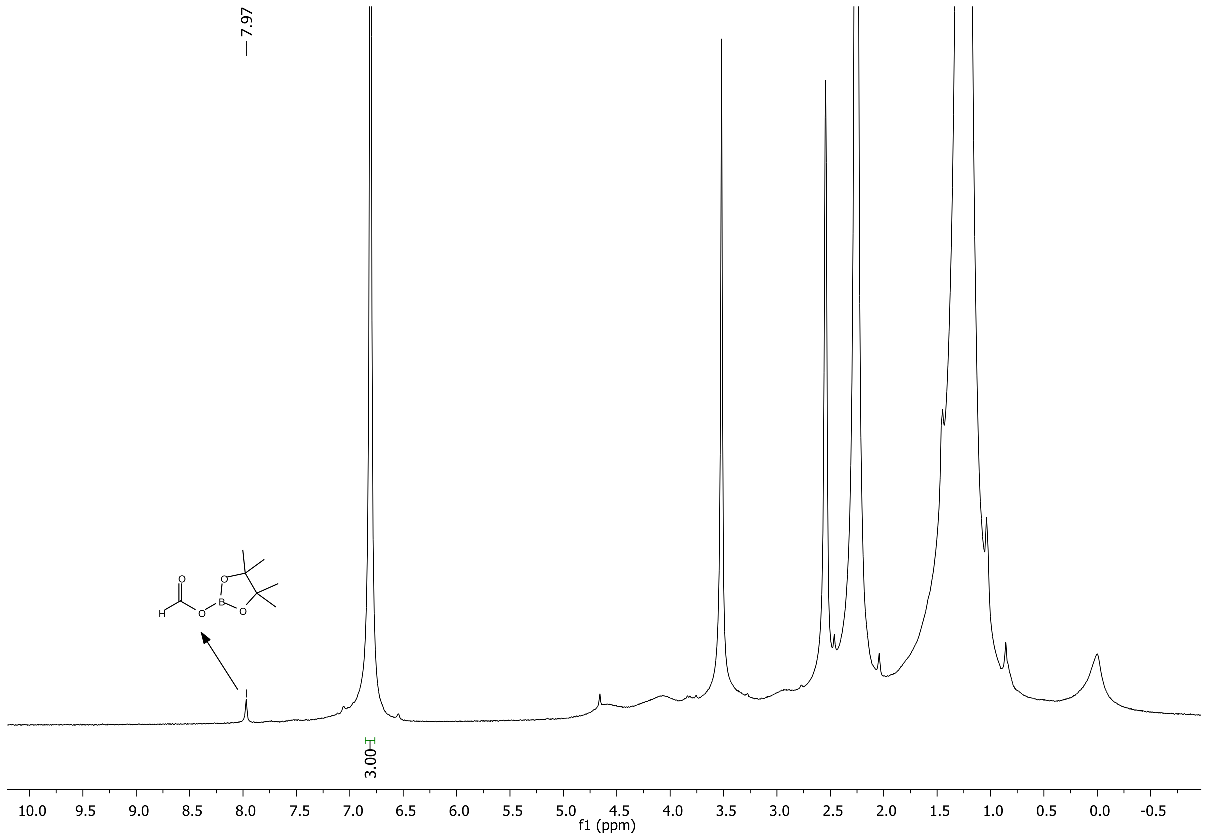

Supplementary Figure 22: ^{1}H-NMR (300 MHz, DMSO-*d*$_6$, 298 K) spectrum of the reaction between carbon dioxide with pinacolborane in the presence of complex 1 and base.

NMR-Spectra

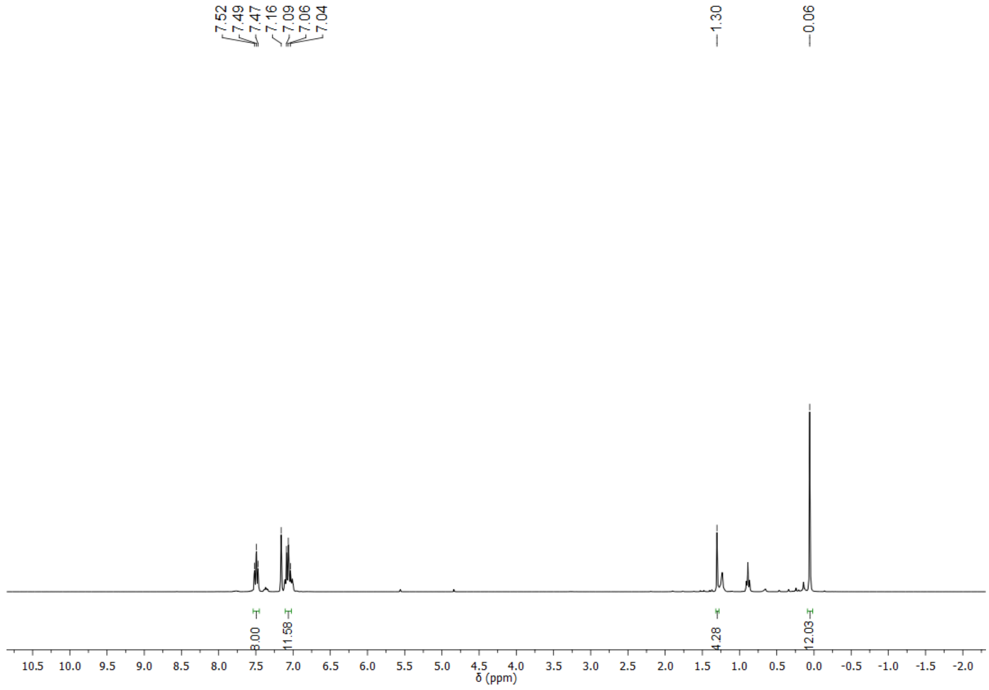

Supplementary Figure 23: ^{1}H-NMR (300 MHz, C_6D_6, 298 K) of 1, 3-bis(diphenyl-phosphino)methyl)tetramethyldisilazane.

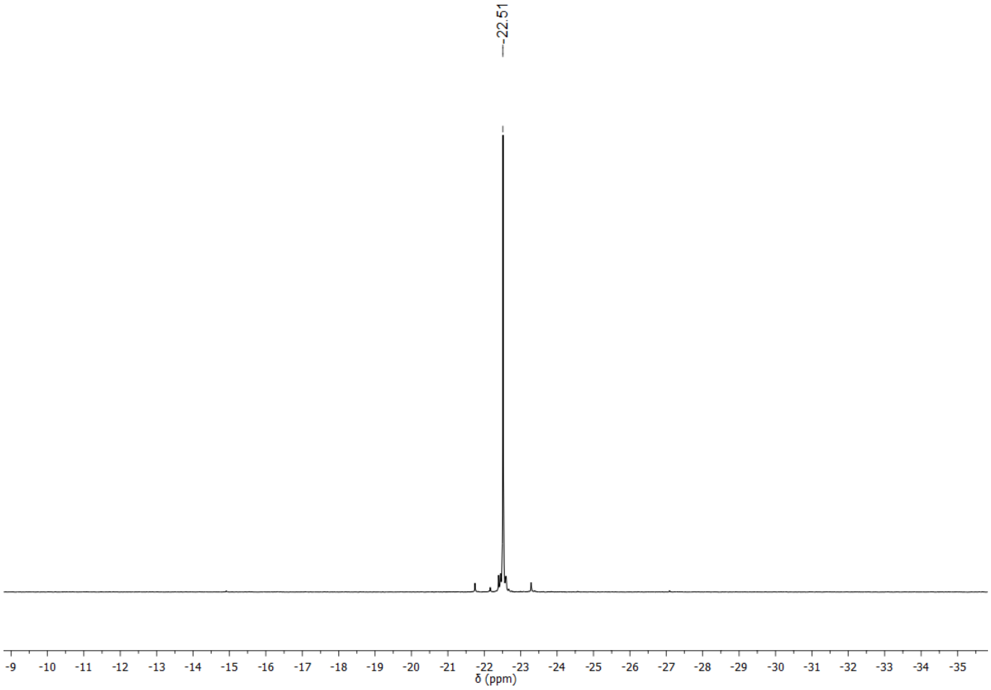

Supplementary Figure 24: ^{31}P{^{1}H}-NMR (121 MHz, C_6D_6, 298 K) of 1, 3-bis(diphenyl-phosphino)methyl)tetramethyldisilazane.

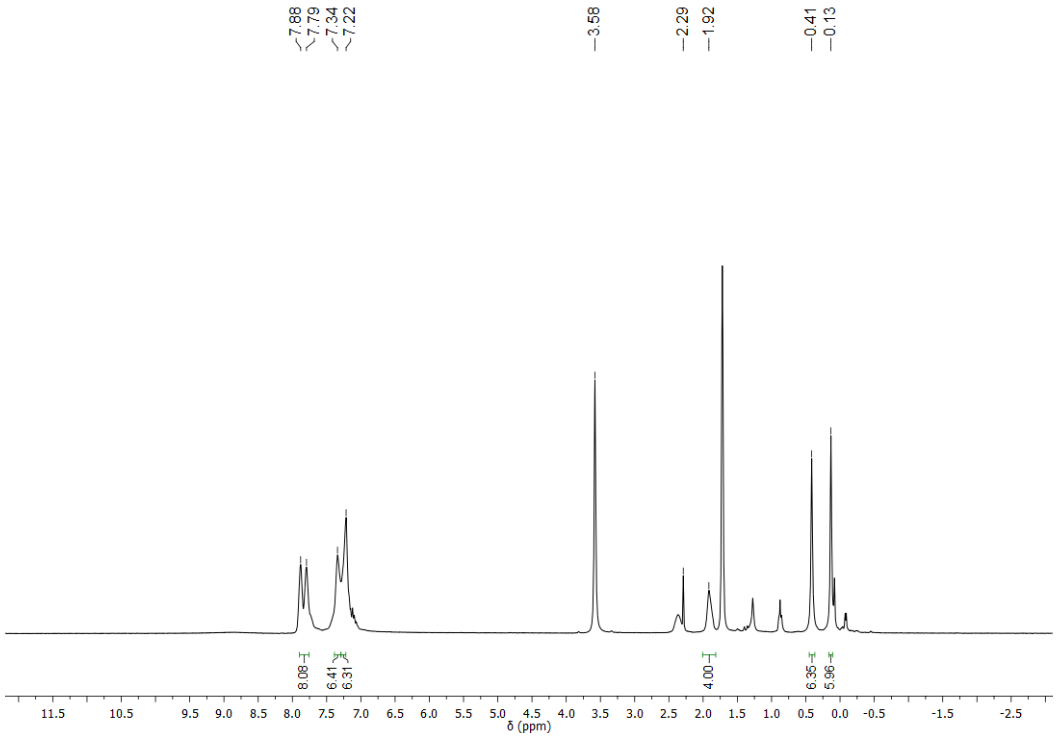

Supplementary Figure 25: ^{1}H-NMR (300 MHz, THF-d_8, 298 K) of 1.

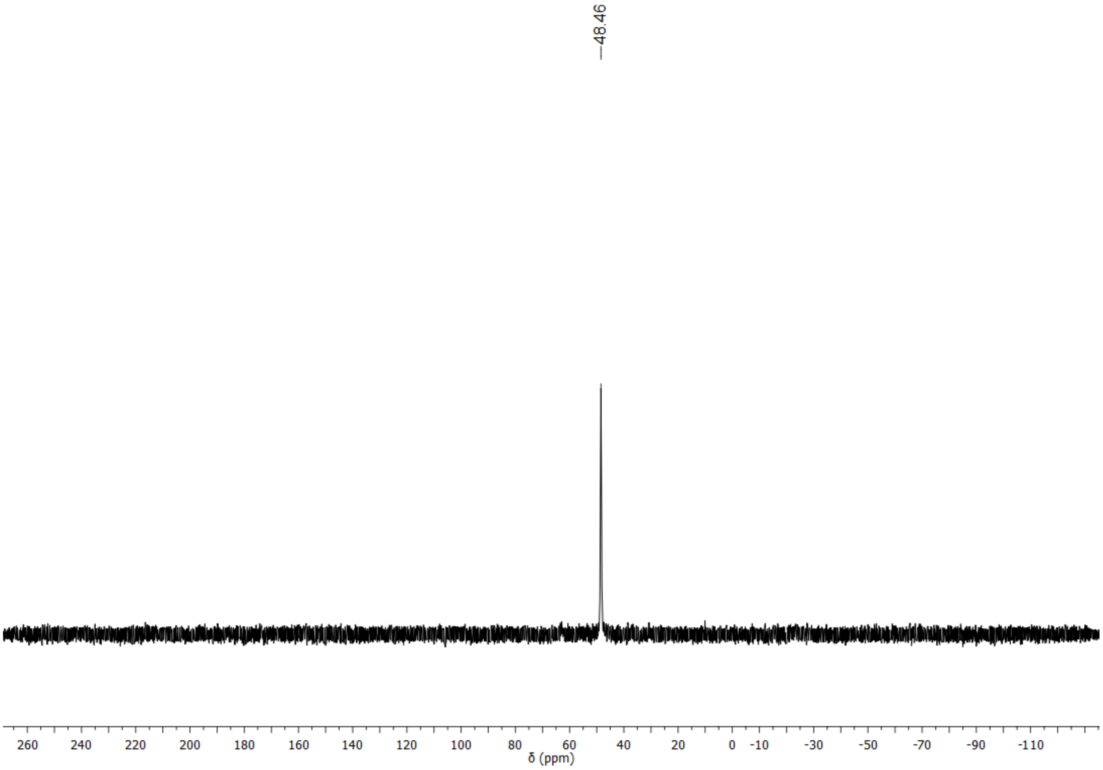

Supplementary Figure 26: ^{31}P{^{1}H}-NMR (121 MHz, THF-d_8, 298 K) of 1.

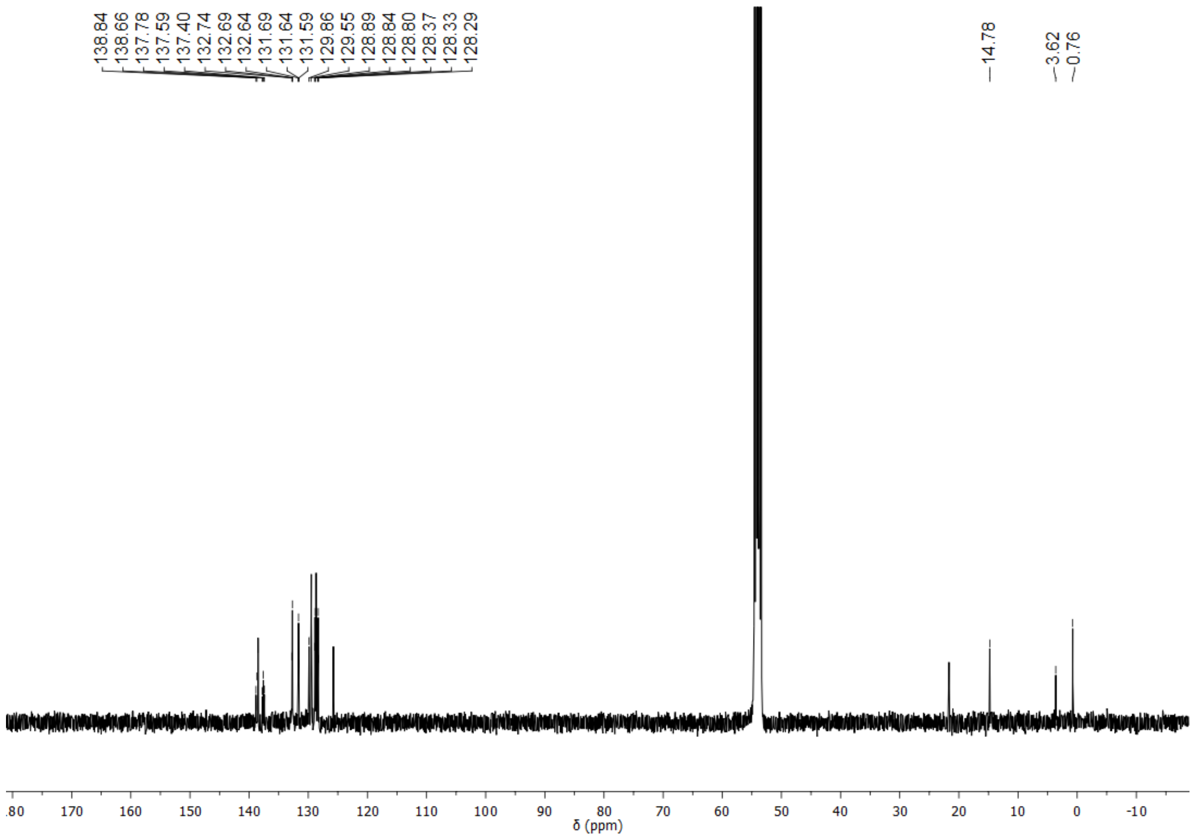

Supplementary Figure 27: $^{13}C\{^1H\}$-NMR (101 MHz, THF-d_8, 298 K) of 1.

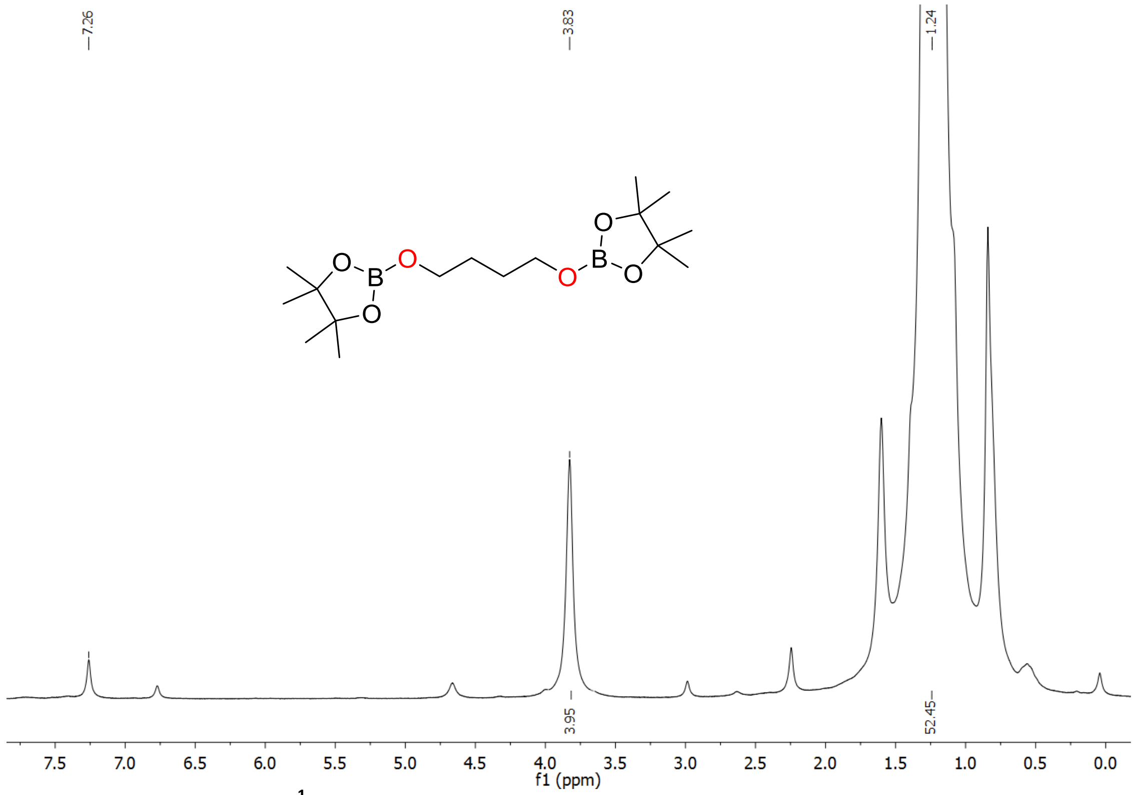

Supplementary Figure 28: ^{1}H-NMR (400 MHz, $CDCl_3$, 296K) of 4e.

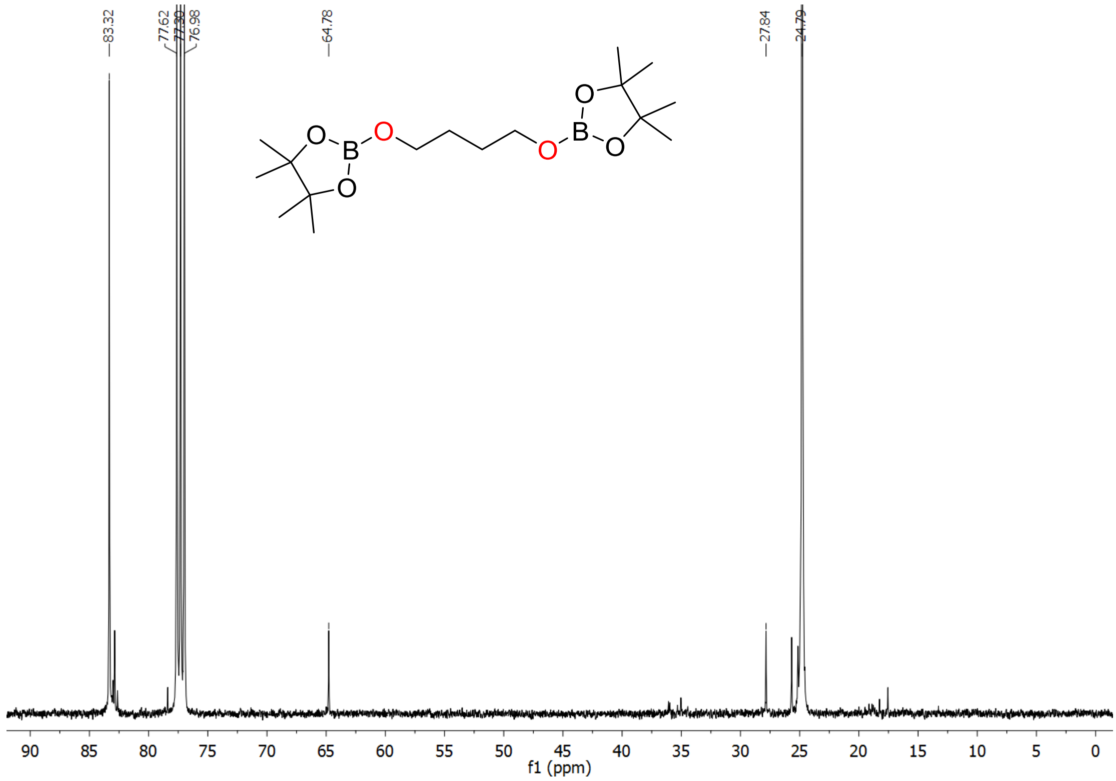

Supplementary Figure 29: $^{13}C\{^{1}H\}$-NMR (101 MHz, $CDCl_3$, 296K) of 4e.

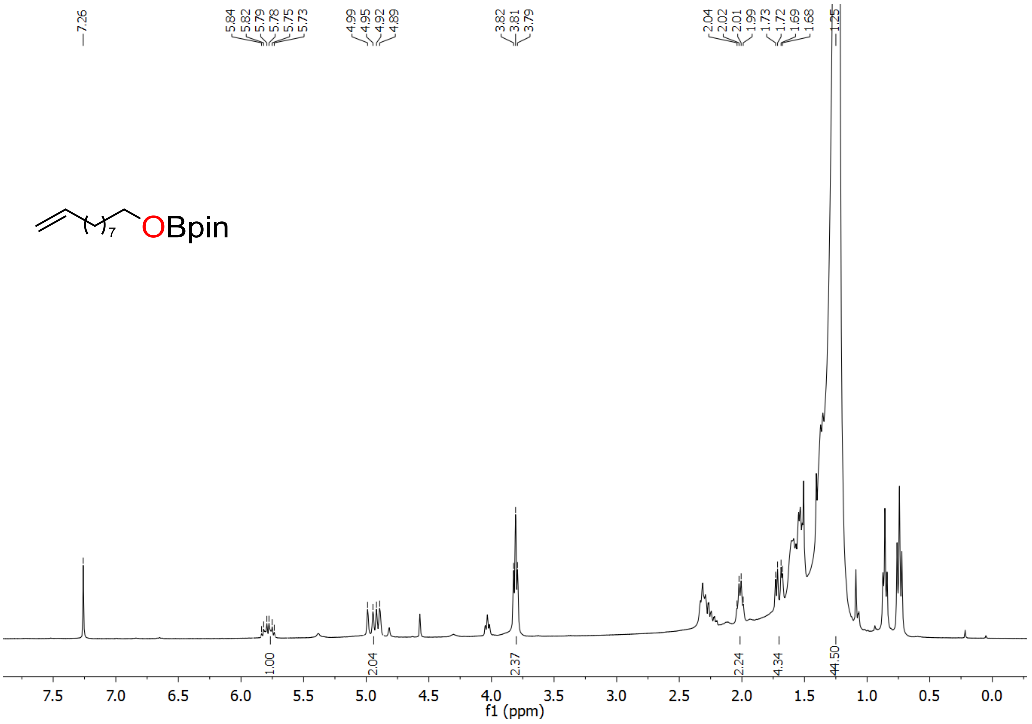

Supplementary Figure 30: ^{1}H-NMR (400 MHz, $CDCl_3$, 296K) of 4g.

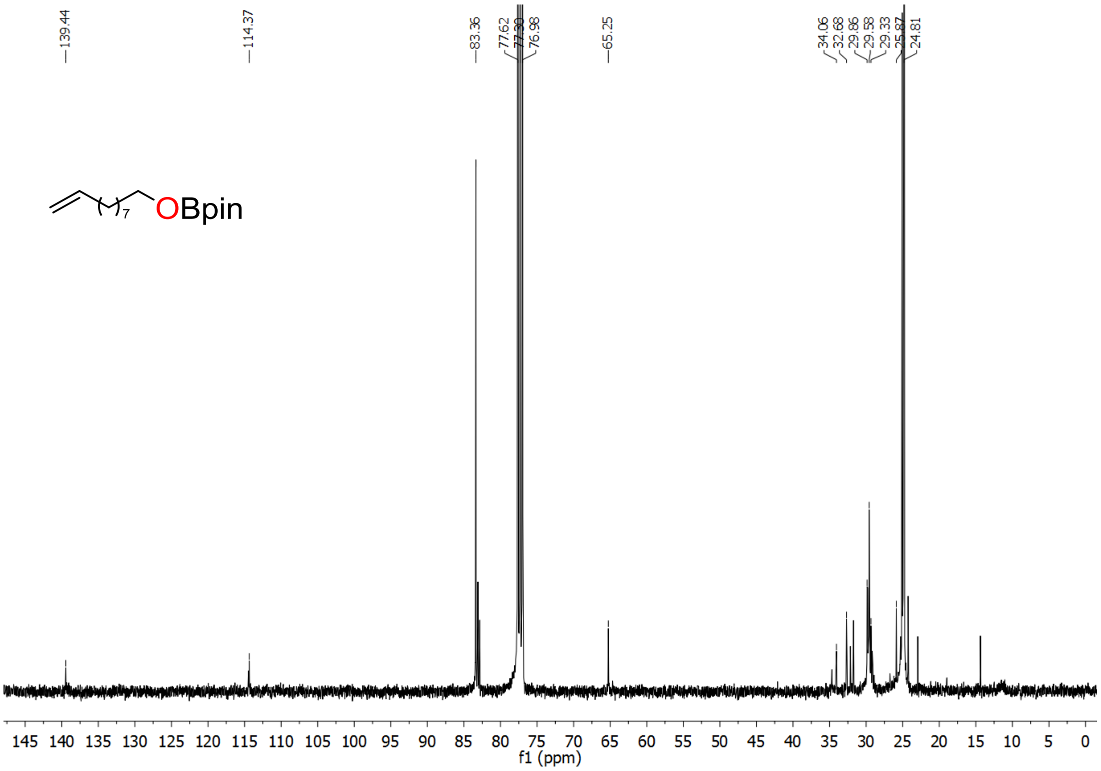

Supplementary Figure 31: $^{13}C\{^{1}H\}$-NMR (101 MHz, $CDCl_3$, 296K) of 4g.

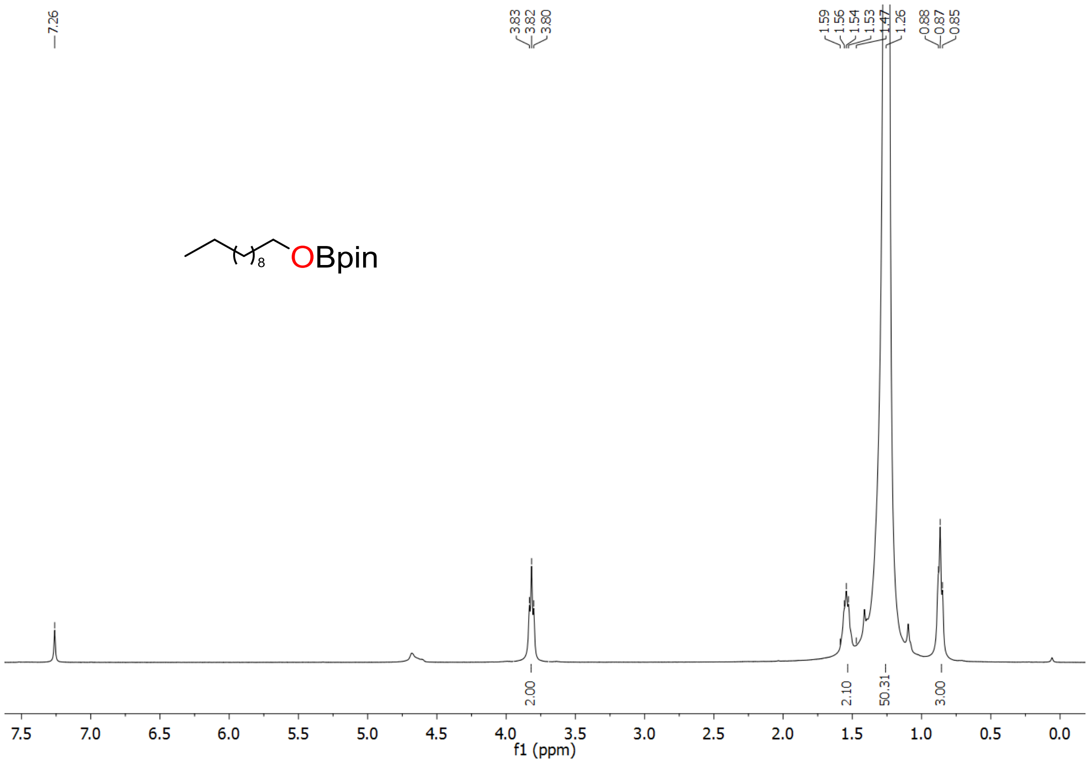

Supplementary Figure 32: ^{1}H-NMR (400 MHz, $CDCl_3$, 296K) of 4i.

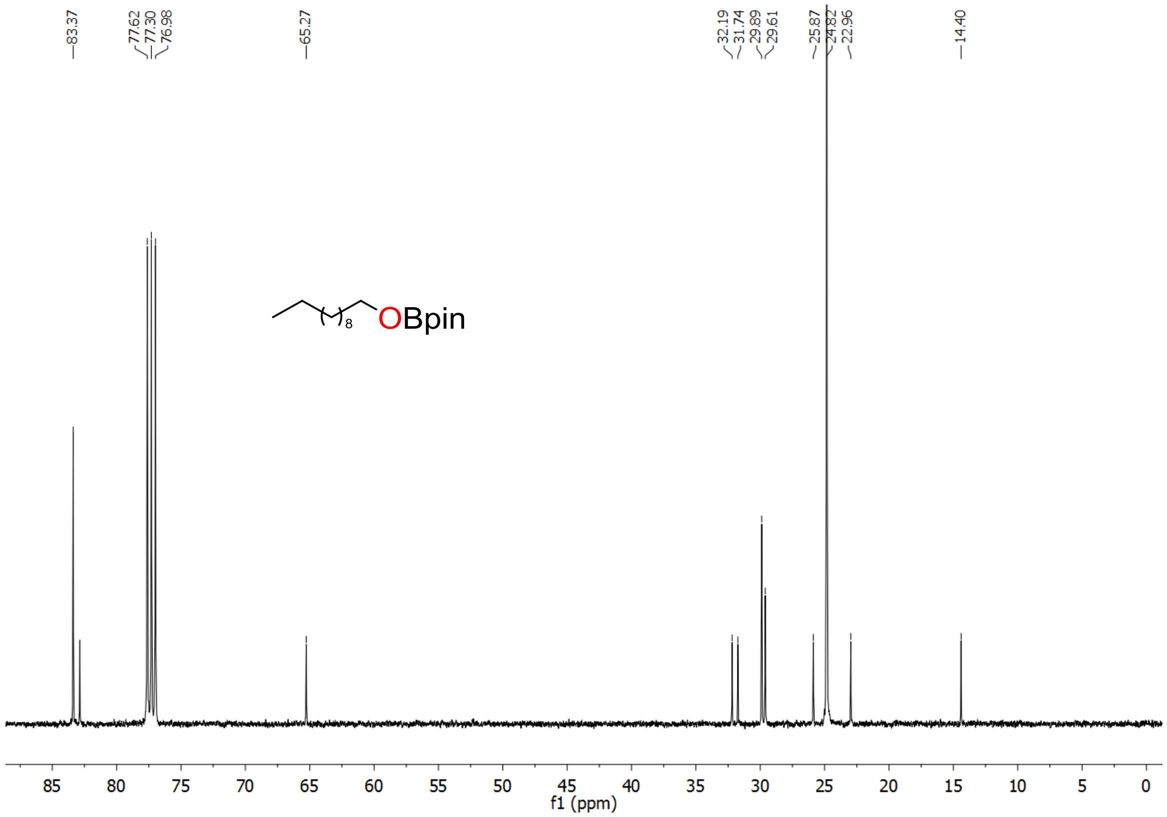

Supplementary Figure 33: $^{13}C\{^{1}H\}$-NMR (101 MHz, $CDCl_3$, 296K) of 4i.

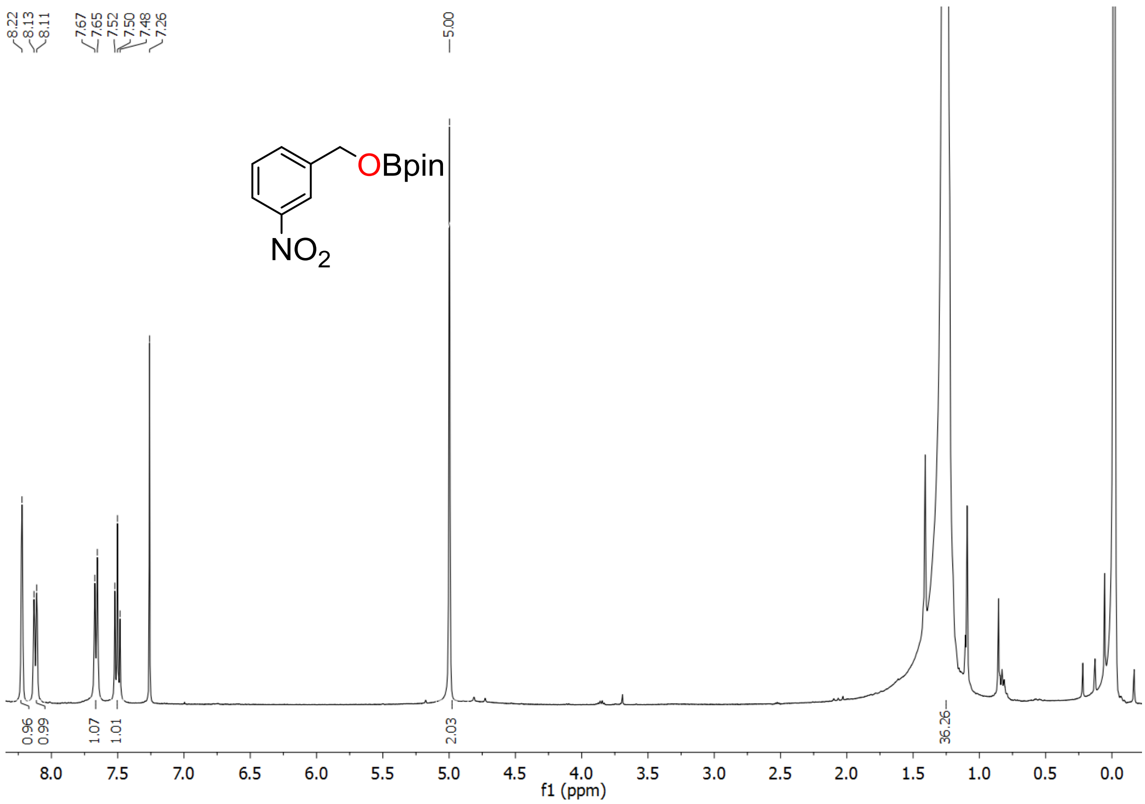

Supplementary Figure 34: ^{1}H-NMR (400 MHz, $CDCl_3$, 296K) of 4k.

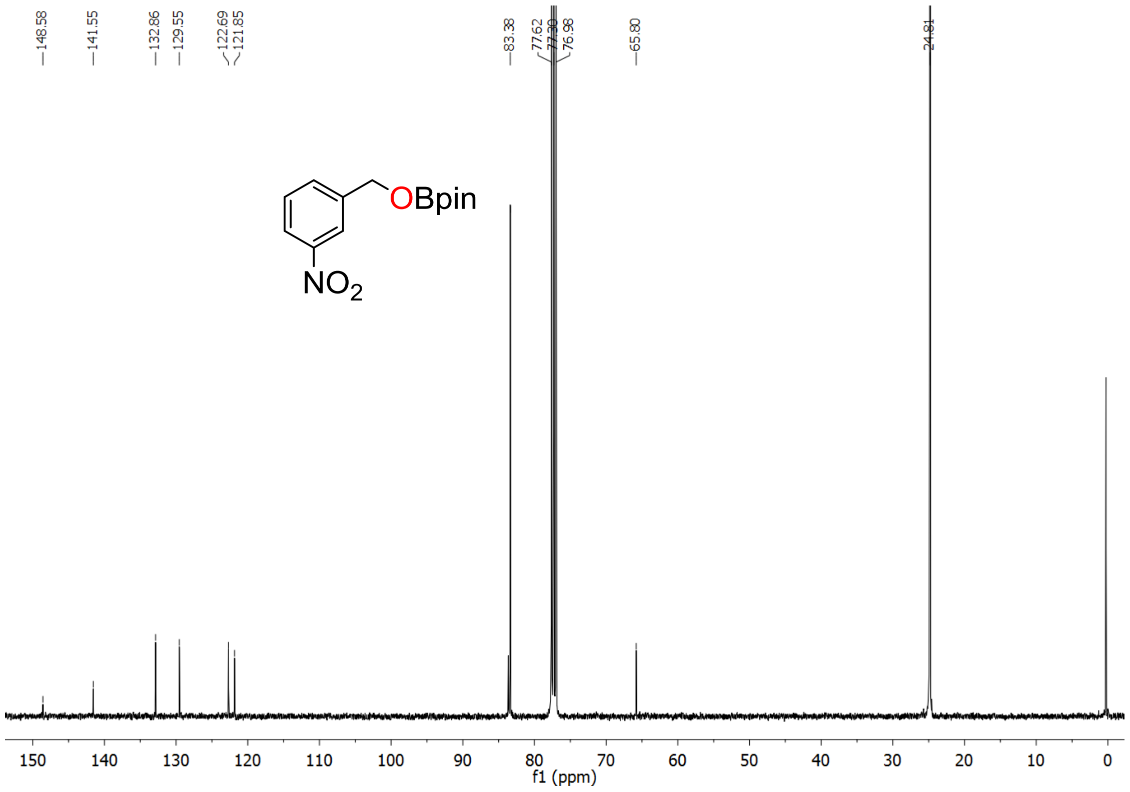

Supplementary Figure 35: $^{13}C\{^{1}H\}$-NMR (101 MHz, $CDCl_3$, 296K) of 4k.

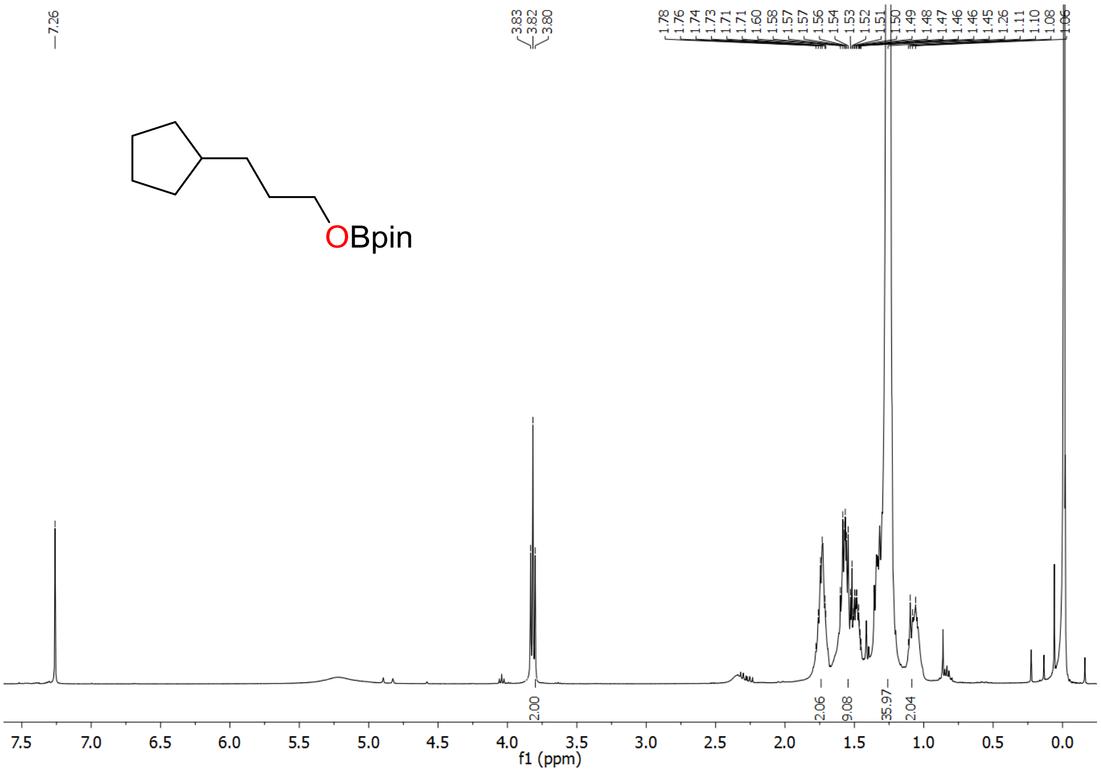

Supplementary Figure 36: ^{1}H-NMR (400 MHz, $CDCl_3$, 296K) of 4m.

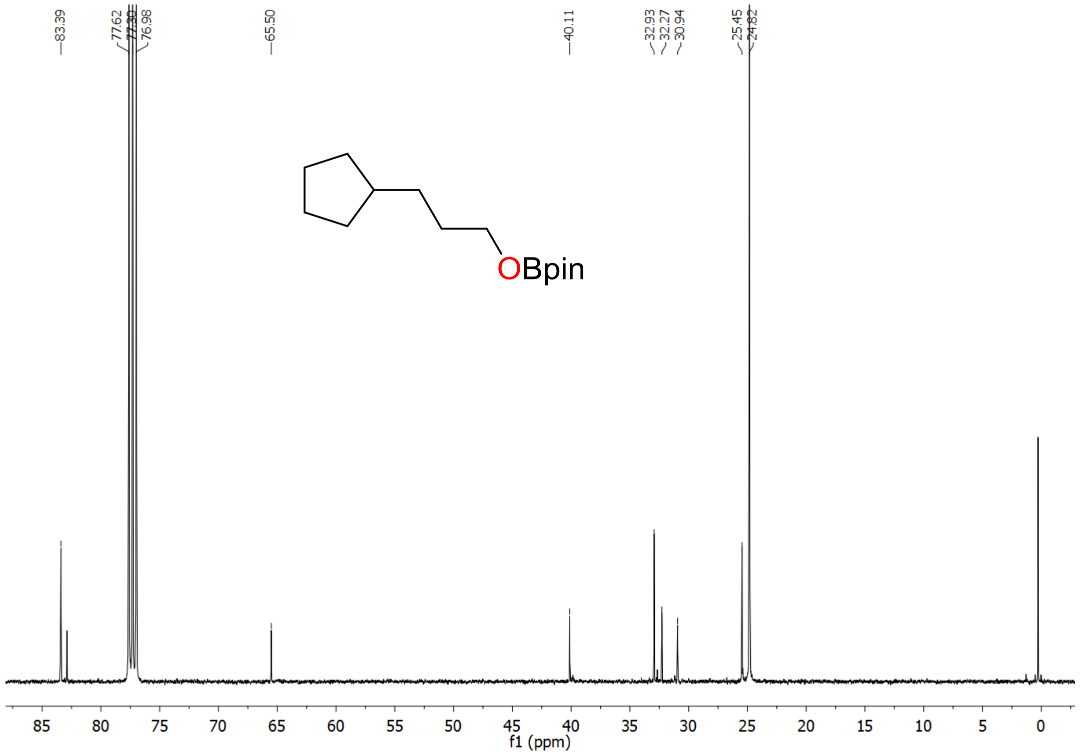

Supplementary Figure 37: $^{13}C\{^{1}H\}$-NMR (101 MHz, $CDCl_3$, 296K) of 4m.

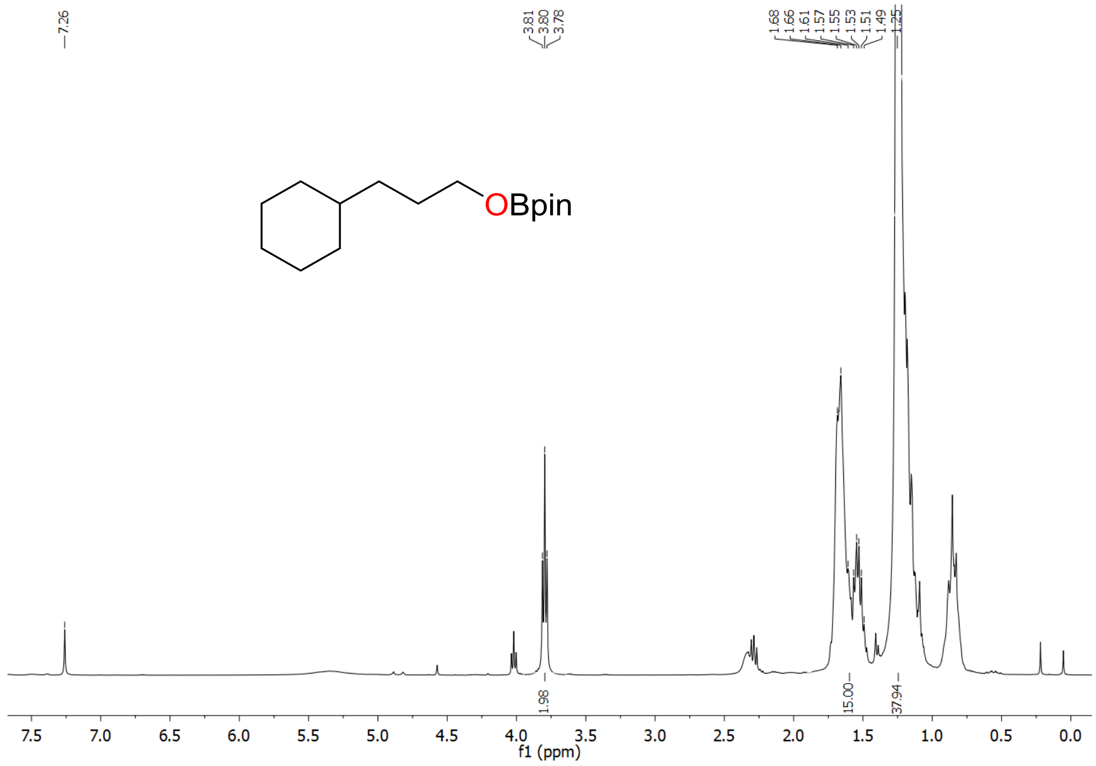

Supplementary Figure 38: ^{1}H-NMR (400 MHz, $CDCl_3$, 296K) of 4n.

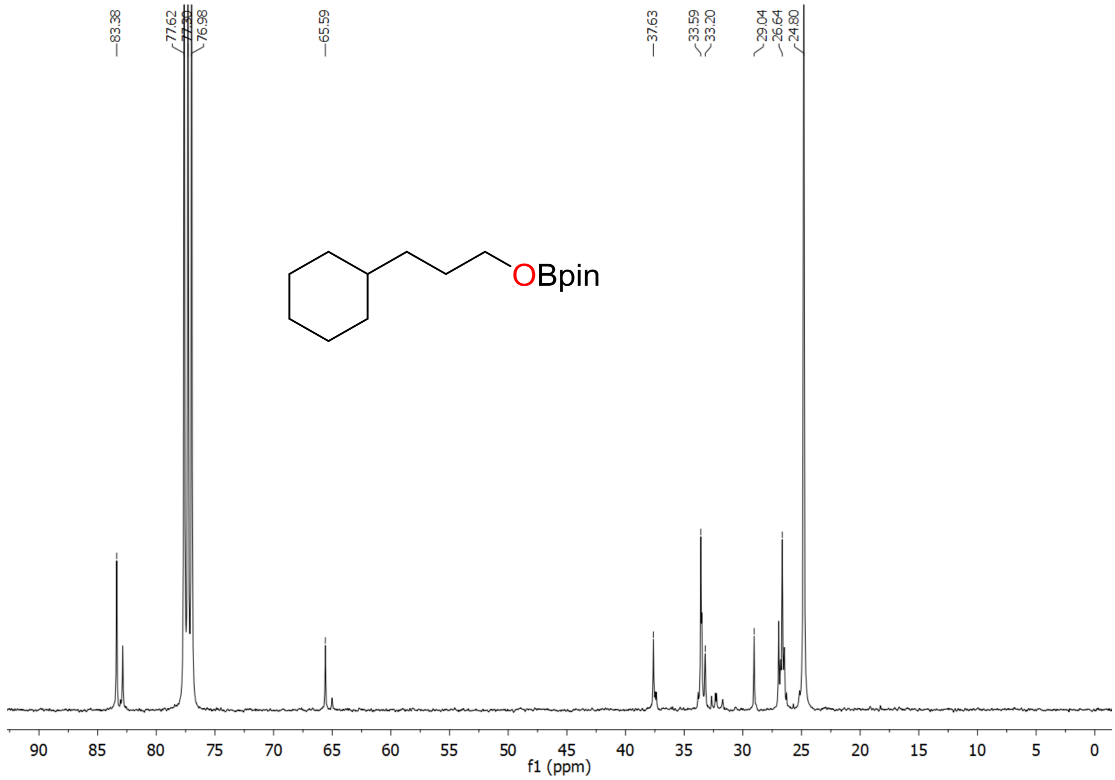

Supplementary Figure 39: $^{13}C\{^1H\}$-NMR (101 MHz, $CDCl_3$, 296K) of 4n.

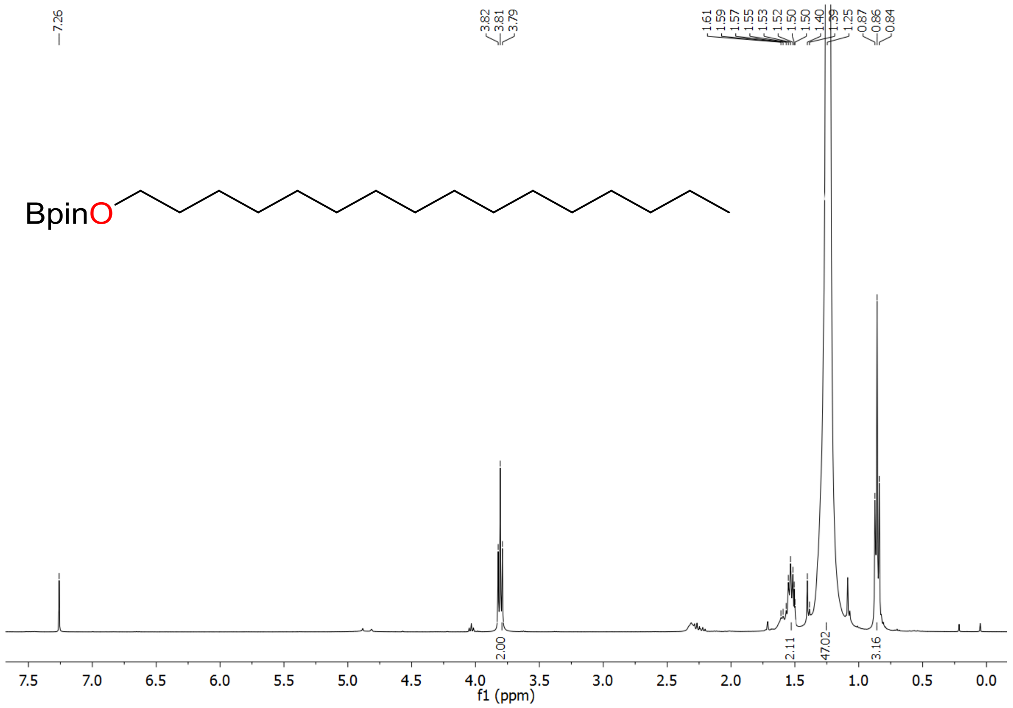

Supplementary Figure 40: ^{1}H-NMR (400 MHz, $CDCl_3$, 296K) of 4o.

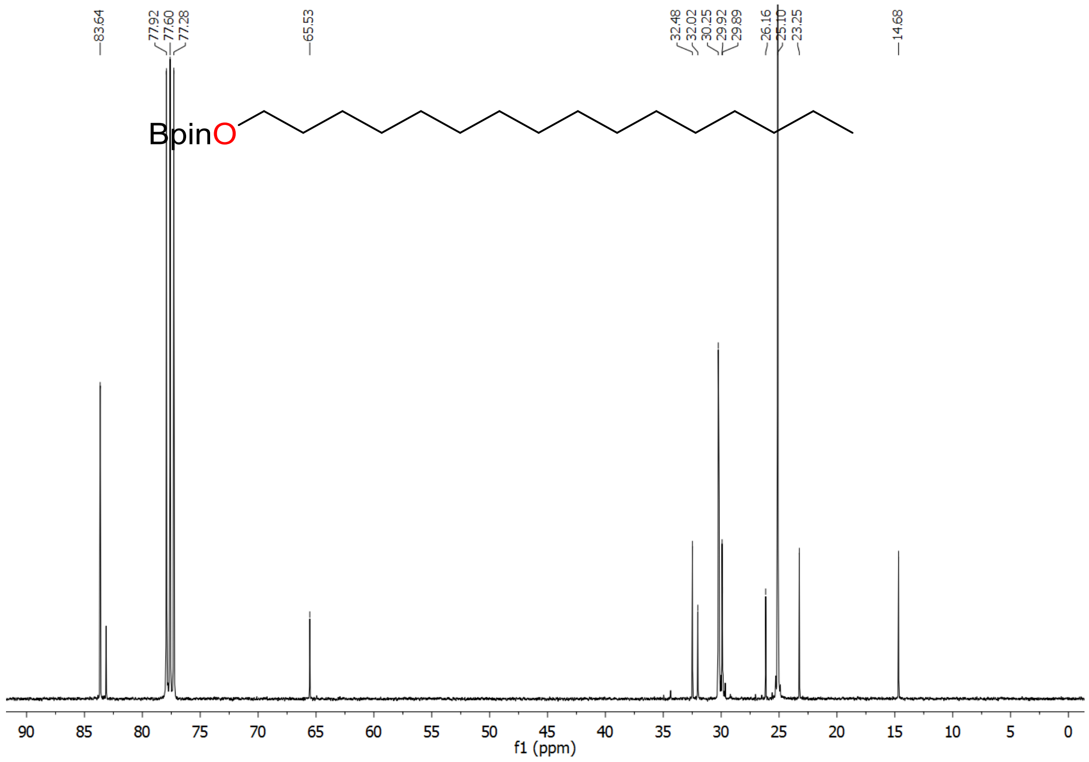

Supplementary Figure 41: $^{13}C\{^1H\}$-NMR (101 MHz, $CDCl_3$, 296K) of 4o.

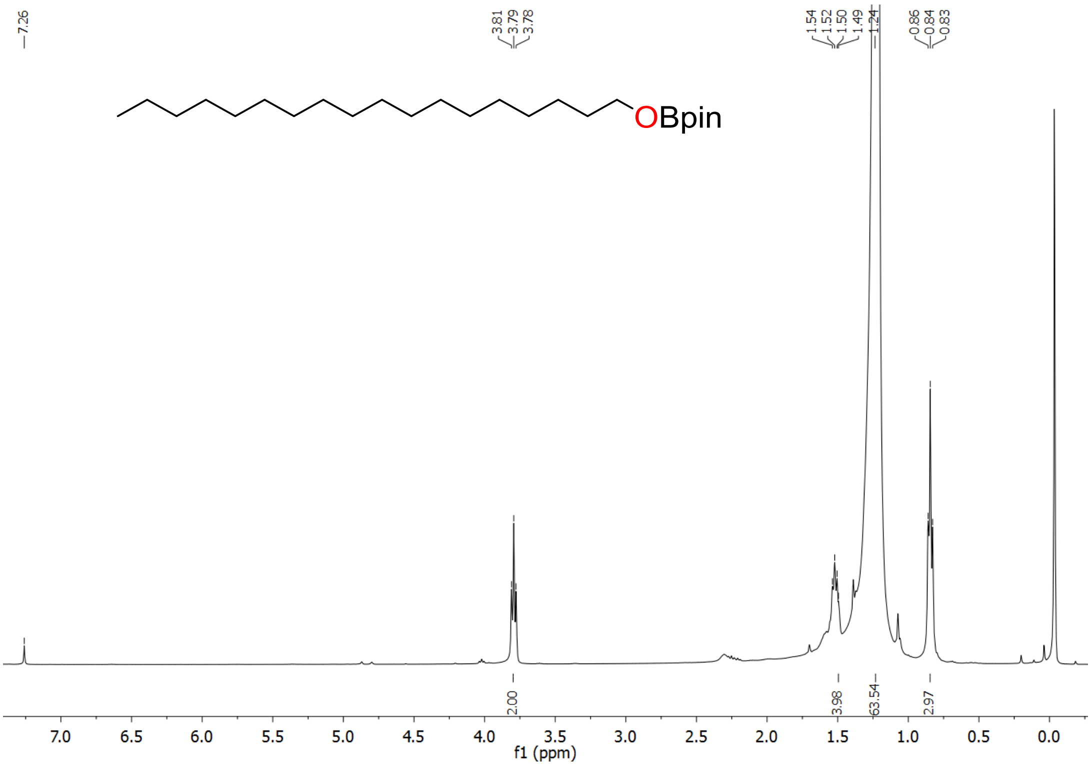

Supplementary Figure 42: ^{1}H-NMR (400 MHz, $CDCl_3$, 296K) of 4p.

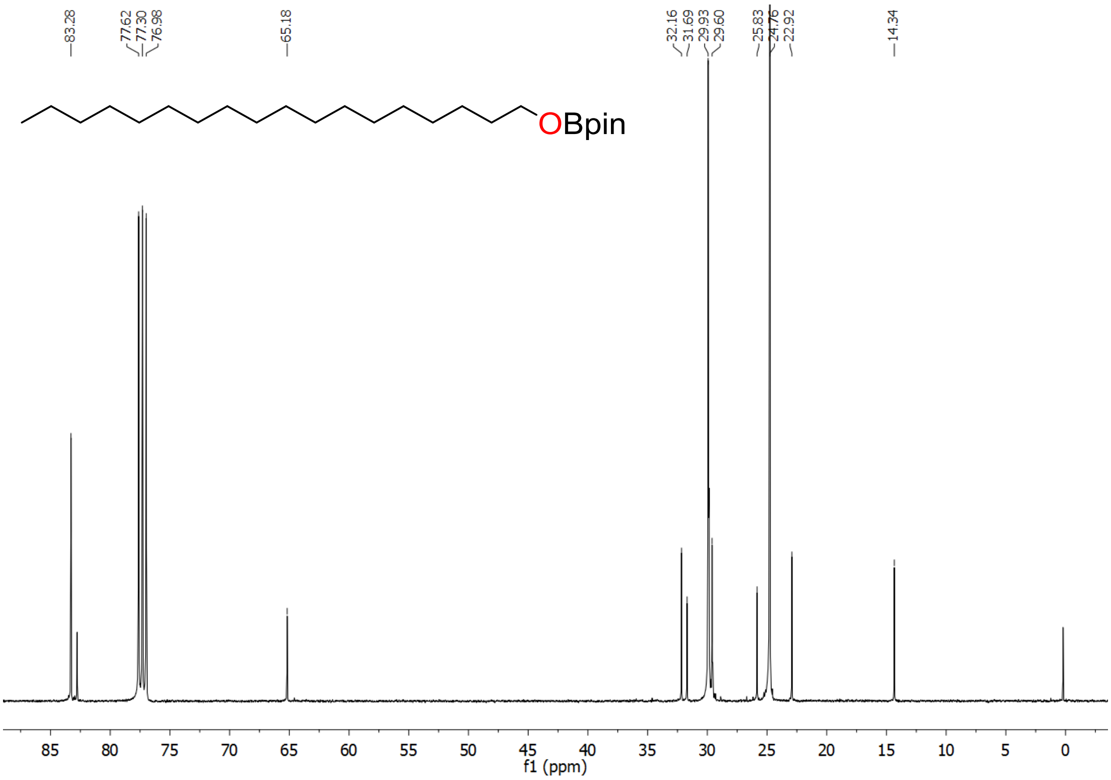

Supplementary Figure 43: $^{13}C\{^{1}H\}$-NMR (101 MHz, $CDCl_3$, 296K) of 4p.

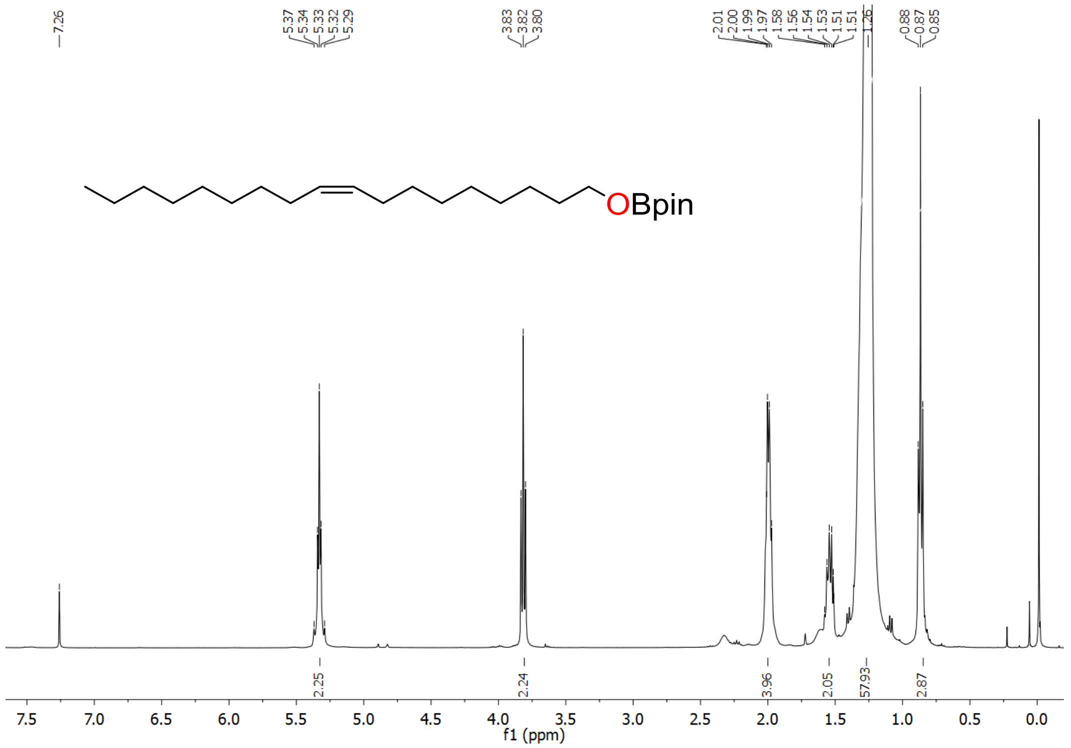

Supplementary Figure 44: ^{1}H-NMR (400 MHz, $CDCl_3$, 296K) of 4q.

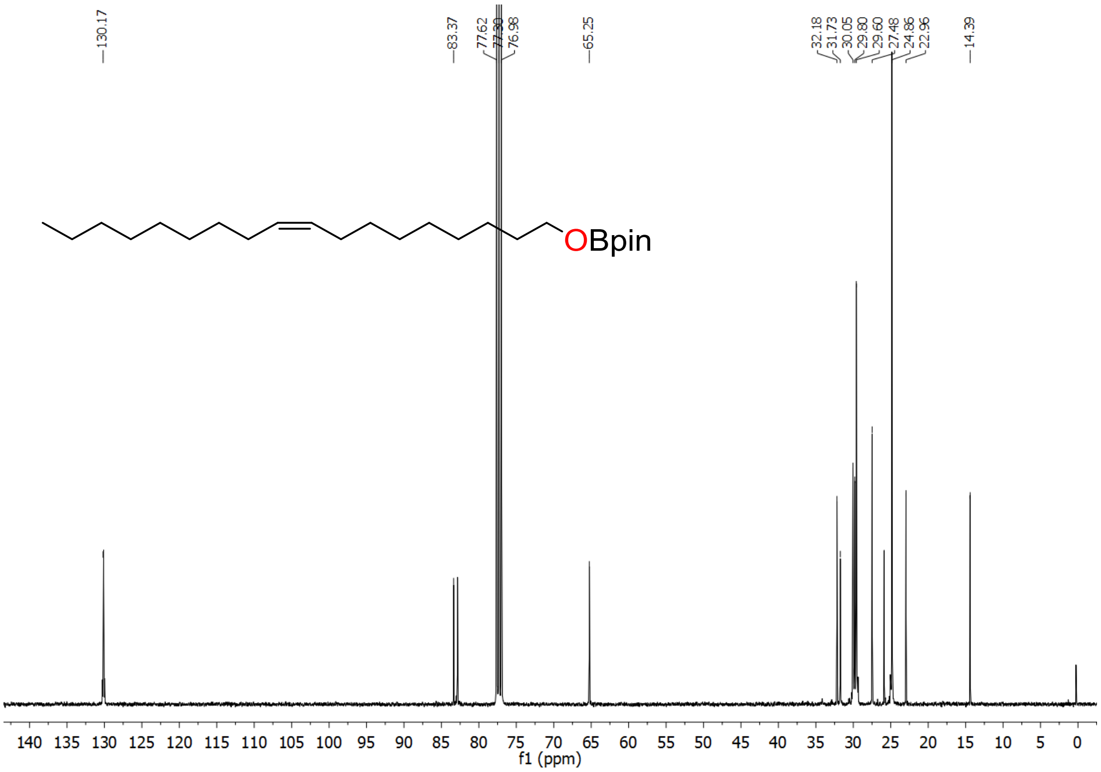

Supplementary Figure 45: $^{13}C\{^{1}H\}$-NMR (101 MHz, $CDCl_3$, 296K) of 4q.

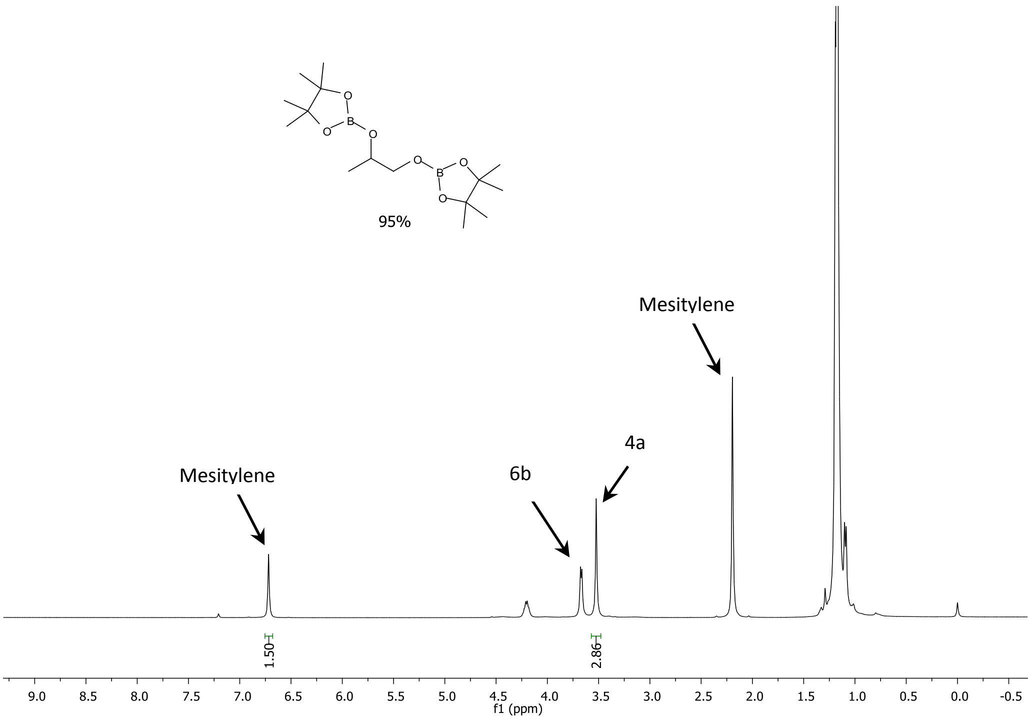

Supplementary Figure 46: ^{1}H-NMR (400 MHz, $CDCl_3$, 298 K) spectrum for hydroboration of propylene carbonate (Table 2, entry b).

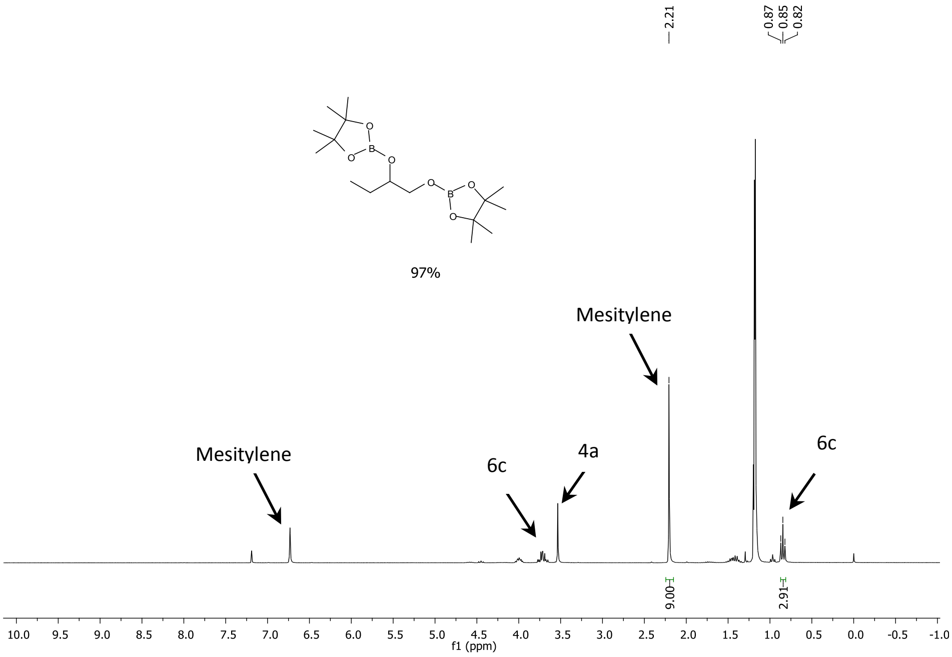

Supplementary Figure 47: ^{1}H-NMR (300 MHz, $CDCl_3$,298 K) spectrum for hydroboration of butylene carbonate (Table 2, entry c).

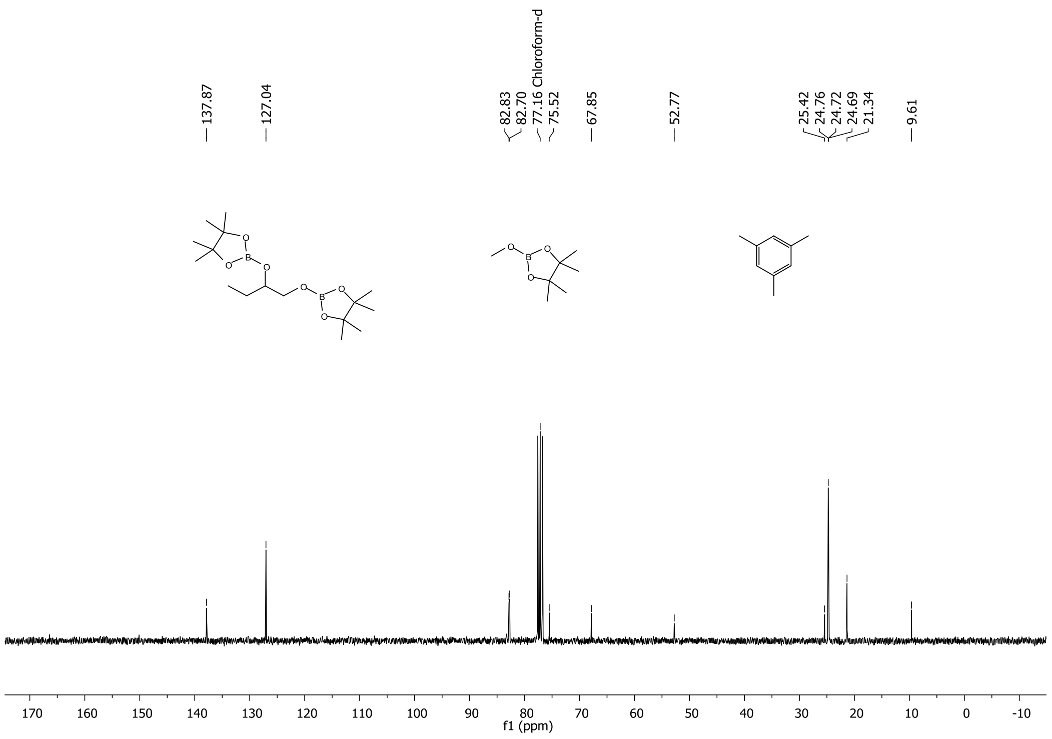

Supplementary Figure 48: $^{13}C\{^{1}H\}$-NMR (75 MHz, $CDCl_3$,298 K) spectrum for hydroboration of butylene carbonate (Table 2, entry c).

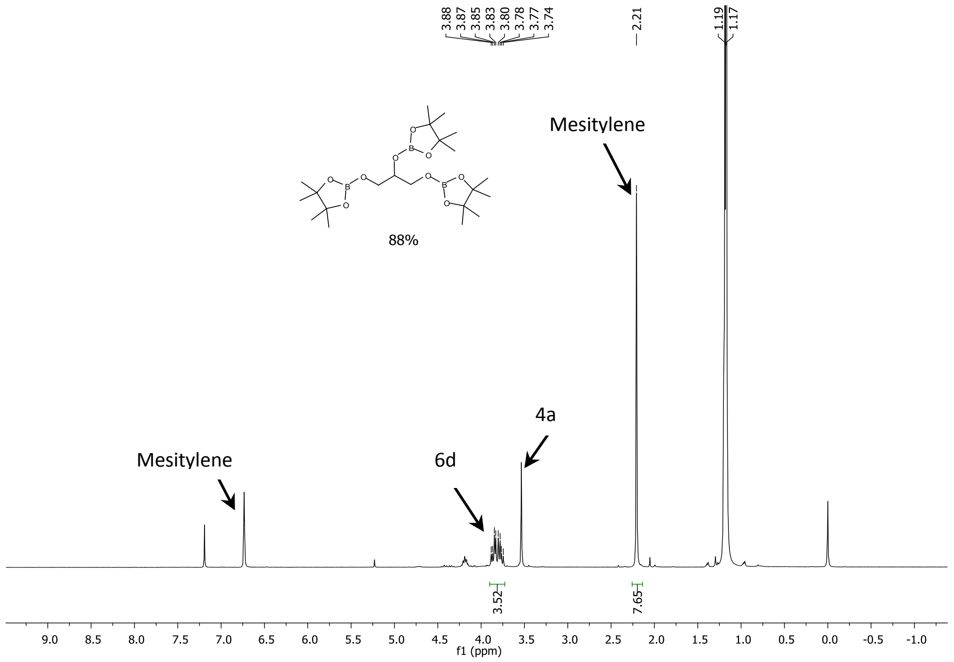

Supplementary Figure 49: ^{1}H-NMR (300 MHz, $CDCl_3$, 298 K) spectrum for hydroboration of glycerol 1-2 carbonate (Table 2, entry d).

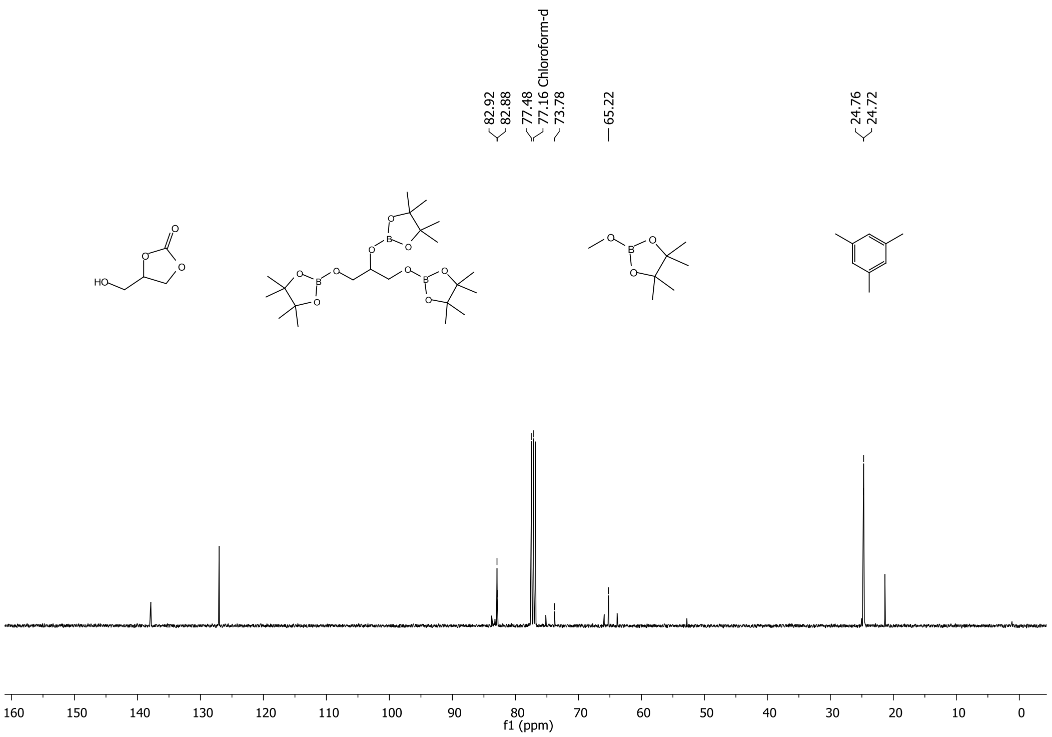

Supplementary Figure 50: $^{13}C\{^{1}H\}$-NMR (101 MHz, $CDCl_3$, 298 K) spectrum for hydroboration of glycerol 1-2 carbonate (Table 2, entry d).

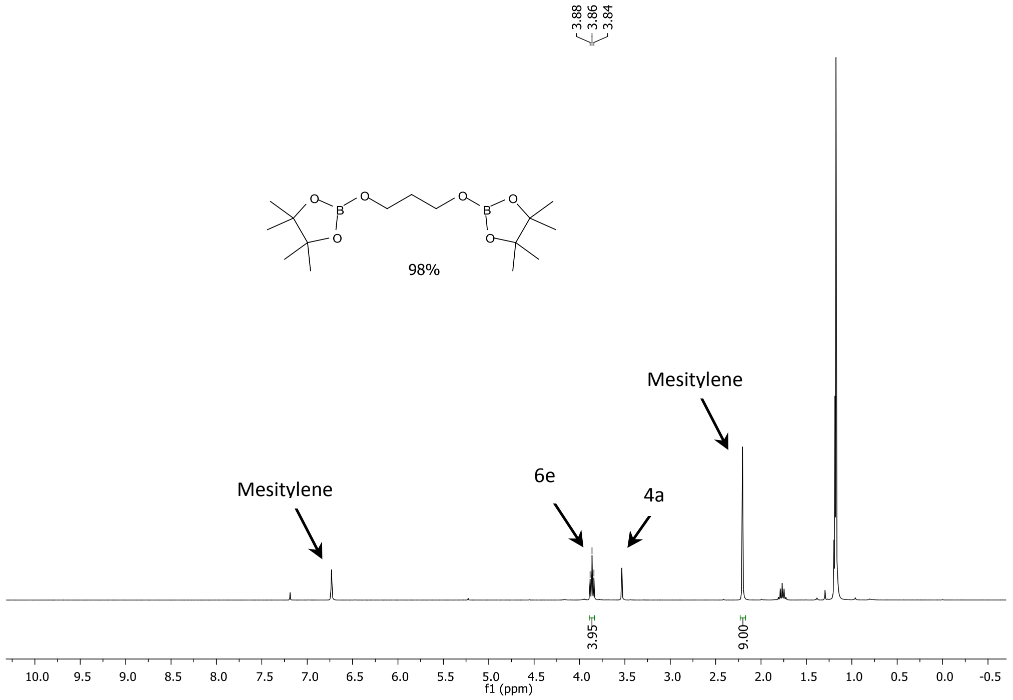

Supplementary Figure 51: ^{1}H-NMR (300 MHz, $CDCl_3$, 298 K) spectrum for hydroboration of 1,3-dioxan-2-one (Table 2, entry e).

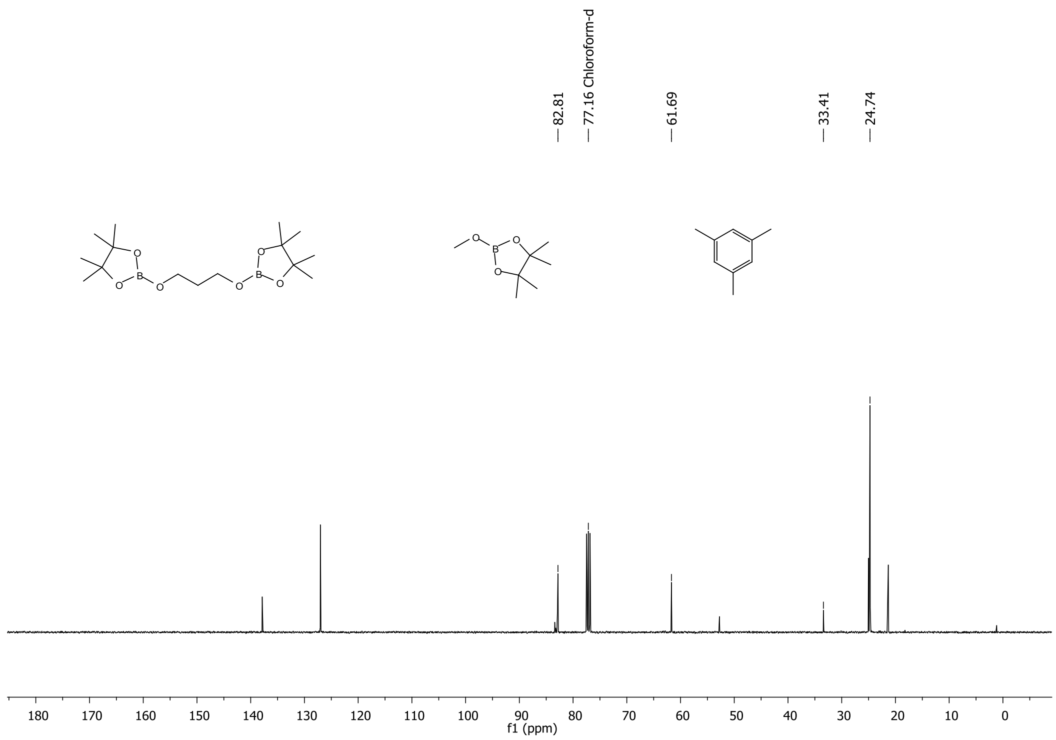

Supplementary Figure 52: $^{13}C\{^{1}H\}$-NMR (101 MHz, $CDCl_3$, 298 K) spectrum for hydroboration of 1,3-dioxan-2-one (Table 2, entry e).

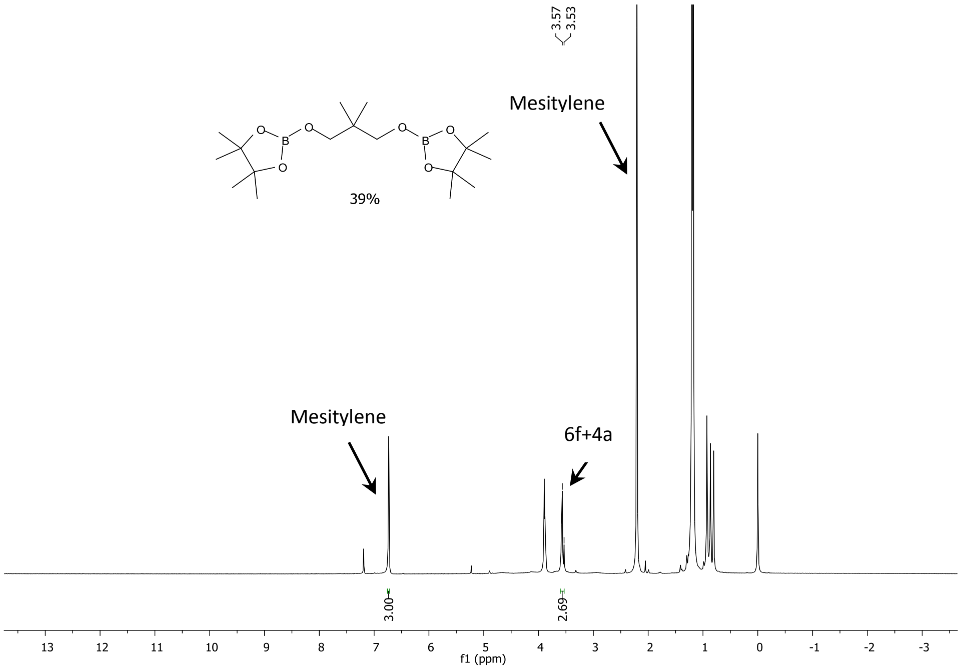

Supplementary Figure 53: ^{1}H-NMR (300 MHz, $CDCl_3$, 298 K) spectrum for hydroboration of 5,5-dimethyl-1,3-dioxan-2-one (Table 2, entry f).

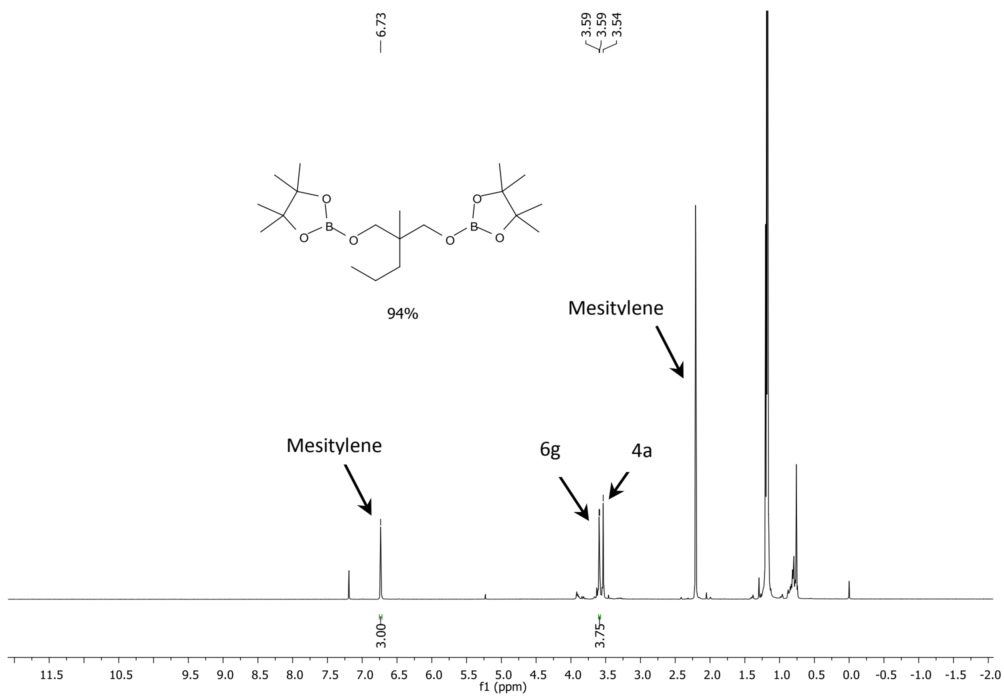

Supplementary Figure 54: ^{1}H-NMR (300 MHz, $CDCl_3$, 298 K) spectrum for hydroboration of 5-methyl-5-propyl-1,3-dioxan-2-one (Table 2, entry g).

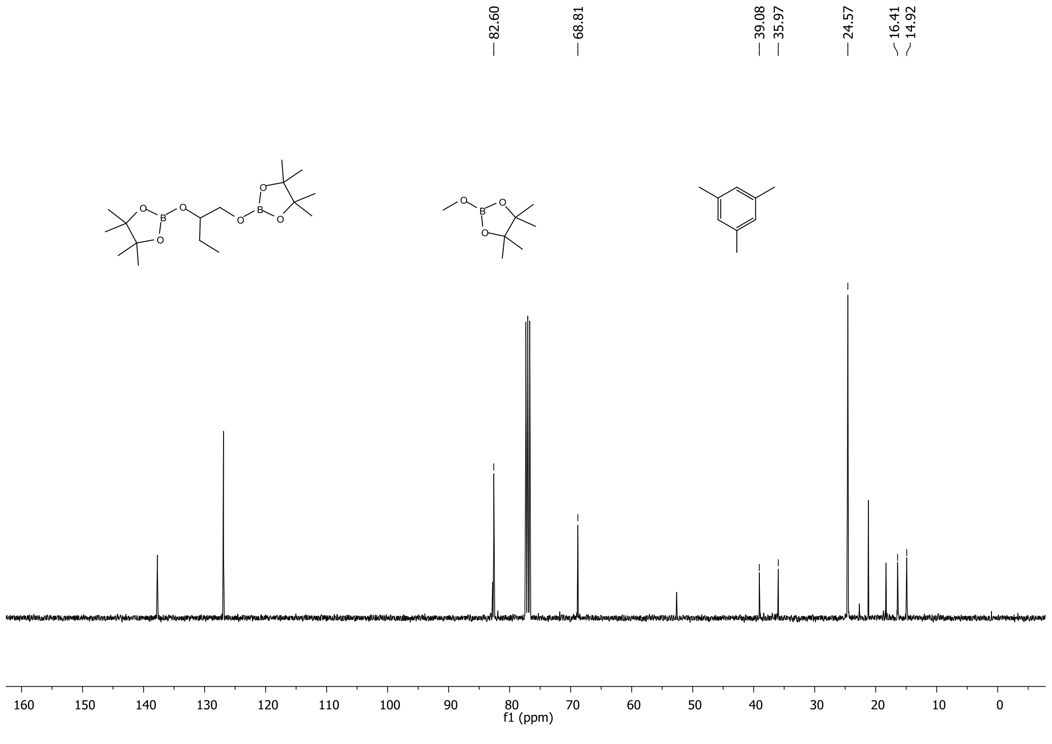

Supplementary Figure 55: $^{13}C\{^{1}H\}$-NMR (101 MHz, $CDCl_3$, 298 K) spectrum for hydroboration of 5-methyl-5-propyl-1,3-dioxan-2-one (Table 2, entry g).

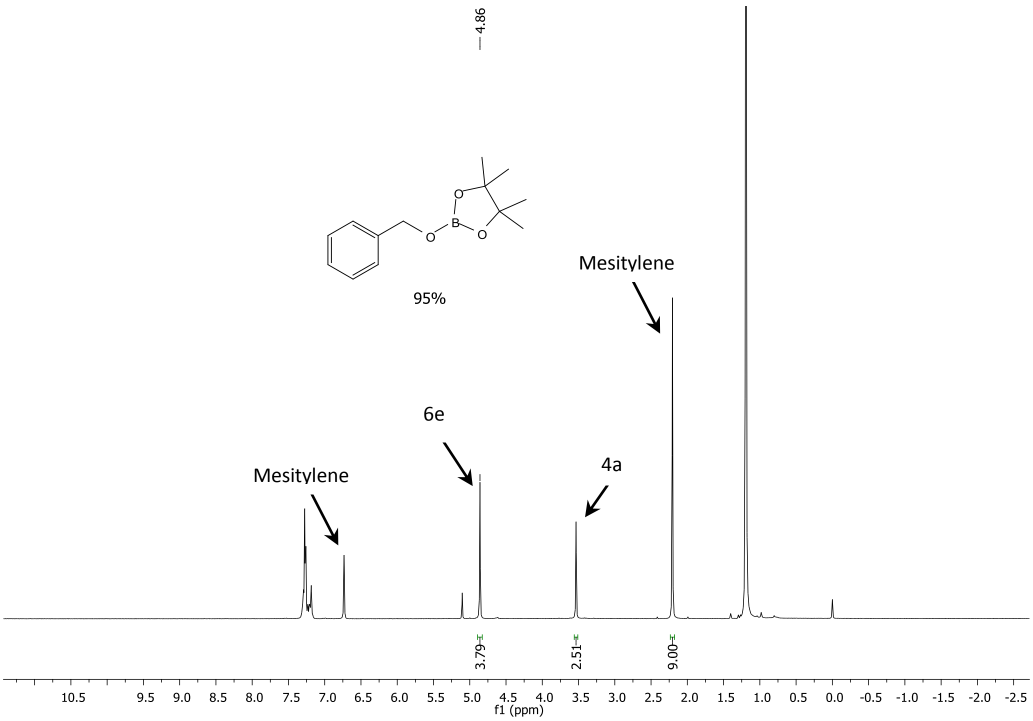

Supplementary Figure 56: ^{1}H-NMR (300 MHz, $CDCl_3$, 298 K) spectrum for hydroboration of dibenzyl carbonate (Table 2, entry h).

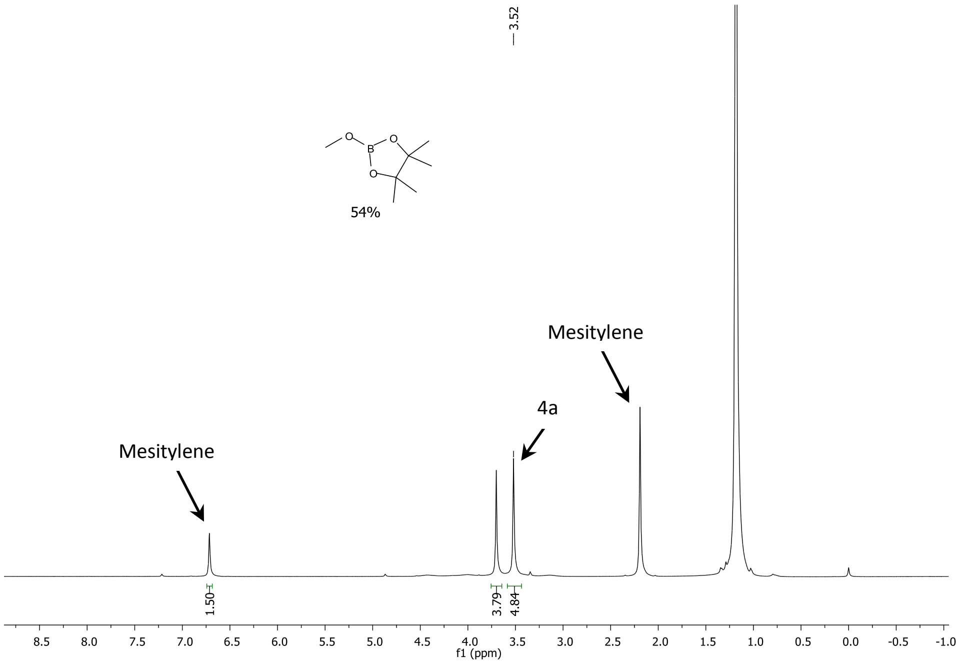

Supplementary Figure 57: ^{1}H-NMR (400 MHz, $CDCl_3$,298 K) spectrum for hydroboration of dimethyl carbonate (Table 2, entry j).

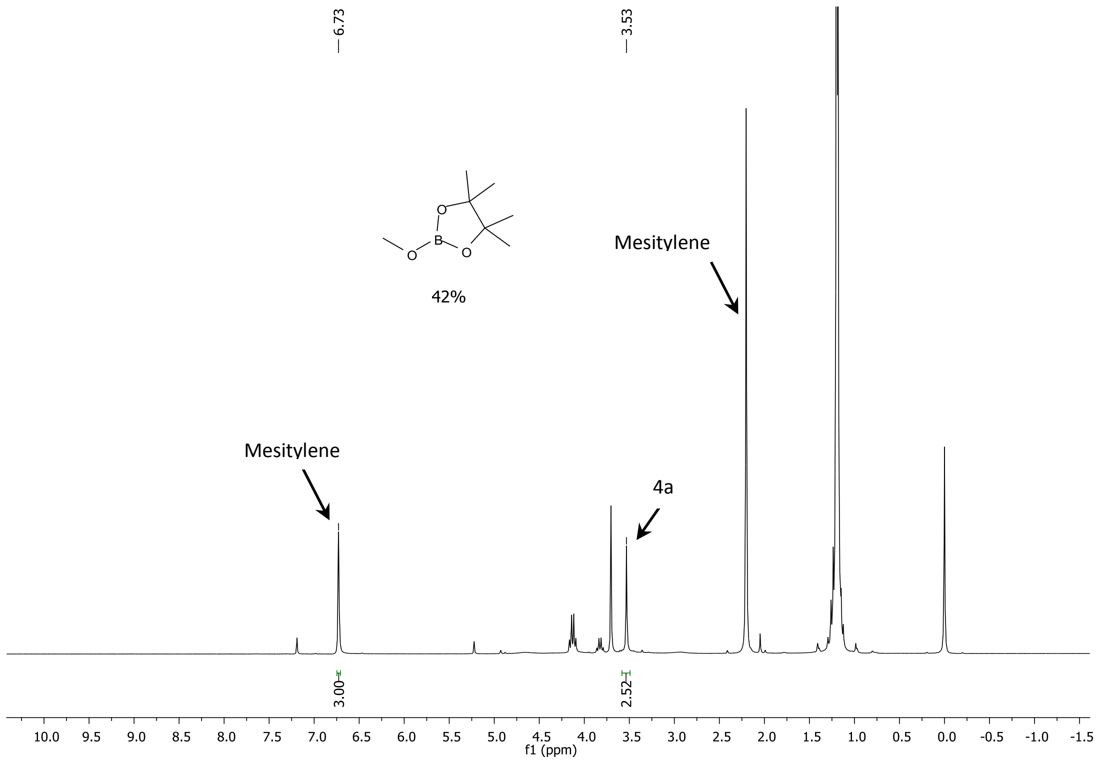

Supplementary Figure 58: ^{1}H-NMR (300 MHz, $CDCl_3$, 298 K) spectrum for hydroboration of ethyl methyl carbonate (Table 2, entry i).

Crystal data and structure refinement for (1)

Summary of Data CCDC 1868572

Identification code 1

Empirical formula C32 H37 Br Mn N O2 P2 Si2

Formula weight 720.59

Temperature 100(2) K

Wavelength 0.71073 Å

Crystal system Triclinic

Space group P -1

Unit cell dimensions a = 10.1969(15) Å α= 88.898(3)°.

b = 16.800(3) Å β= 80.928(3)°.

c = 21.342(3) Å γ = 88.327(3)°.

Volume 3608.4(10) $Å^3$

Z 4

Density (calculated) 1.326 Mg/m^3

Absorption coefficient 1.656 mm^{-1}

F(000) 1480

Crystal size 0.082 x 0.081 x 0.031 mm^3

Theta range for data collection 2.625 to 29.000°.

Index ranges -13<=h<=13, -22<=k<=22, -29<=l<=29

Reflections collected 89657

Independent reflections 19152 [R(int) = 0.0495]

Completeness to theta = 25.242° 99.8 %

Absorption correction	Gaussian
Max. and min. transmission	0.96156 and 0.89033
Refinement method	Full-matrix least-squares on F^2
Data / restraints / parameters	19152 / 139 / 756
Goodness-of-fit on F^2	1.039
Final R indices [I>2sigma(I)]	R1 = 0.0494, wR2 = 0.1172
R indices (all data)	R1 = 0.0671, wR2 = 0.1252
Extinction coefficient	n/a
Largest diff. peak and hole	1.775 and -0.950 e.$Å^{-3}$

Supplementary Table 5: Atomic coordinates (x 10^4) and equivalent isotropic displacement parameters ($Å^2$x 10^3) for 1.

U(eq) is defined as one third of the trace of the orthogonalized U^{ij} tensor.

	x	y	z	U(eq)
Mn(1)	-230(1)	6491(1)	2341(1)	11(1)
Br(1)	71(1)	7746(1)	2933(1)	20(1)
P(1)	58(1)	7250(1)	1423(1)	14(1)
C(2)	1781(4)	7534(6)	1253(7)	23(2)
Si(1)	2871(4)	6735(6)	1528(2)	23(1)
C(32)	4497(7)	7167(12)	1585(5)	55(3)
C(33)	3100(17)	5880(9)	977(5)	51(3)
C(2')	1834(8)	7410(20)	1220(30)	23(2)
Si(1')	2856(16)	6572(13)	1490(4)	23(1)
C(32')	4610(20)	6830(40)	1490(20)	55(3)
C(33')	2850(70)	5680(30)	1000(20)	51(3)
N(4)	2018(2)	6403(2)	2270(1)	16(1)
Si(2)	2498(1)	5588(1)	2728(1)	24(1)
C(6)	1306(3)	5692(2)	3478(2)	26(1)
P(7)	-371(1)	5835(1)	3305(1)	18(1)
C(8)	-356(3)	6757(2)	723(1)	21(1)
C(9)	564(4)	6553(3)	194(2)	36(1)

C(10)	167(5)	6156(3)	-308(2)	49(1)
C(11)	-1142(5)	5959(2)	-282(2)	45(1)
C(12)	-2065(5)	6175(2)	229(2)	43(1)
C(13)	-1682(4)	6563(2)	736(2)	29(1)
C(14)	-844(3)	8198(2)	1349(1)	19(1)
C(15)	-1906(4)	8441(2)	1784(2)	34(1)
C(16)	-2606(5)	9149(3)	1696(2)	44(1)
C(17)	-2241(4)	9618(2)	1178(2)	35(1)
C(18)	-1195(4)	9380(2)	729(2)	42(1)
C(19)	-505(4)	8669(2)	815(2)	39(1)
C(20)	-1051(8)	4825(3)	3340(5)	23(1)
C(21)	-2409(8)	4793(4)	3332(8)	27(1)
C(22)	-3025(7)	4062(6)	3374(8)	40(1)
C(23)	-2283(9)	3364(4)	3425(6)	61(3)
C(24)	-925(9)	3395(3)	3433(3)	52(3)
C(25)	-309(6)	4126(4)	3390(3)	33(2)
C(20')	-1204(16)	4882(5)	3413(10)	23(1)
C(21')	-2478(18)	4799(8)	3272(15)	27(1)
C(22')	-3085(15)	4067(11)	3360(15)	40(1)
C(23')	-2419(19)	3417(7)	3589(12)	61(3)
C(24')	-1145(18)	3500(6)	3730(7)	52(3)
C(25')	-538(12)	4233(8)	3642(6)	33(2)
C(26)	-1310(3)	6292(2)	4020(2)	26(1)

C(27)	-932(4)	6172(3)	4617(2)	42(1)
C(28)	-1714(5)	6509(3)	5154(2)	54(1)
C(29)	-2847(5)	6944(3)	5107(2)	53(1)
C(30)	-3216(4)	7069(3)	4522(2)	48(1)
C(31)	-2446(4)	6748(2)	3982(2)	33(1)
C(34)	4207(3)	5662(3)	2908(2)	41(1)
C(35)	2393(4)	4635(2)	2318(3)	46(1)
C(36)	-333(3)	5605(2)	1923(2)	19(1)
O(37)	-408(3)	5030(1)	1661(1)	28(1)
C(38)	-1961(3)	6568(2)	2395(1)	16(1)
O(39)	-3110(2)	6591(2)	2412(1)	27(1)
Mn(2)	3845(1)	1457(1)	2366(1)	13(1)
Br(2)	3411(1)	2681(1)	3070(1)	24(1)
P(51)	4057(1)	2278(1)	1487(1)	17(1)
C(52)	2440(4)	2619(3)	1325(2)	19(1)
Si(3)	1156(1)	1882(1)	1644(1)	16(1)
N(54)	1623(5)	1535(3)	2368(2)	13(1)
C(82)	1102(10)	1049(5)	1089(4)	41(2)
C(83)	-462(6)	2437(6)	1752(4)	47(3)
C(52')	2412(6)	2738(5)	1492(6)	19(1)
Si(3')	1011(3)	2074(2)	1801(2)	16(1)
N(54')	1565(9)	1640(5)	2478(3)	13(1)
C(82')	800(20)	1284(10)	1229(9)	41(2)

C(83')	-576(12)	2642(12)	2023(8)	47(3)
Si(4)	759(1)	811(1)	2889(1)	21(1)
C(56)	1710(3)	723(2)	3569(2)	19(1)
P(57)	3490(1)	720(1)	3290(1)	14(1)
C(58)	4814(4)	1792(2)	747(2)	31(1)
C(59)	4134(5)	1620(3)	255(2)	57(1)
C(60)	4835(6)	1196(3)	-288(2)	58(1)
C(61)	6138(6)	980(3)	-311(2)	57(1)
C(62)	6805(6)	1179(3)	157(2)	52(1)
C(63)	6156(5)	1572(2)	693(2)	39(1)
C(64)	5070(3)	3165(2)	1416(1)	17(1)
C(65)	5915(3)	3332(2)	1847(2)	23(1)
C(66)	6691(3)	4002(2)	1761(2)	30(1)
C(67)	6637(3)	4513(2)	1254(2)	27(1)
C(68)	5806(3)	4352(2)	820(2)	24(1)
C(69)	5034(3)	3680(2)	899(2)	21(1)
C(70)	4024(3)	-330(2)	3260(1)	17(1)
C(71)	3149(3)	-954(2)	3280(2)	24(1)
C(72)	3617(4)	-1739(2)	3270(2)	32(1)
C(73)	4958(4)	-1908(2)	3240(2)	29(1)
C(74)	5839(3)	-1297(2)	3222(2)	25(1)
C(75)	5377(3)	-511(2)	3226(1)	21(1)
C(76)	4224(3)	1003(2)	3979(1)	17(1)

C(77)	3668(3)	733(2)	4581(2)	26(1)
C(78)	4270(4)	882(3)	5106(2)	35(1)
C(79)	5442(4)	1287(2)	5035(2)	31(1)
C(80)	5999(3)	1551(2)	4443(2)	30(1)
C(81)	5394(3)	1421(2)	3914(2)	24(1)
C(84)	735(4)	-106(2)	2432(2)	33(1)
C(85)	-972(3)	1123(2)	3217(2)	36(1)
C(86)	3984(3)	584(2)	1903(2)	21(1)
O(87)	4087(3)	32(1)	1618(1)	36(1)
C(88)	5580(3)	1369(2)	2314(1)	19(1)
O(89)	6723(2)	1267(2)	2246(1)	29(1)

Supplementary Table 6: Bond lengths [Å] and angles [°] for 1.

Mn(1)-C(38)	1.752(3)
Mn(1)-C(36)	1.765(3)
Mn(1)-N(4)	2.274(2)
Mn(1)-P(1)	2.2997(9)
Mn(1)-P(7)	2.3022(9)
Mn(1)-Br(1)	2.5290(6)
P(1)-C(2)	1.813(4)
P(1)-C(2')	1.822(8)
P(1)-C(14)	1.833(3)
P(1)-C(8)	1.834(3)
C(2)-Si(1)	1.863(3)
Si(1)-N(4)	1.770(3)
Si(1)-C(32)	1.851(4)
Si(1)-C(33)	1.860(4)
C(2')-Si(1')	1.865(5)
Si(1')-N(4)	1.768(4)
Si(1')-C(32')	1.854(5)
Si(1')-C(33')	1.857(5)
N(4)-Si(2)	1.771(2)
Si(2)-C(34)	1.851(4)
Si(2)-C(35)	1.855(4)

Si(2)-C(6)	1.858(4)
C(6)-P(7)	1.814(3)
P(7)-C(20')	1.828(7)
P(7)-C(26)	1.836(4)
P(7)-C(20)	1.848(4)
C(8)-C(9)	1.391(5)
C(8)-C(13)	1.396(5)
C(9)-C(10)	1.391(5)
C(10)-C(11)	1.377(7)
C(11)-C(12)	1.370(7)
C(12)-C(13)	1.387(5)
C(14)-C(15)	1.369(5)
C(14)-C(19)	1.379(5)
C(15)-C(16)	1.394(5)
C(16)-C(17)	1.356(5)
C(17)-C(18)	1.371(6)
C(18)-C(19)	1.392(5)
C(20)-C(21)	1.3900
C(20)-C(25)	1.3900
C(21)-C(22)	1.3900
C(22)-C(23)	1.3900
C(23)-C(24)	1.3900
C(24)-C(25)	1.3900

C(20')-C(21')	1.3900
C(20')-C(25')	1.3900
C(21')-C(22')	1.3900
C(22')-C(23')	1.3900
C(23')-C(24')	1.3900
C(24')-C(25')	1.3900
C(26)-C(31)	1.382(5)
C(26)-C(27)	1.398(5)
C(27)-C(28)	1.409(6)
C(28)-C(29)	1.364(7)
C(29)-C(30)	1.371(6)
C(30)-C(31)	1.397(5)
C(36)-O(37)	1.135(4)
C(38)-O(39)	1.166(4)
Mn(2)-C(88)	1.757(3)
Mn(2)-C(86)	1.774(3)
Mn(2)-N(54)	2.265(5)
Mn(2)-P(51)	2.2920(9)
Mn(2)-P(57)	2.2932(9)
Mn(2)-N(54')	2.311(9)
Mn(2)-Br(2)	2.5572(6)
P(51)-C(52)	1.812(4)
P(51)-C(52')	1.826(7)

P(51)-C(64)	1.829(3)
P(51)-C(58)	1.841(4)
C(52)-Si(3)	1.869(3)
Si(3)-N(54)	1.771(3)
Si(3)-C(83)	1.855(4)
Si(3)-C(82)	1.858(4)
N(54)-Si(4)	1.786(5)
C(52')-Si(3')	1.867(4)
Si(3')-N(54')	1.770(4)
Si(3')-C(83')	1.855(4)
Si(3')-C(82')	1.859(4)
N(54')-Si(4)	1.782(9)
Si(4)-C(84)	1.842(4)
Si(4)-C(85)	1.857(4)
Si(4)-C(56)	1.870(3)
C(56)-P(57)	1.818(3)
P(57)-C(76)	1.828(3)
P(57)-C(70)	1.831(3)
C(58)-C(59)	1.385(5)
C(58)-C(63)	1.394(6)
C(59)-C(60)	1.450(7)
C(60)-C(61)	1.361(8)
C(61)-C(62)	1.345(7)

C(62)-C(63)	1.396(5)
C(64)-C(69)	1.392(4)
C(64)-C(65)	1.393(4)
C(65)-C(66)	1.387(4)
C(66)-C(67)	1.375(5)
C(67)-C(68)	1.386(5)
C(68)-C(69)	1.387(4)
C(70)-C(71)	1.392(4)
C(70)-C(75)	1.395(4)
C(71)-C(72)	1.389(5)
C(72)-C(73)	1.381(5)
C(73)-C(74)	1.380(5)
C(74)-C(75)	1.388(4)
C(76)-C(81)	1.389(4)
C(76)-C(77)	1.393(4)
C(77)-C(78)	1.390(5)
C(78)-C(79)	1.379(5)
C(79)-C(80)	1.372(5)
C(80)-C(81)	1.393(5)
C(86)-O(87)	1.112(4)
C(88)-O(89)	1.160(4)

C(38)-Mn(1)-C(36)	86.14(14)

C(38)-Mn(1)-N(4)	179.51(12)
C(36)-Mn(1)-N(4)	94.25(11)
C(38)-Mn(1)-P(1)	91.36(10)
C(36)-Mn(1)-P(1)	92.47(10)
N(4)-Mn(1)-P(1)	88.32(6)
C(38)-Mn(1)-P(7)	92.37(10)
C(36)-Mn(1)-P(7)	93.12(10)
N(4)-Mn(1)-P(7)	87.91(6)
P(1)-Mn(1)-P(7)	173.47(3)
C(38)-Mn(1)-Br(1)	97.40(10)
C(36)-Mn(1)-Br(1)	176.45(10)
N(4)-Mn(1)-Br(1)	82.21(7)
P(1)-Mn(1)-Br(1)	87.60(3)
P(7)-Mn(1)-Br(1)	86.61(3)
C(2)-P(1)-C(14)	102.7(3)
C(2')-P(1)-C(14)	109.0(9)
C(2)-P(1)-C(8)	108.3(5)
C(2')-P(1)-C(8)	103(2)
C(14)-P(1)-C(8)	98.70(13)
C(2)-P(1)-Mn(1)	108.4(3)
C(2')-P(1)-Mn(1)	106.7(12)
C(14)-P(1)-Mn(1)	122.46(10)
C(8)-P(1)-Mn(1)	114.96(11)

P(1)-C(2)-Si(1) 110.1(3)

N(4)-Si(1)-C(32) 113.1(4)

N(4)-Si(1)-C(33) 108.4(4)

C(32)-Si(1)-C(33) 110.1(5)

N(4)-Si(1)-C(2) 105.4(4)

C(32)-Si(1)-C(2) 108.2(3)

C(33)-Si(1)-C(2) 111.6(7)

P(1)-C(2')-Si(1') 112.5(11)

N(4)-Si(1')-C(32') 111.4(16)

N(4)-Si(1')-C(33') 111.2(19)

C(32')-Si(1')-C(33') 108(3)

N(4)-Si(1')-C(2') 101.4(18)

C(32')-Si(1')-C(2') 113.5(16)

C(33')-Si(1')-C(2') 112(3)

Si(1')-N(4)-Si(2) 120.2(7)

Si(1)-N(4)-Si(2) 126.2(3)

Si(1')-N(4)-Mn(1) 113.0(6)

Si(1)-N(4)-Mn(1) 113.37(17)

Si(2)-N(4)-Mn(1) 110.75(11)

N(4)-Si(2)-C(34) 113.04(15)

N(4)-Si(2)-C(35) 110.92(18)

C(34)-Si(2)-C(35) 108.1(2)

N(4)-Si(2)-C(6) 101.97(13)

C(34)-Si(2)-C(6)	108.80(18)
C(35)-Si(2)-C(6)	114.05(19)
P(7)-C(6)-Si(2)	110.10(17)
C(6)-P(7)-C(20')	108.5(6)
C(6)-P(7)-C(26)	104.96(16)
C(20')-P(7)-C(26)	95.5(6)
C(6)-P(7)-C(20)	104.9(3)
C(26)-P(7)-C(20)	102.3(3)
C(6)-P(7)-Mn(1)	107.48(11)
C(20')-P(7)-Mn(1)	119.1(7)
C(26)-P(7)-Mn(1)	119.96(12)
C(20)-P(7)-Mn(1)	115.9(3)
C(9)-C(8)-C(13)	118.7(3)
C(9)-C(8)-P(1)	124.3(3)
C(13)-C(8)-P(1)	117.0(2)
C(10)-C(9)-C(8)	120.4(4)
C(11)-C(10)-C(9)	120.1(4)
C(12)-C(11)-C(10)	120.2(4)
C(11)-C(12)-C(13)	120.4(4)
C(12)-C(13)-C(8)	120.3(4)
C(15)-C(14)-C(19)	117.8(3)
C(15)-C(14)-P(1)	122.7(3)
C(19)-C(14)-P(1)	119.3(3)

C(14)-C(15)-C(16)	121.1(3)
C(17)-C(16)-C(15)	120.6(4)
C(16)-C(17)-C(18)	119.4(3)
C(17)-C(18)-C(19)	120.0(4)
C(14)-C(19)-C(18)	121.2(3)
C(21)-C(20)-C(25)	120.0
C(21)-C(20)-P(7)	115.5(5)
C(25)-C(20)-P(7)	124.5(5)
C(22)-C(21)-C(20)	120.0
C(21)-C(22)-C(23)	120.0
C(22)-C(23)-C(24)	120.0
C(25)-C(24)-C(23)	120.0
C(24)-C(25)-C(20)	120.0
C(21')-C(20')-C(25')	120.0
C(21')-C(20')-P(7)	121.4(10)
C(25')-C(20')-P(7)	118.6(10)
C(20')-C(21')-C(22')	120.0
C(23')-C(22')-C(21')	120.0
C(22')-C(23')-C(24')	120.0
C(25')-C(24')-C(23')	120.0
C(24')-C(25')-C(20')	120.0
C(31)-C(26)-C(27)	118.0(3)
C(31)-C(26)-P(7)	120.3(3)

C(27)-C(26)-P(7)	121.6(3)
C(26)-C(27)-C(28)	119.5(4)
C(29)-C(28)-C(27)	121.4(4)
C(28)-C(29)-C(30)	119.3(4)
C(29)-C(30)-C(31)	120.3(4)
C(26)-C(31)-C(30)	121.5(4)
O(37)-C(36)-Mn(1)	179.2(3)
O(39)-C(38)-Mn(1)	176.9(3)
C(88)-Mn(2)-C(86)	85.81(14)
C(88)-Mn(2)-N(54)	176.22(14)
C(86)-Mn(2)-N(54)	91.01(15)
C(88)-Mn(2)-P(51)	90.94(10)
C(86)-Mn(2)-P(51)	92.67(10)
N(54)-Mn(2)-P(51)	87.17(9)
C(88)-Mn(2)-P(57)	92.65(10)
C(86)-Mn(2)-P(57)	91.54(10)
N(54)-Mn(2)-P(57)	89.47(9)
P(51)-Mn(2)-P(57)	174.66(3)
C(88)-Mn(2)-N(54')	176.3(2)
C(86)-Mn(2)-N(54')	97.9(2)
P(51)-Mn(2)-N(54')	89.23(15)
P(57)-Mn(2)-N(54')	86.93(14)
C(88)-Mn(2)-Br(2)	99.28(10)

C(86)-Mn(2)-Br(2)	174.49(11)
N(54)-Mn(2)-Br(2)	83.98(11)
P(51)-Mn(2)-Br(2)	89.39(3)
P(57)-Mn(2)-Br(2)	86.14(3)
N(54')-Mn(2)-Br(2)	77.0(2)
C(52)-P(51)-C(64)	105.38(17)
C(52')-P(51)-C(64)	100.2(2)
C(52)-P(51)-C(58)	103.5(2)
C(52')-P(51)-C(58)	116.3(5)
C(64)-P(51)-C(58)	98.32(14)
C(52)-P(51)-Mn(2)	110.69(14)
C(52')-P(51)-Mn(2)	105.1(3)
C(64)-P(51)-Mn(2)	122.45(10)
C(58)-P(51)-Mn(2)	114.31(12)
P(51)-C(52)-Si(3)	110.1(2)
N(54)-Si(3)-C(83)	113.1(3)
N(54)-Si(3)-C(82)	111.2(4)
C(83)-Si(3)-C(82)	109.4(5)
N(54)-Si(3)-C(52)	104.8(2)
C(83)-Si(3)-C(52)	106.3(4)
C(82)-Si(3)-C(52)	112.0(4)
Si(3)-N(54)-Si(4)125.2(3)	
Si(3)-N(54)-Mn(2)	114.4(2)

Si(4)-N(54)-Mn(2)	112.14(16)
P(51)-C(52')-Si(3')	114.2(4)
N(54')-Si(3')-C(83')	111.1(6)
N(54')-Si(3')-C(82')	109.8(8)
C(83')-Si(3')-C(82')	110.0(10)
N(54')-Si(3')-C(52')	101.6(5)
C(83')-Si(3')-C(52')	112.0(8)
C(82')-Si(3')-C(52')	112.2(9)
Si(3')-N(54')-Si(4)	121.5(5)
Si(3')-N(54')-Mn(2)	113.6(4)
Si(4)-N(54')-Mn(2)	110.3(3)
N(54')-Si(4)-C(84)	116.7(3)
N(54)-Si(4)-C(84)	107.23(18)
N(54')-Si(4)-C(85)	108.5(3)
N(54)-Si(4)-C(85)	113.71(18)
C(84)-Si(4)-C(85)	109.30(19)
N(54')-Si(4)-C(56)	99.5(2)
N(54)-Si(4)-C(56)	104.53(16)
C(84)-Si(4)-C(56)	114.82(16)
C(85)-Si(4)-C(56)	107.35(16)
P(57)-C(56)-Si(4)	110.96(16)
C(56)-P(57)-C(76)	104.86(14)
C(56)-P(57)-C(70)	105.55(14)

C(76)-P(57)-C(70)	98.39(13)
C(56)-P(57)-Mn(2)	107.85(10)
C(76)-P(57)-Mn(2)	120.70(11)
C(70)-P(57)-Mn(2)	117.90(10)
C(59)-C(58)-C(63)	118.8(4)
C(59)-C(58)-P(51)	124.4(4)
C(63)-C(58)-P(51)	116.8(3)
C(58)-C(59)-C(60)	118.7(5)
C(61)-C(60)-C(59)	120.1(4)
C(62)-C(61)-C(60)	120.7(4)
C(61)-C(62)-C(63)	120.6(5)
C(58)-C(63)-C(62)	120.9(4)
C(69)-C(64)-C(65)	118.6(3)
C(69)-C(64)-P(51)	118.9(2)
C(65)-C(64)-P(51)	122.5(2)
C(66)-C(65)-C(64)	120.2(3)
C(67)-C(66)-C(65)	120.9(3)
C(66)-C(67)-C(68)	119.5(3)
C(67)-C(68)-C(69)	120.1(3)
C(68)-C(69)-C(64)	120.8(3)
C(71)-C(70)-C(75)	118.6(3)
C(71)-C(70)-P(57)	123.4(2)
C(75)-C(70)-P(57)	118.0(2)

C(72)-C(71)-C(70)	120.5(3)
C(73)-C(72)-C(71)	120.2(3)
C(74)-C(73)-C(72)	120.1(3)
C(73)-C(74)-C(75)	119.9(3)
C(74)-C(75)-C(70)	120.8(3)
C(81)-C(76)-C(77)	118.7(3)
C(81)-C(76)-P(57)	121.7(2)
C(77)-C(76)-P(57)	119.4(2)
C(78)-C(77)-C(76)	120.6(3)
C(79)-C(78)-C(77)	120.3(3)
C(80)-C(79)-C(78)	119.5(3)
C(79)-C(80)-C(81)	121.0(3)
C(76)-C(81)-C(80)	120.0(3)
O(87)-C(86)-Mn(2)	178.8(3)
O(89)-C(88)-Mn(2)	174.8(3)

Supplementary Table 7: Anisotropic displacement parameters (Å^{2}x 10^3) for 1.

The anisotropic displacement factor exponent takes the form: $-2\pi^2$[$h^2a^{*2}U^{11}$ + ... + 2 h k a* b* U^{12}]

	U^{11}	U^{22}	U^{33}	U^{23}	U^{13}	U^{12}
Mn(1)	9(1)	13(1)	12(1)	1(1)	-3(1)	-2(1)
Br(1)	26(1)	15(1)	21(1)	-2(1)	-7(1)	0(1)
P(1)	13(1)	18(1)	12(1)	2(1)	-2(1)	0(1)
C(2)	16(2)	26(3)	26(2)	7(3)	-1(1)	-2(1)
Si(1)	10(1)	38(2)	19(1)	5(1)	-1(1)	3(1)
C(32)	17(2)	99(9)	50(4)	46(5)	-13(2)	-17(3)
C(33)	64(7)	57(6)	29(2)	-10(4)	-11(3)	40(5)
C(2')	16(2)	26(3)	26(2)	7(3)	-1(1)	-2(1)
Si(1')	10(1)	38(2)	19(1)	5(1)	-1(1)	3(1)
C(32')	17(2)	99(9)	50(4)	46(5)	-13(2)	-17(3)
C(33')	64(7)	57(6)	29(2)	-10(4)	-11(3)	40(5)
N(4)	10(1)	19(1)	20(1)	4(1)	-4(1)	-1(1)
Si(2)	13(1)	19(1)	39(1)	9(1)	-6(1)	2(1)
C(6)	16(1)	34(2)	30(2)	16(1)	-12(1)	-5(1)
P(7)	13(1)	23(1)	19(1)	9(1)	-6(1)	-4(1)
C(8)	30(2)	20(1)	14(1)	1(1)	-8(1)	10(1)
C(9)	36(2)	51(2)	21(2)	-6(2)	-11(1)	23(2)
C(10)	76(3)	49(2)	23(2)	-17(2)	-15(2)	35(2)

C(11)	87(4)	24(2)	32(2)	-6(2)	-34(2)	7(2)
C(12)	73(3)	32(2)	34(2)	11(2)	-35(2)	-20(2)
C(13)	44(2)	28(2)	20(2)	5(1)	-14(1)	-14(2)
C(14)	24(2)	17(1)	18(1)	2(1)	-5(1)	-1(1)
C(15)	39(2)	34(2)	25(2)	11(2)	3(2)	13(2)
C(16)	57(3)	43(2)	25(2)	10(2)	6(2)	30(2)
C(17)	51(2)	25(2)	28(2)	1(1)	-9(2)	15(2)
C(18)	47(2)	29(2)	44(2)	20(2)	5(2)	5(2)
C(19)	39(2)	33(2)	37(2)	14(2)	11(2)	9(2)
C(20)	19(2)	21(2)	29(3)	12(2)	-3(2)	-4(2)
C(21)	22(2)	32(2)	30(3)	12(2)	-8(2)	-8(1)
C(22)	27(2)	43(2)	54(3)	20(2)	-15(2)	-22(2)
C(23)	59(4)	35(3)	93(8)	36(4)	-27(4)	-27(3)
C(24)	48(4)	27(3)	83(8)	22(4)	-23(5)	-10(3)
C(25)	25(3)	22(3)	52(5)	13(3)	-8(3)	-3(2)
C(20')	19(2)	21(2)	29(3)	12(2)	-3(2)	-4(2)
C(21')	22(2)	32(2)	30(3)	12(2)	-8(2)	-8(1)
C(22')	27(2)	43(2)	54(3)	20(2)	-15(2)	-22(2)
C(23')	59(4)	35(3)	93(8)	36(4)	-27(4)	-27(3)
C(24')	48(4)	27(3)	83(8)	22(4)	-23(5)	-10(3)
C(25')	25(3)	22(3)	52(5)	13(3)	-8(3)	-3(2)
C(26)	24(2)	39(2)	16(2)	8(1)	-3(1)	-10(1)
C(27)	39(2)	69(3)	22(2)	15(2)	-11(2)	-10(2)

C(28)	56(3)	93(4)	14(2)	6(2)	-6(2)	-22(3)
C(29)	44(3)	92(4)	22(2)	-11(2)	4(2)	-12(2)
C(30)	39(2)	75(3)	26(2)	-13(2)	2(2)	4(2)
C(31)	28(2)	53(2)	19(2)	-4(2)	-4(1)	5(2)
C(34)	16(2)	44(2)	64(3)	21(2)	-14(2)	0(2)
C(35)	39(2)	19(2)	79(3)	-1(2)	-8(2)	6(2)
C(36)	14(1)	22(2)	21(2)	3(1)	-4(1)	-1(1)
O(37)	47(2)	11(1)	22(1)	-6(1)	4(1)	-3(1)
C(38)	17(1)	22(1)	11(1)	4(1)	-3(1)	-4(1)
O(39)	14(1)	44(1)	24(1)	5(1)	-4(1)	-1(1)
Mn(2)	11(1)	16(1)	12(1)	1(1)	-3(1)	-1(1)
Br(2)	27(1)	20(1)	24(1)	-4(1)	0(1)	1(1)
P(51)	17(1)	18(1)	16(1)	4(1)	-6(1)	-5(1)
C(52)	18(2)	26(2)	15(3)	6(2)	-10(2)	-6(1)
C(82)	62(6)	46(6)	23(5)	7(3)	-25(4)	-32(4)
C(83)	18(2)	67(6)	54(6)	41(5)	-1(3)	6(2)
C(52')	18(2)	26(2)	15(3)	6(2)	-10(2)	-6(1)
C(82')	62(6)	46(6)	23(5)	7(3)	-25(4)	-32(4)
C(83')	18(2)	67(6)	54(6)	41(5)	-1(3)	6(2)
Si(4)	14(1)	22(1)	29(1)	9(1)	-7(1)	-4(1)
C(56)	11(1)	22(2)	23(2)	3(1)	0(1)	0(1)
P(57)	11(1)	19(1)	12(1)	1(1)	-3(1)	0(1)
C(58)	54(2)	24(2)	14(2)	4(1)	-4(2)	-18(2)

C(59)	68(3)	76(3)	30(2)	-5(2)	-10(2)	-42(3)
C(60)	86(4)	56(3)	35(2)	-13(2)	-8(2)	-42(3)
C(61)	92(4)	34(2)	39(3)	-8(2)	13(3)	-20(2)
C(62)	85(4)	34(2)	26(2)	7(2)	18(2)	10(2)
C(63)	63(3)	30(2)	19(2)	4(1)	6(2)	13(2)
C(64)	13(1)	16(1)	20(1)	0(1)	1(1)	0(1)
C(65)	22(2)	20(2)	27(2)	7(1)	-9(1)	-7(1)
C(66)	25(2)	31(2)	39(2)	9(2)	-16(2)	-13(1)
C(67)	21(2)	21(2)	38(2)	3(1)	-4(1)	-6(1)
C(68)	27(2)	17(1)	26(2)	5(1)	-1(1)	1(1)
C(69)	24(2)	20(1)	19(2)	2(1)	-6(1)	-4(1)
C(70)	19(1)	20(1)	12(1)	1(1)	-6(1)	1(1)
C(71)	22(2)	23(2)	29(2)	1(1)	-10(1)	-1(1)
C(72)	36(2)	23(2)	40(2)	-4(2)	-18(2)	-2(1)
C(73)	39(2)	22(2)	27(2)	-5(1)	-11(2)	8(1)
C(74)	24(2)	30(2)	21(2)	-4(1)	-6(1)	8(1)
C(75)	20(2)	26(2)	18(1)	0(1)	-4(1)	2(1)
C(76)	14(1)	22(1)	16(1)	-2(1)	-5(1)	2(1)
C(77)	27(2)	36(2)	15(2)	-2(1)	-2(1)	-4(1)
C(78)	40(2)	51(2)	14(2)	-1(2)	-3(1)	-5(2)
C(79)	33(2)	43(2)	20(2)	-10(2)	-10(1)	2(2)
C(80)	23(2)	39(2)	28(2)	-7(2)	-7(1)	-4(1)
C(81)	21(2)	32(2)	18(2)	-3(1)	-3(1)	-3(1)

C(84)	39(2)	30(2)	34(2)	9(2)	-16(2)	-14(2)
C(85)	12(2)	42(2)	52(2)	21(2)	-6(2)	0(1)
C(86)	23(2)	23(2)	17(1)	6(1)	-4(1)	-3(1)
O(87)	79(2)	18(1)	15(1)	-1(1)	-16(1)	-16(1)
C(88)	21(2)	21(1)	14(1)	5(1)	-2(1)	-2(1)
O(89)	15(1)	43(2)	28(1)	7(1)	-1(1)	0(1)

Supplementary Table 8: Hydrogen coordinates (x 10^4) and isotropic displacement parameters ($Å^2$x 10^3) for 1.

	x	y	z	U(eq)
H(2A)	1894	8037	1471	28
H(2B)	2037	7625	791	28
H(32A)	5089	6754	1726	82
H(32B)	4370	7601	1891	82
H(32C)	4893	7376	1168	82
H(33A)	2233	5660	946	76
H(33B)	3665	5468	1139	76
H(33C)	3524	6061	557	76
H(2'1)	2053	7909	1410	28
H(2'2)	2063	7470	750	28
H(32D)	4650	7297	1751	82
H(32E)	5040	6940	1055	82
H(32F)	5081	6377	1665	82
H(33D)	1933	5512	1003	76
H(33E)	3360	5242	1166	76
H(33F)	3254	5799	559	76
H(4)	2171	6857	2544	19
H(6A)	1357	5207	3746	31
H(6B)	1542	6152	3717	31

H(9)	1469	6686	175	43
H(10)	800	6020	-668	58
H(11)	-1405	5673	-619	54
H(12)	-2973	6057	236	52
H(13)	-2325	6699	1093	35
H(15)	-2171	8121	2152	41
H(16)	-3345	9303	2002	53
H(17)	-2705	10108	1126	42
H(18)	-942	9700	361	50
H(19)	212	8506	500	46
H(21)	-2917	5271	3297	33
H(22)	-3953	4041	3369	48
H(23)	-2703	2864	3454	73
H(24)	-418	2918	3468	62
H(25)	619	4148	3396	40
H(21')	-2933	5243	3116	33
H(22')	-3956	4010	3264	48
H(23')	-2834	2917	3649	73
H(24')	-690	3056	3887	62
H(25')	332	4290	3739	40
H(27)	-153	5864	4660	51
H(28)	-1447	6433	5558	65
H(29)	-3374	7157	5476	64

H(30)	-3999	7375	4484	57
H(31)	-2709	6846	3579	40
H(34A)	4846	5593	2518	62
H(34B)	4368	5247	3218	62
H(34C)	4313	6187	3084	62
H(35A)	1520	4606	2183	69
H(35B)	2512	4189	2608	69
H(35C)	3089	4606	1945	69
H(52A)	2467	2685	861	22
H(52B)	2207	3142	1524	22
H(54)	1397	2022	2630	16
H(82A)	1960	761	1026	62
H(82B)	914	1263	681	62
H(82C)	401	685	1268	62
H(83A)	-434	2877	2044	71
H(83B)	-1171	2078	1929	71
H(83C)	-634	2650	1342	71
H(52C)	2324	2909	1054	22
H(52D)	2336	3221	1756	22
H(54')	1385	2074	2797	16
H(82D)	1622	963	1137	62
H(82E)	592	1531	836	62
H(82F)	71	942	1414	62

H(83D)	-1280	2274	2189	71
H(83E)	-810	2924	1648	71
H(83F)	-476	3028	2349	71
H(56A)	1461	1175	3858	23
H(56B)	1477	224	3810	23
H(59)	3226	1777	273	68
H(60)	4383	1069	-628	70
H(61)	6582	685	-661	69
H(62)	7727	1051	121	62
H(63)	6634	1691	1026	47
H(65)	5959	2986	2201	27
H(66)	7267	4109	2056	36
H(67)	7165	4974	1202	32
H(68)	5765	4701	468	28
H(69)	4475	3569	597	25
H(71)	2224	-842	3302	29
H(72)	3012	-2161	3283	38
H(73)	5275	-2446	3233	35
H(74)	6761	-1413	3205	30
H(75)	5988	-92	3206	25
H(77)	2870	443	4633	31
H(78)	3872	705	5515	42
H(79)	5861	1382	5392	37

H(80)	6810	1827	4393	36
H(81)	5781	1619	3509	29
H(84A)	136	-27	2118	50
H(84B)	423	-546	2721	50
H(84C)	1634	-235	2214	50
H(85A)	-963	1614	3457	54
H(85B)	-1386	702	3498	54
H(85C)	-1481	1218	2868	54

Crystal data and structure refinement for (IIa)

Summary of Data CCDC 1868571

Identification code	11666
Empirical formula	C39 H51 B Cl2 Mn N O4 P2 Si2
Formula weight	852.58
Temperature	100(2) K
Wavelength	0.71073 Å
Crystal system	Monoclinic
Space group	P 2_1/n , No. 14
Unit cell dimensions	a = 10.7001(11) Å α= 90°.
	b = 9.8564(11) Å β= 96.519(8)°.
	c = 39.297(5) Å γ = 90°.
Volume	4117.6(8) Å^3
Z	4
Density (calculated)	1.375 Mg/m^3
Absorption coefficient	0.627 mm^{-1}
F(000)	1784
Crystal size	0.080 x 0.050 x 0.040 mm^3
Theta range for data collection	2.818 to 29.000°.
Index ranges	-14<=h<=14, -13<=k<=13, -52<=l<=53
Reflections collected	55882
Independent reflections	10905 [R(int) = 0.0870]
Completeness to theta = 25.242°	99.7 %

Absorption correction	Gaussian
Max. and min. transmission	0.97764 and 0.95663
Refinement method	Full-matrix least-squares on F^2
Data / restraints / parameters	10905 / 0 / 480
Goodness-of-fit on F^2	1.054
Final R indices [I>2sigma(I)]	R1 = 0.0553, wR2 = 0.0966
R indices (all data)	R1 = 0.1010, wR2 = 0.1123
Extinction coefficient	n/a
Largest diff. peak and hole	1.282 and -0.814 e.Å^{-3}

Supplementary Table 9: Atomic coordinates (x 10^4) and equivalent isotropic displacement parameters ($Å^2$x 10^3) for 11666.

U(eq) is defined as one third of the trace of the orthogonalized U^{ij} tensor.

	x	y	z	U(eq)
Mn(1)	5201(1)	5764(1)	1271(1)	10(1)
N(1)	4627(2)	3640(2)	1212(1)	12(1)
Si(2)	5023(1)	2623(1)	1583(1)	13(1)
C(3)	4895(3)	3780(3)	1953(1)	14(1)
P(4)	5382(1)	5482(1)	1847(1)	12(1)
Si(5)	5031(1)	3042(1)	811(1)	13(1)
C(6)	4568(3)	4385(3)	483(1)	15(1)
P(7)	4739(1)	6037(1)	697(1)	11(1)
B(8)	3329(3)	4316(3)	1205(1)	14(1)
O(9)	2550(2)	4364(2)	888(1)	14(1)
C(10)	1268(2)	4101(3)	950(1)	15(1)
C(11)	1299(2)	4408(3)	1341(1)	14(1)
O(12)	2571(2)	4015(2)	1470(1)	14(1)
C(13)	987(3)	2610(3)	865(1)	22(1)
C(14)	390(3)	4994(3)	717(1)	21(1)
C(15)	384(3)	3579(3)	1522(1)	21(1)
C(16)	1135(3)	5911(3)	1416(1)	18(1)
C(17)	3916(3)	1178(3)	1611(1)	19(1)

C(18)	6656(3)	1936(3)	1608(1)	19(1)
C(19)	4242(3)	1403(3)	695(1)	22(1)
C(20)	6754(3)	2722(3)	804(1)	20(1)
C(21)	4470(3)	6537(3)	2114(1)	16(1)
C(22)	4469(3)	6225(3)	2460(1)	23(1)
C(23)	3746(3)	6956(3)	2664(1)	27(1)
C(24)	3016(3)	8025(3)	2528(1)	26(1)
C(25)	3006(3)	8343(3)	2187(1)	23(1)
C(26)	3711(3)	7599(3)	1979(1)	18(1)
C(31)	6981(3)	5693(3)	2067(1)	16(1)
C(32)	7681(3)	4640(3)	2224(1)	21(1)
C(33)	8923(3)	4843(3)	2366(1)	26(1)
C(34)	9470(3)	6098(3)	2349(1)	25(1)
C(35)	8780(3)	7163(3)	2194(1)	26(1)
C(36)	7536(3)	6964(3)	2056(1)	22(1)
C(41)	3311(2)	7000(3)	547(1)	14(1)
C(42)	2743(3)	6852(3)	211(1)	20(1)
C(43)	1692(3)	7621(3)	96(1)	24(1)
C(44)	1208(3)	8542(3)	312(1)	24(1)
C(45)	1766(3)	8700(3)	644(1)	21(1)
C(46)	2817(3)	7925(3)	760(1)	16(1)
C(51)	5908(2)	6933(3)	474(1)	13(1)
C(52)	7010(3)	6288(3)	404(1)	16(1)

C(53)	7883(3)	6957(3)	232(1)	20(1)
C(54)	7687(3)	8295(3)	134(1)	22(1)
C(55)	6622(3)	8965(3)	212(1)	20(1)
C(56)	5740(3)	8289(3)	380(1)	16(1)
C(61)	5432(2)	7525(3)	1313(1)	14(1)
O(62)	5650(2)	8687(2)	1337(1)	22(1)
C(71)	6860(3)	5638(3)	1253(1)	15(1)
O(72)	7939(2)	5657(2)	1248(1)	22(1)
C(80)	-1099(3)	8962(4)	1408(1)	30(1)
Cl(81)	438(1)	9534(1)	1554(1)	52(1)
Cl(82)	-1430(1)	9182(1)	965(1)	39(1)

Supplementary Table 10: Bond lengths [Å] and angles [°] for 11666.

Mn(1)-H(8)	1.58(3)
Mn(1)-C(61)	1.758(3)
Mn(1)-C(71)	1.789(3)
Mn(1)-N(1)	2.187(2)
Mn(1)-P(4)	2.2693(8)
Mn(1)-P(7)	2.2695(8)
N(1)-B(8)	1.537(4)
N(1)-Si(5)	1.779(2)
N(1)-Si(2)	1.781(2)
Si(2)-C(17)	1.864(3)
Si(2)-C(3)	1.865(3)
Si(2)-C(18)	1.865(3)
C(3)-P(4)	1.819(3)
P(4)-C(21)	1.834(3)
P(4)-C(31)	1.839(3)
Si(5)-C(19)	1.856(3)
Si(5)-C(20)	1.873(3)
Si(5)-C(6)	1.875(3)
C(6)-P(7)	1.832(3)
P(7)-C(51)	1.833(3)
P(7)-C(41)	1.837(3)
B(8)-O(9)	1.419(3)

B(8)-O(12)	1.422(4)
B(8)-H(8)	1.55(3)
O(9)-C(10)	1.444(3)
C(10)-C(14)	1.518(4)
C(10)-C(13)	1.529(4)
C(10)-C(11)	1.560(4)
C(11)-O(12)	1.451(3)
C(11)-C(15)	1.515(4)
C(11)-C(16)	1.524(4)
C(21)-C(26)	1.393(4)
C(21)-C(22)	1.393(4)
C(22)-C(23)	1.381(4)
C(23)-C(24)	1.383(4)
C(24)-C(25)	1.375(4)
C(25)-C(26)	1.384(4)
C(31)-C(32)	1.382(4)
C(31)-C(36)	1.389(4)
C(32)-C(33)	1.396(4)
C(33)-C(34)	1.373(5)
C(34)-C(35)	1.384(4)
C(35)-C(36)	1.393(4)
C(41)-C(46)	1.383(4)
C(41)-C(42)	1.400(4)

C(42)-C(43)	1.389(4)
C(43)-C(44)	1.383(5)
C(44)-C(45)	1.382(4)
C(45)-C(46)	1.393(4)
C(51)-C(56)	1.393(4)
C(51)-C(52)	1.394(4)
C(52)-C(53)	1.382(4)
C(53)-C(54)	1.384(4)
C(54)-C(55)	1.382(4)
C(55)-C(56)	1.381(4)
C(61)-O(62)	1.170(3)
C(71)-O(72)	1.158(3)
C(80)-Cl(82)	1.748(3)
C(80)-Cl(81)	1.771(3)

H(8)-Mn(1)-C(61)	95.9(10)
H(8)-Mn(1)-C(71)	177.1(11)
C(61)-Mn(1)-C(71)	86.73(12)
H(8)-Mn(1)-N(1)	76.1(10)
C(61)-Mn(1)-N(1)	171.85(10)
C(71)-Mn(1)-N(1)	101.35(11)
H(8)-Mn(1)-P(4)	85.4(10)
C(61)-Mn(1)-P(4)	91.82(9)

C(71)-Mn(1)-P(4)	93.28(8)
N(1)-Mn(1)-P(4)	88.85(6)
H(8)-Mn(1)-P(7)	87.0(10)
C(61)-Mn(1)-P(7)	89.39(8)
C(71)-Mn(1)-P(7)	94.30(8)
N(1)-Mn(1)-P(7)	88.90(6)
P(4)-Mn(1)-P(7)	172.38(3)
B(8)-N(1)-Si(5)	116.01(17)
B(8)-N(1)-Si(2)	112.86(18)
Si(5)-N(1)-Si(2)	118.61(13)
B(8)-N(1)-Mn(1)	80.15(15)
Si(5)-N(1)-Mn(1)	108.51(11)
Si(2)-N(1)-Mn(1)	114.44(11)
N(1)-Si(2)-C(17)	112.62(12)
N(1)-Si(2)-C(3)	105.24(11)
C(17)-Si(2)-C(3)	108.50(13)
N(1)-Si(2)-C(18)	112.54(12)
C(17)-Si(2)-C(18)	108.46(14)
C(3)-Si(2)-C(18)	109.35(13)
P(4)-C(3)-Si(2)	109.75(14)
C(3)-P(4)-C(21)	102.01(13)
C(3)-P(4)-C(31)	105.81(13)
C(21)-P(4)-C(31)	101.23(12)

C(3)-P(4)-Mn(1)	110.27(9)
C(21)-P(4)-Mn(1)	120.67(9)
C(31)-P(4)-Mn(1)	115.14(9)
N(1)-Si(5)-C(19)	110.85(12)
N(1)-Si(5)-C(20)	114.04(12)
C(19)-Si(5)-C(20)	105.72(14)
N(1)-Si(5)-C(6)	107.63(11)
C(19)-Si(5)-C(6)	111.59(13)
C(20)-Si(5)-C(6)	107.01(13)
P(7)-C(6)-Si(5)	107.86(13)
C(6)-P(7)-C(51)	104.30(13)
C(6)-P(7)-C(41)	106.06(12)
C(51)-P(7)-C(41)	100.71(12)
C(6)-P(7)-Mn(1)	110.49(9)
C(51)-P(7)-Mn(1)	116.62(8)
C(41)-P(7)-Mn(1)	117.28(9)
O(9)-B(8)-O(12)	108.8(2)
O(9)-B(8)-N(1)	118.0(2)
O(12)-B(8)-N(1)	119.1(2)
O(9)-B(8)-H(8)	106.6(11)
O(12)-B(8)-H(8)	101.7(11)
N(1)-B(8)-H(8)	100.0(11)
B(8)-O(9)-C(10)	108.6(2)

O(9)-C(10)-C(14)	109.1(2)
O(9)-C(10)-C(13)	107.6(2)
C(14)-C(10)-C(13)	109.4(2)
O(9)-C(10)-C(11)	102.6(2)
C(14)-C(10)-C(11)	115.0(2)
C(13)-C(10)-C(11)	112.6(2)
O(12)-C(11)-C(15)	108.7(2)
O(12)-C(11)-C(16)	108.4(2)
C(15)-C(11)-C(16)	109.8(2)
O(12)-C(11)-C(10)	101.9(2)
C(15)-C(11)-C(10)	114.6(2)
C(16)-C(11)-C(10)	112.9(2)
B(8)-O(12)-C(11)	106.2(2)
C(26)-C(21)-C(22)	118.3(3)
C(26)-C(21)-P(4)	122.2(2)
C(22)-C(21)-P(4)	119.4(2)
C(23)-C(22)-C(21)	121.1(3)
C(22)-C(23)-C(24)	120.0(3)
C(25)-C(24)-C(23)	119.6(3)
C(24)-C(25)-C(26)	120.7(3)
C(25)-C(26)-C(21)	120.4(3)
C(32)-C(31)-C(36)	118.7(3)
C(32)-C(31)-P(4)	123.5(2)

C(36)-C(31)-P(4)	117.7(2)
C(31)-C(32)-C(33)	120.9(3)
C(34)-C(33)-C(32)	120.1(3)
C(33)-C(34)-C(35)	119.7(3)
C(34)-C(35)-C(36)	120.2(3)
C(31)-C(36)-C(35)	120.5(3)
C(46)-C(41)-C(42)	119.1(3)
C(46)-C(41)-P(7)	120.5(2)
C(42)-C(41)-P(7)	120.3(2)
C(43)-C(42)-C(41)	120.0(3)
C(44)-C(43)-C(42)	120.2(3)
C(45)-C(44)-C(43)	120.2(3)
C(44)-C(45)-C(46)	119.6(3)
C(41)-C(46)-C(45)	120.9(3)
C(56)-C(51)-C(52)	118.1(3)
C(56)-C(51)-P(7)	121.0(2)
C(52)-C(51)-P(7)	120.8(2)
C(53)-C(52)-C(51)	120.8(3)
C(52)-C(53)-C(54)	120.2(3)
C(55)-C(54)-C(53)	119.7(3)
C(56)-C(55)-C(54)	120.1(3)
C(55)-C(56)-C(51)	121.0(3)
O(62)-C(61)-Mn(1)	176.5(3)

O(72)-C(71)-Mn(1)	174.9(2)
Cl(82)-C(80)-Cl(81)	111.1(2)

Supplementary Table 11: Anisotropic displacement parameters (Å^{2}x 10^3) for 11666.

The anisotropic displacement factor exponent takes the form: $-2\pi^2[h^2 a^{*2}U^{11} + ... + 2 h k a^* b^* U^{12}]$

	U^{11}	U^{22}	U^{33}	U^{23}	U^{13}	U^{12}
Mn(1)	10(1)	11(1)	10(1)	0(1)	0(1)	0(1)
N(1)	12(1)	12(1)	11(1)	0(1)	1(1)	0(1)
Si(2)	13(1)	13(1)	14(1)	2(1)	1(1)	2(1)
C(3)	12(1)	16(1)	12(1)	3(1)	0(1)	-1(1)
P(4)	13(1)	13(1)	10(1)	0(1)	0(1)	1(1)
Si(5)	16(1)	12(1)	12(1)	-1(1)	1(1)	2(1)
C(6)	16(1)	14(1)	14(1)	-3(1)	1(1)	0(1)
P(7)	12(1)	12(1)	11(1)	0(1)	1(1)	2(1)
B(8)	16(2)	13(2)	14(1)	1(1)	2(1)	2(1)
O(9)	10(1)	18(1)	14(1)	1(1)	1(1)	-1(1)
C(10)	10(1)	18(1)	16(1)	2(1)	1(1)	-2(1)
C(11)	7(1)	18(1)	18(1)	1(1)	0(1)	1(1)
O(12)	10(1)	19(1)	13(1)	2(1)	-1(1)	2(1)
C(13)	19(2)	21(2)	25(2)	-4(1)	0(1)	-6(1)
C(14)	13(1)	27(2)	23(2)	4(1)	-2(1)	-1(1)
C(15)	17(2)	23(2)	23(2)	4(1)	4(1)	1(1)
C(16)	13(1)	18(2)	22(1)	1(1)	2(1)	0(1)
C(17)	23(2)	15(1)	20(2)	3(1)	3(1)	1(1)

C(18)	19(2)	20(2)	17(1)	1(1)	-2(1)	5(1)
C(19)	31(2)	15(2)	18(2)	-3(1)	2(1)	0(1)
C(20)	22(2)	22(2)	18(1)	0(1)	4(1)	9(1)
C(21)	15(1)	16(1)	16(1)	-2(1)	1(1)	-3(1)
C(22)	35(2)	19(2)	16(1)	-1(1)	5(1)	1(1)
C(23)	40(2)	26(2)	17(2)	-2(1)	11(1)	-5(2)
C(24)	23(2)	30(2)	26(2)	-9(1)	12(1)	-1(1)
C(25)	18(2)	26(2)	26(2)	-5(1)	3(1)	4(1)
C(26)	16(1)	23(2)	16(1)	-3(1)	2(1)	1(1)
C(31)	18(1)	22(2)	8(1)	-2(1)	1(1)	0(1)
C(32)	18(2)	21(2)	24(2)	-2(1)	0(1)	1(1)
C(33)	18(2)	33(2)	27(2)	-2(1)	-6(1)	5(1)
C(34)	16(2)	38(2)	20(2)	-9(1)	-3(1)	-3(1)
C(35)	28(2)	33(2)	17(2)	-3(1)	-4(1)	-11(1)
C(36)	26(2)	24(2)	14(1)	2(1)	-4(1)	-4(1)
C(41)	13(1)	14(1)	15(1)	3(1)	1(1)	0(1)
C(42)	19(2)	24(2)	16(1)	3(1)	0(1)	2(1)
C(43)	17(2)	33(2)	22(2)	10(1)	-4(1)	-1(1)
C(44)	13(1)	22(2)	35(2)	12(1)	-1(1)	3(1)
C(45)	14(1)	16(2)	34(2)	2(1)	6(1)	3(1)
C(46)	14(1)	15(1)	18(1)	1(1)	1(1)	0(1)
C(51)	13(1)	19(1)	8(1)	-1(1)	-1(1)	-2(1)
C(52)	16(1)	18(1)	15(1)	1(1)	-1(1)	2(1)

C(53)	14(1)	28(2)	17(1)	0(1)	1(1)	-1(1)
C(54)	19(2)	32(2)	17(1)	6(1)	1(1)	-9(1)
C(55)	20(2)	21(2)	19(1)	5(1)	-1(1)	-4(1)
C(56)	16(1)	18(1)	15(1)	1(1)	-1(1)	0(1)
C(61)	11(1)	20(2)	10(1)	2(1)	-2(1)	2(1)
O(62)	24(1)	13(1)	27(1)	-2(1)	1(1)	-3(1)
C(71)	21(2)	14(1)	9(1)	-1(1)	0(1)	2(1)
O(72)	12(1)	30(1)	25(1)	-2(1)	0(1)	1(1)
C(80)	32(2)	30(2)	26(2)	-1(1)	-3(1)	7(2)
Cl(81)	60(1)	34(1)	52(1)	1(1)	-30(1)	-12(1)
Cl(82)	32(1)	58(1)	26(1)	0(1)	-4(1)	-3(1)

Supplementary Table 12: Hydrogen coordinates (x 10^4) and isotropic displacement parameters (Å^{2}x 10 ^{3}) for 11666.

	x	y	z	U(eq)
H(3A)	4015	3798	2008	17
H(3B)	5436	3443	2156	17
H(6A)	5115	4339	296	18
H(6B)	3686	4249	383	18
H(8)	3740(30)	5800(30)	1293(7)	17
H(13A)	1151	2426	629	33
H(13B)	102	2418	889	33
H(13C)	1526	2031	1023	33
H(14A)	660	5942	744	32
H(14B)	-469	4904	778	32
H(14C)	411	4713	479	32
H(15A)	569	2612	1499	31
H(15B)	-477	3765	1420	31
H(15C)	470	3824	1766	31
H(16A)	1329	6073	1662	27
H(16B)	264	6181	1343	27
H(16C)	1706	6446	1291	27
H(17A)	3064	1529	1621	29

H(17B)	4176	649	1818	29
H(17C)	3926	596	1409	29
H(18A)	6696	1237	1433	29
H(18B)	6888	1538	1835	29
H(18C)	7241	2671	1571	29
H(19A)	4601	693	851	32
H(19B)	4368	1165	459	32
H(19C)	3340	1487	714	32
H(20A)	7235	3469	920	31
H(20B)	6942	2663	566	31
H(20C)	6985	1868	923	31
H(22)	4973	5498	2556	28
H(23)	3750	6724	2899	32
H(24)	2525	8536	2669	31
H(25)	2509	9081	2093	28
H(26)	3677	7814	1742	22
H(32)	7312	3767	2235	26
H(33)	9390	4113	2475	32
H(34)	10319	6234	2442	30
H(35)	9156	8032	2182	32
H(36)	7063	7703	1953	26
H(42)	3077	6226	61	24
H(43)	1303	7514	-132	29

H(44)	490	9067	232	29
H(45)	1435	9334	792	25
H(46)	3199	8032	989	19
H(52)	7163	5377	476	20
H(53)	8618	6497	181	23
H(54)	8282	8752	13	27
H(55)	6496	9891	151	24
H(56)	5008	8755	431	20
H(80A)	-1175	7989	1464	36
H(80B)	-1719	9473	1526	36

Supplementary References

1 Fulmer, G. R. *et al.* NMR Chemical Shifts of Trace Impurities: Common Laboratory Solvents, Organics, and Gases in Deuterated Solvents Relevant to the Organometallic Chemist. *Organometallics* **29**, 2176-2179, doi:10.1021/om100106e (2010).

2 Fryzuk, M. D., MacNeil, P. A., Rettig, S. J., Secco, A. S. & Trotter, J. Tridentate amidophosphine derivatives of the nickel triad: synthesis, characterization, and reactivity of nickel(II), palladium(II), and platinum(II) amide complexes. *Organometallics* **1**, 918-930, doi:10.1021/om00067a006 (1982).

3 Nave, S., Sonawane, R. P., Elford, T. G. & Aggarwal, V. K. Protodeboronation of tertiary boronic esters: asymmetric synthesis of tertiary alkyl stereogenic centers. *J. Am. Chem. Soc.* **132**, 17096-17098, doi:10.1021/ja1084207 (2010).

4 Boudet, N., Lachs, J. R. & Knochel, P. Multiple regioselective functionalizations of quinolines via magnesiations. *Org. Lett.* **9**, 5525-5528, doi:10.1021/ol702494k (2007).

5 Kisan, S., Krishnakumar, V. & Gunanathan, C. Ruthenium-Catalyzed Deoxygenative Hydroboration of Carboxylic Acids. *ACS Catal.* **8**, 4772-4776, doi:10.1021/acscatal.8b00900 (2018).

6 Barman, M. K., Baishya, A. & Nembenna, S. Magnesium amide catalyzed selective hydroboration of esters. *Dalton Trans.* **46**, 4152-4156, doi:10.1039/c7dt00556c (2017).

7 Mukherjee, D., Osseili, H., Truong, K. N., Spaniol, T. P. & Okuda, J. Ring-opening of cyclic ethers by aluminum hydridotriphenylborate. *Chem. Commun.* **53**, 3493-3496, doi:10.1039/c7cc01159h (2017).

8 Romero, E. A., Peltier, J. L., Jazzar, R. & Bertrand, G. Catalyst-free dehydrocoupling of amines, alcohols, and thiols with pinacol borane and 9-borabicyclononane (9-BBN). *Chem. Commun.* **52**, 10563-10565, doi:10.1039/c6cc06096j (2016).

9 Lu, L. Q., Li, Y., Junge, K. & Beller, M. Iron-catalyzed hydrogenation for the in situ regeneration of an NAD(P)H model: biomimetic reduction of alpha-keto-/alpha-iminoesters. *Angew. Chem. Int. Ed.* **52**, 8382-8386, doi:10.1002/anie.201301972 (2013).

10 Pollard, V. A. *et al.* Lithium diamidodihydridoaluminates: bimetallic cooperativity in catalytic hydroboration and metallation applications. *Chem. Commun.* **54**, 1233-1236, doi:10.1039/c7cc08214b (2018).

11 Zhu, Z. *et al.* Lanthanide aryloxides catalyzed hydroboration of aldehydes and ketones. *Catal. Comm.* **112**, 26-30, doi:10.1016/j.catcom.2018.04.014 (2018).

12 Mukherjee, D., Ellern, A. & Sadow, A. D. Magnesium-catalyzed hydroboration of esters: evidence for a new zwitterionic mechanism. *Chem. Sci.* **5**, 959-964, doi:10.1039/c3sc52793j (2014).

13 Kaithal, A., Chatterjee, B. & Gunanathan, C. Ruthenium Catalyzed Selective Hydroboration of Carbonyl Compounds. *Org. Lett.* **17**, 4790-4793, doi:10.1021/acs.orglett.5b02352 (2015).

14 Chattopadhyay, B. *et al.* Ir-Catalyzed ortho-Borylation of Phenols Directed by Substrate-Ligand Electrostatic Interactions: A Combined Experimental/in Silico Strategy for Optimizing Weak Interactions. *J. Am. Chem. Soc.* **139**, 7864-7871, doi:10.1021/jacs.7b02232 (2017).

15 Chakraborty, S., Zhang, J., Krause, J. A. & Guan, H. An efficient nickel catalyst for the reduction of carbon dioxide with a borane. *J. Am. Chem. Soc.* **132**, 8872-8873, doi:10.1021/ja103982t (2010).
